# Enantiocomplementary Gut Bacterial Enzymes Metabolize Dietary Polyphenols

**DOI:** 10.1021/jacs.4c09892

**Published:** 2025-02-24

**Authors:** Xueyang Dong, Minwoo Bae, Chi Le, Miguel A. Aguilar Ramos, Emily P. Balskus

**Affiliations:** † Department of Chemistry and Chemical Biology, Harvard University, Cambridge, Massachusetts 02138, United States; ‡ Howard Hughes Medical Institute, Harvard University, Cambridge, Massachusetts 02138, United States

## Abstract

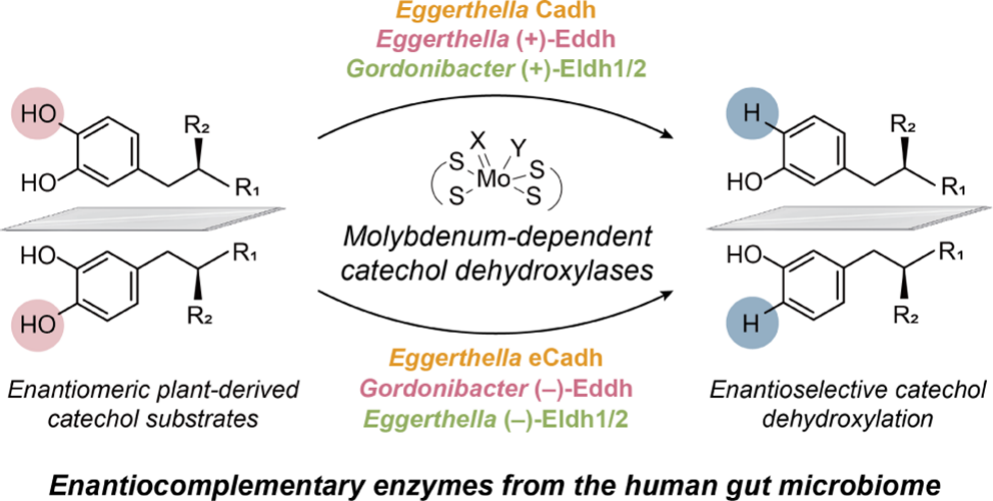

Molybdenum-dependent catechol dehydroxylases in gut Actinobacteria catalyze the removal of *para*-hydroxyl groups from catechols, a central reaction in the microbial metabolism of polyphenol compounds. However, the substrates of most putative catechol dehydroxylases remain unidentified due to the challenges of obtaining these enzymes from standard heterologous expression systems. In this work, we establish *Gordonibacter urolithinfaciens* as a versatile bacterial host to express active catechol dehydroxylases. Using this system, we rapidly deorphanize eight previously uncharacterized gut bacterial catechol dehydroxylases that selectively dehydroxylate intermediates in the gut bacterial metabolism of plant-derived catechins and lignans. Unexpectedly, we discover multiple instances of distinct catechol dehydroxylases that have evolved to selectively metabolize individual substrate enantiomers, setting the stage for future efforts to elucidate their mechanisms and evolution. Altogether, these findings greatly increase our knowledge of these metalloenzymes, illustrating the power of bacterial genetics to accelerate enzyme discovery and providing a more complete understanding of transformations relevant to the health benefits of phytochemicals.

## Introduction

Polyphenols, known for their antioxidant and anti-inflammatory properties, are an important group of phytochemicals.^1^ Consumption of foods rich in polyphenols, such as berries (anthocyanins), tea (catechins), and flaxseeds (lignans), is suggested to have protective effects against certain cancers, neurodegenerative diseases, cardiovascular diseases, and obesity, among others.^1−3^ The health benefits of polyphenols are thought to be greatly impacted by gut bacterial metabolism which alters the chemical structures of polyphenols, changing their bioactivity and bioavailability.^4^ However, the molecular determinants underlying the transformation of individual polyphenols by gut bacteria are poorly understood. Therefore, it is important to elucidate the specific gut microbial enzymes that mediate polyphenol metabolism.

**Figure 1:**
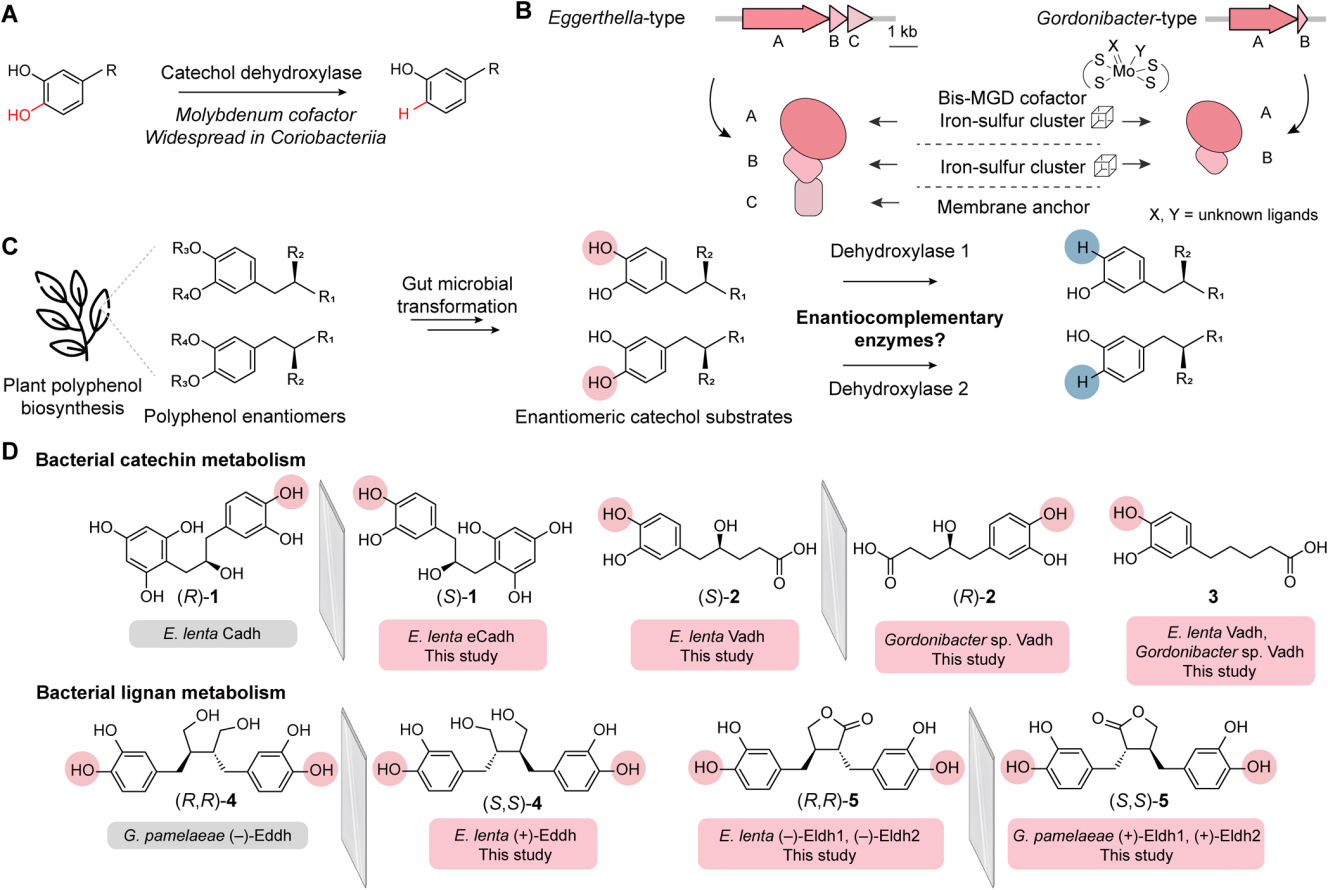
Catechol dehydroxylases metabolize dietary polyphenol enantiomers. (A) Schematic of catechol dehydroxylation. (B) Subunit organization and cofactor composition of *Eggerthella*-type and *Gordonibacter*-type catechol dehydroxylases. (C) Schematic of plant biosynthesis and gut microbial metabolism of enantiomeric dietary polyphenols. Enantiocomplementary catechol dehydroxylases would selectively transform individual catechol enantiomers. (D) Summary of catechol substrates examined in this study with their *para*–OHs highlighted. Enzymes characterized in this study and in previous studies are colored pink and gray, respectively.

One prominent transformation of polyphenols performed by the gut microbiome is dehydroxylation of catechols (compounds containing a 1,2-dihydroxylated aromatic ring) (Figure 1A). Catechol dehydroxylation occurs on a wide range of dietary phytochemicals, including hydrocaffeic acid,^4,5^ 3,4-dihydroxyphenylacetic acid (DOPAC),^4−6^ lignans,^7,8^ and catechins,^4,9−11^ as well as the host-derived catecholamine neurotransmitters dopamine and norepinephrine.^12−14^ Catechol dehydroxylation is performed by a recently discovered family of molybdenum-dependent metalloenzymes termed catechol dehydroxylases, which belongs to the larger dimethyl sulfoxide (DMSO) reductase superfamily and requires a bis-molybdopterin guanidine dinucleotide (bis-MGD) cofactor.^4,12,15^ Within the human gut microbiome, catechol dehydroxylases are encoded by Coriobacteriia, a class of anaerobic Gram-positive Actinobacteria commonly found in the mammalian gastrointestinal tract that includes *Eggerthella* and *Gordonibacter*. These two genera encode two distinct types of catechol dehydroxylases (Figure 1B). *Eggerthella*-type catechol dehydroxylases, exemplified by dopamine dehydroxylase (Dadh),^12^ are predicted to consist of three protein subunits including a bis-MGD cofactor-binding catalytic subunit, an iron–sulfur cluster-binding protein predicted to mediate electron transfer, and a predicted membrane-anchoring subunit^4,15^ (Figure 1B). By contrast, *Gordonibacter*-type catechol dehydroxylases, exemplified by DOPAC dehydroxylase (Dodh)^4^ are predicted to contain two cytoplasmic protein subunits, including a bis-MGD cofactor-binding catalytic subunit that is smaller than that of the *Eggerthella* enzymes, and an iron–sulfur cluster-binding protein predicted to mediate electron transfer^4,15^ (Figure 1B). Both types of catechol dehydroxylases exhibit strict substrate specificity^4^ and their expression is highly induced by individual substrates.^4,8,12,16^ Bioinformatic analysis indicates hundreds of uncharacterized catechol dehydroxylases are encoded in the genomes of *Eggerthella*, *Gordonibacter*, and other Coriobacteriia genera.^4^

The study of catechol dehydroxylases has been challenging due to the well-recognized difficulty of establishing heterologous expression systems that generate active molybdopterin-dependent enzymes.^12,17^ Moreover, there was a lack of tools to genetically manipulate catechol dehydroxylase-encoding bacteria until we recently established a genetic toolkit for multiple Coriobacteriia species, including *Eggerthella lenta* and *Gordonibacter urolithinfaciens*.^16^ With these tools, we achieved the recombinant expression of *E. lenta* Dadh in a dopamine non-metabolizing *E. lenta* strain.^16^ However, we still lack an understanding of the general factors influencing catechol dehydroxylase activity and the diversity of substrates they process, both of which are required to fully harness their catalytic potential and elucidate their biological roles.

The availability of genetic tools now provides an unprecedented opportunity to enhance our understanding of catechol dehydroxylases. In particular, rapidly linking more members of this largely uncharacterized enzyme family^4^ to their substrates will help elucidate the relationship between protein sequences and activities, understand enzyme evolution, and accurately predict polyphenol metabolism by the gut microbiome, which is critical to deciphering the biological consequences of catechol dehydroxylation. Indeed, recent studies indicate this activity mediates anaerobic respiration in Coriobacteriia promoting their growth in vitro,^4,18,19^ perhaps explaining the evolution of numerous catechol dehydroxylases in response to the tremendous chemical diversity of dietary polyphenols.^1^

A particularly intriguing knowledge gap in polyphenol metabolism is the mechanism by which the gut microbiome transforms enantiomeric substrates. Many plant-derived polyphenols, including lignans and flavonoids, are chiral.^20^ In many cases, both enantiomers of the chiral polyphenols are produced either within a single plant species or by different species via enantioselective enzymes^20^ (Figure 1C). These polyphenols are metabolized in an enantioselective manner by different bacterial strains via transformations including dehydroxylation and oxidation (Figure 1C).^7,21^ While these studies did not reveal the enzymes responsible for this metabolism,^7,21^ they implied the possibility that gut bacterial enzymes have evolved to distinguish enantiomeric polyphenols.

Here, we leverage our genetic toolkit for Coriobacteriia to accelerate the functional characterization of catechol dehydroxylases. By establishing a heterologous expression system in the Coriobacteriia species *G. urolithinfaciens*, we rapidly characterize eight new catechol dehydroxylases from *Eggerthella* and *Gordonibacter* that participate in dietary flavonoid and lignan metabolism (Figure 1D). In particular, we discover multiple pairs of catechol dehydroxylases that have evolved to selectively metabolize individual catechol enantiomers and can be variably distributed in different Coriobacteriia. This is the first identification of enantiocomplementary polyphenol-metabolizing enzymes. Together, these molecular insights enhance our fundamental understanding of catechol dehydroxylase selectivity and evolution and will aid efforts to elucidate the health benefits of consuming polyphenol rich foods.

## Results

### Establishment of *G. urolithinfaciens* as a Heterologous Expression Host for Catechol Dehydroxylases Reveals Essential Roles of Accessory Genes

As highlighted above, lack of a suitable heterologous expression system^12,15^ hinders the study and engineering of catechol dehydroxylases. For example, previous attempted expression of over 20 Dadh constructs in multiple hosts failed to provide active enzyme, prompting the use of activity-guided native purification.^12^ However, native purification is time-consuming and precludes protein mutagenesis studies. Therefore, we sought to establish a heterologous expression system for *Eggerthella* catechol dehydroxylases.

**Figure 2:**
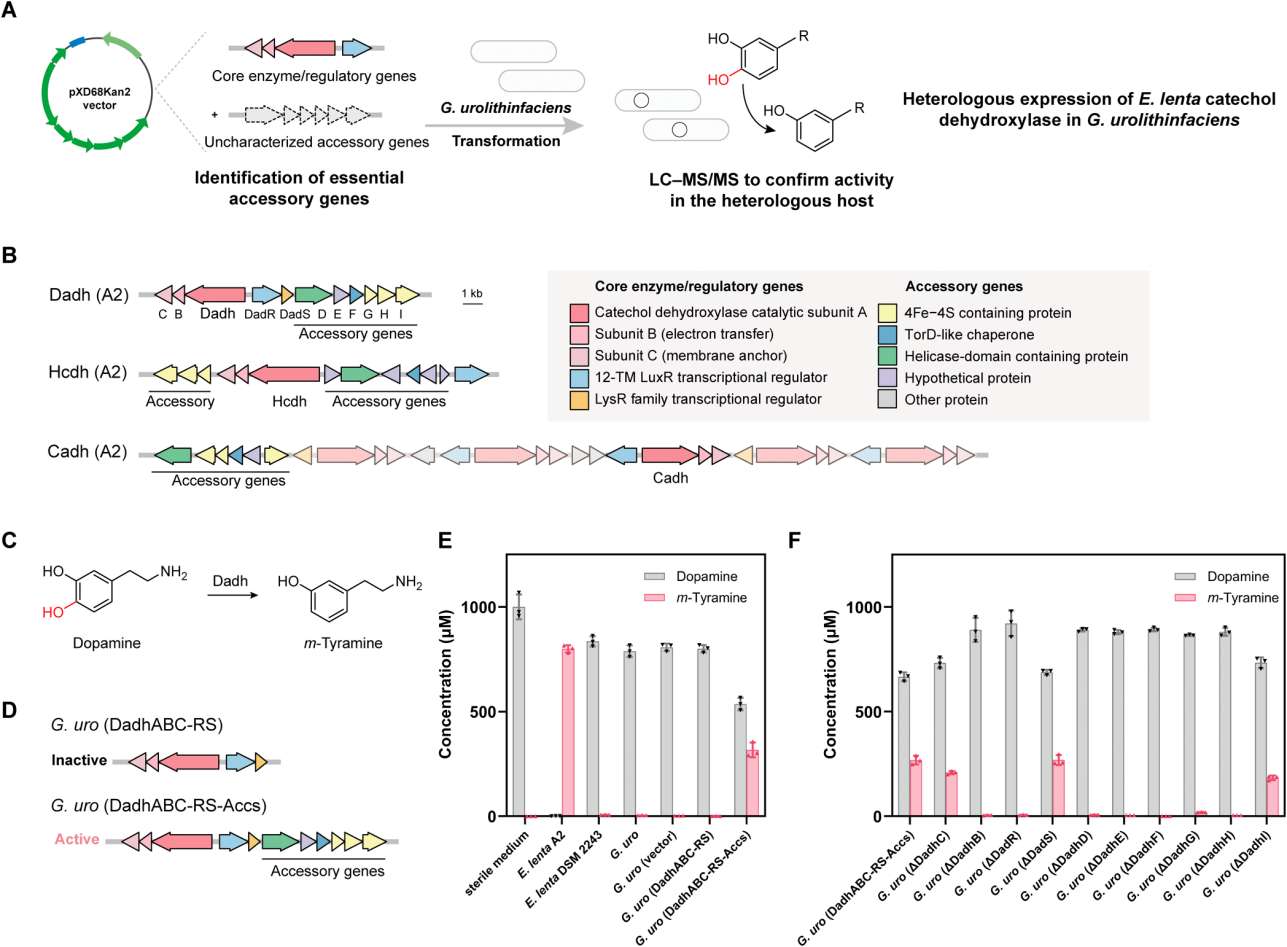
Development of a heterologous expression system to accelerate characterization of gut bacterial catechol dehydroxylases. (A) Schematic of establishing *G. uro* for heterologous expression of *E. lenta* catechol dehydroxylases. (B) Schematic of Dadh, Hcdh, Cadh gene clusters from *E. lenta* A2. (C) Dopamine dehydroxylation by Dadh. (D) Schematic of the construct design for the engineered *G. uro* strains *G. uro* (DadhABC-RS) and *G. uro* (DadhABC-RS-Accs). (E) LC–MS/MS data quantifying the production of *m*-tyramine after incubation of dopamine with corresponding *E. lenta* WT, *G. uro* WT and engineered *G. uro* strains. (F) LC–MS/MS data quantifying the production of *m*-tyramine after incubation of dopamine with *G. uro* strains engineered to test the importance of accessory genes in the *dadh* gene cluster. Data represented as mean ± SD with *n* = 3 biological replicates in (E) and (F).

*Gordonibacter urolithinfaciens* DSM 27213 (*G. uro*) was chosen as a potential expression host (Figure 2A) due to its close phylogenetic relationship to *E. lenta* and demonstrated genetic tractability.^16,22^ We first sought to express Dadh in *G. uro* (Figure 2B,C). We previously demonstrated the expression of active Dadh from *E. lenta* A2 in a dopamine non-metabolizing strain *E. lenta* DSM 2243 using a construct (DadhABC-RS) encoding the three Dadh subunits (DadhABC) and transcriptional regulators DadR/DadS^16^ (Figure 2D). However, this construct was not functional in *G. uro* (Figure 2E). We hypothesized that *G. uro* may lack genes essential for *dadh* expression and function. Previous RNA sequencing (RNA-seq) experiments^12^ revealed that multiple *E. lenta* genes near *dadh* are co-induced in the presence of dopamine, including a helicase domain-containing protein (DadhD), a hypothetical protein (DadhE), a TorD-like chaperone (DadhF), and Fe–S ferredoxins (DadhG, DadhH, DadhI) (Figure 2B). Though these gene products are uncharacterized, this co-regulation could indicate their involvement in the expression and/or function of *dadh*. To test this proposal, we introduced the whole *dadh* gene cluster from *E. lenta*, including the accessory genes, into *G. uro* on a plasmid (Figure 2D). We found that this *G. uro* (DadhABC-RS-Accs) strain can dehydroxylate dopamine (Figure 2E). Thus, we successfully heterologously expressed Dadh in *G. uro* using this strategy.

To further understand the contribution of individual uncharacterized accessory proteins to Dadh activity, we deleted individual genes from the plasmid encoding the *dadh* gene cluster and profiled the activity of each plasmid in *G. uro* (Figure 2B,F). Deleting *dadhC, dadS* or *dadhI* did not affect dopamine metabolism by *G. uro* (Figure 2F). In contrast, *dadhB*, *dadR,*(16) and *dadhD–H* were indispensable (Figure 2F). We still detected Dadh expression in most inactive strains except for *dadR* and *dadhD* deletion constructs (Figure S1A), suggesting most accessory proteins are involved in processes beyond protein expression, such as cofactor biosynthesis and/or insertion. To further investigate the potential functions of accessory proteins, we used AlphaFold3^23^ to predict their potential interactions with Dadh. We found that DadhF (chaperone) is predicted to contact the signal peptide and other parts of the Dadh catalytic subunit (Figure S1B). DadhE (hypothetical) is predicted to interact with the catalytic subunit (Figure S1B). The membrane-spanning ferredoxins DadhG and DadhH appear to interact with DadhB and DadhC, with their 4Fe-4S ferredoxin-type domains forming a potential electron transfer pathway with DadhB (Figure S1C). Similar accessory proteins are also encoded in the *E. lenta* hydrocaffeic acid dehydroxylase (Hcdh) and (+)-catechin dehydroxylase (Cadh) gene clusters, and AlphaFold3 predicts similar interactions between them and their associated dehydroxylases (Figure S2A,B), suggesting that these accessory proteins are also involved in enzyme maturation and electron transfer (Figure S2C).

The generality of this heterologous expression strategy was further illustrated by its application to two additional *E. lenta* catechol dehydroxylases. First, we introduced the *E. lenta* gene cluster encoding hydrocaffeic acid dehydroxylase (*El* Hcdh) (Figures 2B and S3A,B) into a previously generated *G. uro* (Δ*Gphcdh*) mutant^19^ in which the gene encoding the *Gordonibacter*-type hydrocaffeic acid dehydroxylase (*Gp* Hcdh) has been deleted. This expression strain exhibited hydrocaffeic acid dehydroxylation (Figure S3C).

Unlike Dadh and Hcdh, Cadh is encoded by a gene cluster containing five different catechol dehydroxylases (Figure 2B), four of which are uncharacterized. At one end of this gene cluster, we found genes encoding putative accessory proteins (Figure 2B). Unlike the accessory proteins encoded near *dadh* and *hcdh*, these genes are constitutively expressed in RNA-seq experiments.^4^ We hypothesized that they support the assembly of all catechol dehydroxylases in the gene cluster.

To test the roles of these accessory proteins in the expression of Cadh, we compared the activity of recombinant strains *G. uro* (Cadh), which harbors a plasmid encoding the three enzyme subunits CadhABC and regulator CadR, and *G. uro* (Cadh-Accs) which additionally encodes putative accessory genes (Figure S3D,E). As C–O cleavage of catechin precedes dehydroxylation and *G. uro* lacks this C–O cleavage activity (Figure S3D), we also identified the *E. lenta* enzyme that cleaves the C–O bond of (+)-catechin. Previous RNA-seq results^4^ revealed a flavin-dependent enzyme (Elen_0616) is highly induced in the presence of (+)-catechin, suggesting it might perform this reaction. We therefore named this enzyme Cber for catechin benzyl ether reductase, and cloned a plasmid encoding Cber, its cognate promoter and transcriptional regulator (Figure S3E). Indeed, a *G. uro* strain harboring this plasmid *G. uro* (Cber) cleaved (+)-catechin (Figure S3F) into (*R*)-**1**. Co-incubation of *G. uro* (Cber) with *G. uro* (Cadh-Accs) but not *G. uro* (Cadh) provided dehydroxylated product (*R*)-**6** (Figure S3F), demonstrating the importance of the shared accessory genes for Cadh activity. Notably, the accessory genes from the Cadh gene cluster could not support the activity of Dadh (Figure S3G–I), suggesting these accessory genes are specific to the enzymes encoded in this gene cluster. In addition, these heterologous expression constructs were not active in *E. coli* (Figure S4A–C). Taken together, this work establishes *G. uro* as a heterologous expression host for previously characterized *E. lenta* catechol dehydroxylases and elucidates the requirement for accessory genes to produce active enzymes.

### *E. lenta* Encodes Enantiocomplementary Catechin Dehydroxylases

**Figure 3:**
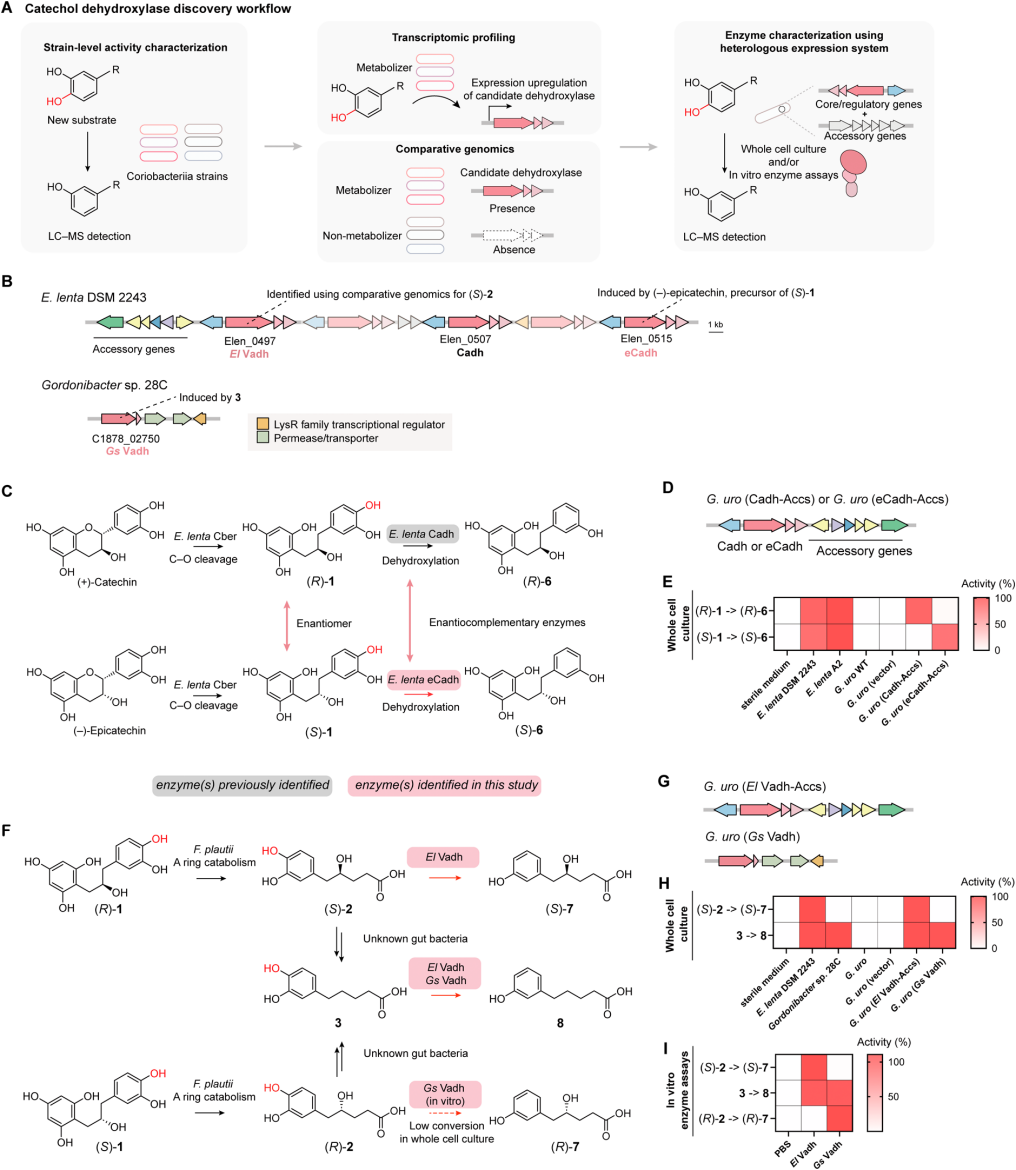
Heterologous expression enables rapid identification and characterization of enantioselective catechol dehydroxylases involved in gut bacterial catechin metabolism. (A) A workflow for catechol dehydroxylase discovery and characterization. (B) Schematic of the gene cluster in *E. lenta* DSM 2243 encoding the newly identified catechol dehydroxylases eCadh and *El* Vadh, the previously identified catechol dehydroxylase Cadh, and accessory genes as well as and the gene cluster in *Gordonibacter* sp. 28C encoding the newly identified catechol dehydroxylase *Gs* Vadh. (C) *E. lenta* metabolism of (−)-epicatechin and (+)-catechin. (D) The construct design for the engineered *G. uro* strains *G. uro* (Cadh-Accs) and *G. uro* (eCadh-Accs) to characterize activity of Cadh and eCadh. (E) LC–MS/MS data quantifying the production of dehydroxylated metabolites (*R*)-**6** and (*S*)-**6** after incubation with *G. uro* strains expressing Cadh or eCadh to test substrate specificity in whole cell culture. *E. lenta* DSM 2243 and A2 are positive controls and *G. uro* WT and *G. uro* (vector) are negative controls. (F) Gut bacterial metabolism of (*R*)-**1** (derived from (+)-catechin) to (*S*)-**7** and **8**, and metabolism of (*S*)-**1** (derived from (−)-epicatechin) to (*R*)-**7** and **8**. (G) The construct design for the engineered *G. uro* strains *G. uro* (*El* Vadh-Accs) and *G. uro* (*Gs* Vadh) for heterologous expression. (H) LC–MS/MS data quantifying the production of dehydroxylated metabolites (*S*)-**7** and **8** after incubation with *G. uro* strains expressing *El* Vadh or *Gs* Vadh to characterize enzyme specificity in whole-cell culture. *E. lenta* DSM 2243 and *Gordonibacter* sp. 28C are positive controls and *G. uro* WT and *G. uro* (vector) are negative controls. (I) LC–MS/MS data quantifying the production of dehydroxylated metabolites (*S*)-**7**, (*R*)-**7** and **8** after incubation with purified *El* Vadh or *Gs* Vadh to characterize enzyme specificity in vitro. PBS is a negative control. Data represented are means normalized to the highest intensity for each metabolite with *n* = 3 biological replicates in E, H, and I.

We next sought to utilize the *G. uro* heterologous expression system to characterize catechol dehydroxylases of unknown function. We envisioned testing the activity of individual Coriobacteriia strains toward catechol substrates, combining transcriptional profiling and comparative genomics approaches to identify genes encoding candidate dehydroxylases in active strains, and then characterizing enzyme activity using the *G. uro* heterologous expression system (Figure 3A).

To prioritize catechol substrates to test, we considered the specificities and genomic contexts of characterized catechol dehydroxylases. Besides Dadh, Hcdh, and Cadh,^4,12^*E. lenta* DSM 2243 encodes four uncharacterized catechol dehydroxylases within the same gene cluster as *cadh* (Figure 3B). Considering their co-localization with *cadh* and the shared accessory genes, we hypothesized that these uncharacterized enzymes might dehydroxylate substrates structurally or metabolically related to the substrate of Cadh, (*R*)-**1**.

One such polyphenol is (−)-epicatechin, a naturally occurring diastereomer of (+)-catechin that is abundant in cacao and tea^24^ and provides anti-inflammatory and antioxidant benefits.^25^ Both (+)-catechin and (−)-epicatechin undergo C-ring benzyl ether cleavage and B-ring dehydroxylation by the gut microbiome (Figure 3C).^10,26,27^ However, it remains unclear if Cadh or a different enzyme mediates dehydroxylation of (*S*)-**1**, the C-ring cleaved product of (−)-epicatechin. Similar to previous reports,^4^ we confirmed using LC–MS/MS that some (+)-catechin metabolizing strains cannot metabolize (−)-epicatechin (Figure S6A–D), suggesting an enzyme other than Cadh dehydroxylates (*S*)-**1**.

To identify the responsible enzyme, we incubated *E. lenta* DSM 2243, which metabolizes both (+)-catechin and (−)-epicatechin, with (−)-epicatechin and used reverse transcription-quantitative PCR (RT-qPCR) to measure the expression of *cadh* and the four genomically co-localized, uncharacterized catechol dehydroxylases. Elen_0515, one of the uncharacterized catechol dehydroxylases, was highly induced by (−)-epicatechin (Figures 3B and S6E) and named eCadh for (−)-epicatechin dehydroxylase. Co-incubation of *G. uro* (Cber), which cleaves (−)-epicatechin, and *G. uro* (eCadh-Accs), which encodes a regulator, three subunits of eCadh and accessory genes, with (−)-epicatechin produced the dehydroxylated metabolite (*S*)-**6** (Figures 3D and S6F), confirming eCadh dehydroxylates (*S*)-**1**. This substrate is the enantiomer of (*R*)-**1**, the substrate of Cadh.

We then examined enzyme specificity by testing the activity of *G. uro* strains heterologously expressing Cadh or eCadh toward (*R*)-**1** and (*S*)-**1** (Figure S5A). The heterologously expressed Cadh only dehydroxylated (*R*)-**1** (Figures 3D,E and S6G,H). While eCadh efficiently dehydroxylated (*S*)-**1**, it displayed only weak activity when incubated with (+)-catechin CFS (∼3% of the Cadh-encoding strain) (Figures 3E and S6G,H). The striking difference in the reactivity of Cadh and eCadh toward (*R*)-**1** and (*S*)-**1** highlights that catechol dehydroxylases have evolved to accept individual enantiomeric substrates.

### *El* Vadh and *Gs* Vadh Dehydroxylate Phenolic Acid Intermediates from Gut Bacterial Metabolism of Catechins

We hypothesized that the other uncharacterized enzymes in the Cadh gene cluster may also participate in catechin metabolism. After initial C-ring benzyl ether cleavage, regardless of B-ring dehydroxylation, intestinal bacteria such as *Flavonifractor plautii* can break down the A ring of catechins, generating 5-(3′,4′-dihydroxyphenyl)-4-hydroxyvaleric acid as a product (DHPHVA, **2**)^26^ (Figure 3F). Previous studies suggest certain *E. lenta* and *Adlercreutzia equolifaciens* strains can dehydroxylate 5-(3′,4′,5′-trihydroxyphenyl)-4-hydroxyvaleric acid (THPHVA), a structural analog of **2** derived from (−)-epigallocatechin.^9,11^ However, the enzyme(s) responsible are unclear.

To fully decipher (epi)catechin metabolism, we sought to discover the enzymes that dehydroxylate these phenolic acid intermediates. To access (*S*)-**2** and (*R*)-**2**, we incubated *F. plautii* DSM 4000 and *E. lenta* AB8n2 with either (+)-catechin or (−)-epicatechin (Figure S5B). After incubation, we detected production of (*S*)-**2** from (+)-catechin and (*R*)-**2** from (−)-epicatechin. We used the CFS diluted with fresh medium to test (*S*)-**2** and (*R*)-**2** dehydroxylation by different Coriobacteriia strains. (*S*)-**2** underwent complete dehydroxylation by a subset of *Eggerthella* strains, whereas (*R*)-**2** was inefficiently dehydroxylated by some of these *Eggerthella* strains and *Gordonibacter* sp. 28C (4–22% signal compared to (*S*)-**2**) (Figure S7A–D). To identify the enzyme(s) responsible for these activities, we compared the catechol dehydroxylases encoded by *E. lenta* DSM 2243, which metabolizes (*S*)-**2**, with those encoded by *E. lenta* A2, which lacks this activity. We found that one catechol dehydroxylase (Elen_0497, *El* Vadh for *E. lenta* 5-(3′,4′-dihydroxyphenyl)-4-hydroxyvaleric acid dehydroxylase) was unique to *E. lenta* DSM 2243 (Figures 3B and S8A). Heterologous expression of *El* Vadh in *G. uro* confirmed its activity for (*S*)-**2** dehydroxylation (Figure 3G,H).

Interestingly, *Gordonibacter* sp. 28C, which showed low activity toward (*R*)-**2** and no activity toward (*S*)-**2** (Figure S7A–D), does not encode *El* Vadh, suggesting the involvement of an uncharacterized enzyme in (*R*)-**2** metabolism. Moreover, the low activities of *Gordonibacter* sp. 28C toward both substrates suggested neither compound is the native substrate of the enzyme. Considering the structure of (*S*)-**2**/(*R*)-**2**, we examined 5-(3′,4′-dihydroxyphenyl)valeric acid (DHPVA, **3**) which lacks a secondary hydroxyl substituent (Figure 3F) and is thought to be generated from **2** during the metabolism of catechins by uncharacterized gut bacteria (Figure 3F).^10^ We found that *Gordonibacter* sp. 28C and the (*S*)-**2**-metabolizing *Eggerthella* strains completely dehydroxylated **3** (Figure S7E,F).

To identify the *Gordonibacter* sp. 28C enzyme that dehydroxylates **3**, we incubated this strain with **3** and used RT-qPCR to identify a highly induced catechol dehydroxylase gene C1878_02750 (Figure S8B). Comparing the genomic context of this gene in *Gordonibacter* sp. 28C and other *Gordonibacter* strains (Figure S8C) revealed a gene cluster that encodes two catechol dehydroxylase subunits, two transporters, and a LysR-type transcriptional regulator (Figure 3B). We named the encoded catechol dehydroxylase *Gs* Vadh for *Gordonibacter* sp. 5-(3′,4′-dihydroxyphenyl)valeric acid dehydroxylase. *G. uro* (*Gs* Vadh) heterologously expressing *Gs* Vadh showed complete metabolism of **3**, minimal activity toward (*R*)-**2**, and no activity toward (*S*)-**2**, consistent with the activity of *Gordonibacter* sp. 28C (Figures 3H and S8D–F). In comparison, the recombinant *G. uro* (*El* Vadh-Accs) strain expressing *El* Vadh showed nearly complete metabolism of (*S*)-**2**, complete metabolism of **3**, but no activity toward (*R*)-**2** (Figures 3H and S8D–F), aligning with the activity of *E. lenta* DSM 2243.

To understand if the observed substrate preference of each enzyme in culture is solely due to enzyme selectivity or impacted by other factors such as transcriptional regulation or substrate transport, we adapted our previously developed cumate-inducible expression system^16^ to express His-tagged *El* Vadh and *Gs* Vadh in *G. uro* in a substrate-independent manner (Figure S8G) and purified both enzymes to perform in vitro assays (Figure S8H). The in vitro substrate selectivity of *El* Vadh is consistent with that of *G. uro* (*El* Vadh-Accs) whole-cell culture (Figures 3I and S8I–K). However, while *G. uro* (*Gs* Vadh) whole-cell culture only efficiently dehydroxylates **3**, the *Gs* Vadh enzyme fully dehydroxylated both (*R*)-**2** and **3** in vitro (Figures 3I and S8I–K), which suggests that the low activity of *Gs* Vadh-encoding strains toward (*R*)-**2** might result from limited enzyme induction by (*R*)-**2** or (*R*)-**2** transport in the native system, rather than enzyme selectivity.

Overall, these results identified two enzymes from *Eggerthella* and *Gordonibacter*, respectively, that process **3**, with *El* Vadh additionally metabolizing (*S*)-**2** and *Gs* Vadh additionally dehydroxylating (*R*)-**2** in vitro. Together with characterization of eCadh and Cadh, these results reveal the complexity of interspecies metabolism of catechins in the human gut (Figure S9).

### *Eggerthella* and *Gordonibacter* Encode Enantiocomplementary Dehydroxylases That Produce Enterodiols

**Figure 4:**
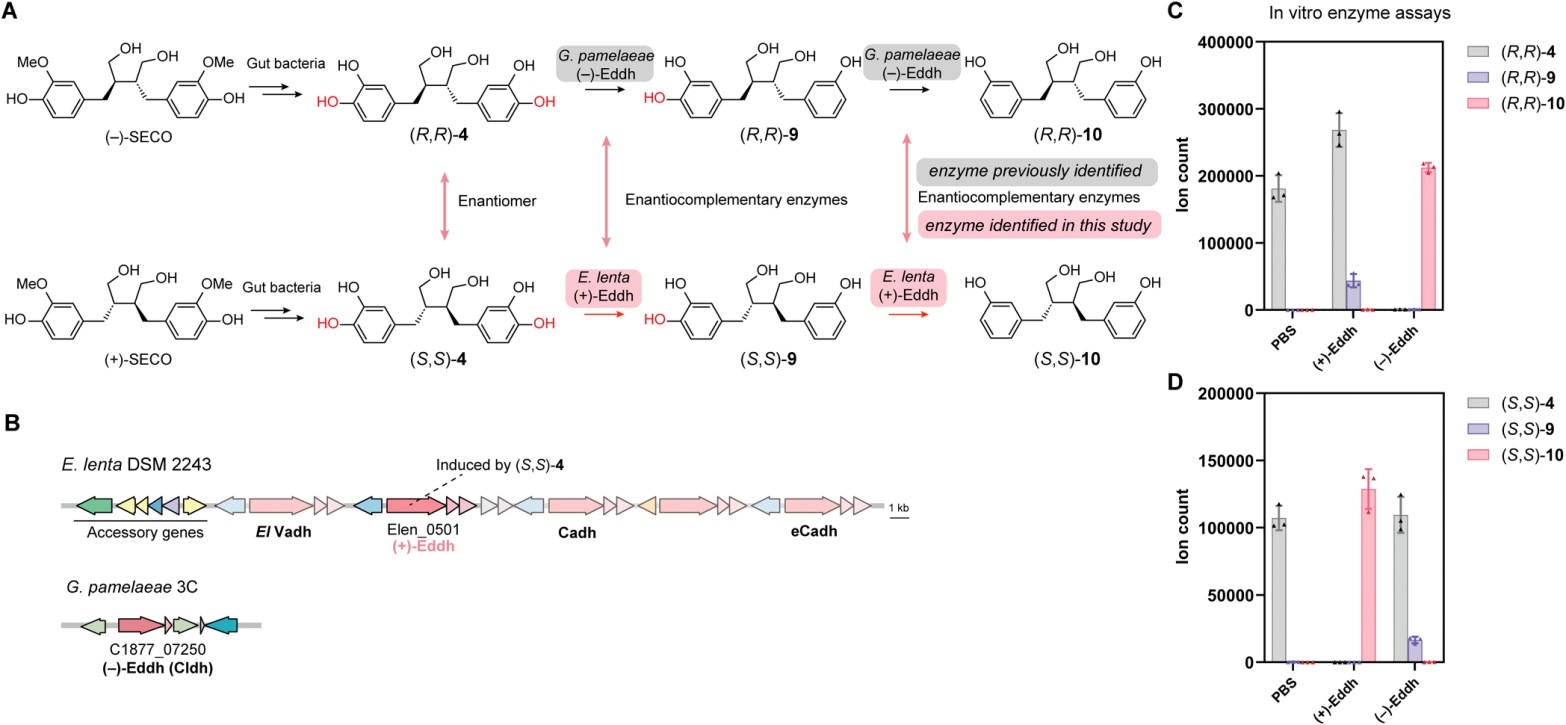
*Eggerthella* (+)-Eddh and *Gordonibacter* (−)-Eddh are enantiocomplementary dehydroxylases that produce enterodiols. (A) Gut bacterial metabolism of (−)-SECO and (+)-SECO. (B) Schematic of the gene cluster in *E. lenta* DSM 2243 encoding the newly identified (+)-Eddh enzyme with co-clustered catechol dehydroxylases and accessory genes, as well as the *G. pamelaeae* 3C gene cluster encoding the previously identified (−)-Eddh enzyme. (C, D) LC–MS/MS data quantifying the production of dehydroxylated metabolites after incubation of (C) (*R*,*R*)-**4** or (D) (*S*,*S*)-**4** with purified (+)-Eddh, (−)-Eddh or PBS negative control. Data represented as mean ± SD with *n* = 3 biological replicates in C and D.

The discovery that Cadh and eCadh dehydroxylate enantiomeric intermediates from catechin metabolism adds to a growing list of naturally occurring enantiocomplementary enzymes.^28^ We next sought to discover other enantiocomplementary catechol dehydroxylases by focusing on metabolism of plant lignans, a family of polyphenols abundant in flaxseeds, whole grains and fruits.^20^ Lignans are transformed via cooperative gut bacterial metabolism to generate enterolignan products which lack *para*-hydroxyl groups.^7^ Enterolignans can exist in enterodiol (END) and enterolactone (ENL) forms, with both enantiomers generated by the gut microbiome.^7^ Intriguingly, the catechol precursors of each enterolignan isomer are dehydroxylated by different Coriobacteriia species in an enantioselective manner.^7^ In gut bacterial metabolism of (+)-pinoresinol, (−)-dihydroxyenterodiol [(−)-DHEND, (*R*,*R*)-**4**] is produced, and transcriptomic analyses identified an enzyme (Cldh) present in multiple *Gordonibacter* strains that potentially dehydroxylates (*R*,*R*)-**4** to (−)-END [(*R*,*R*)-**10**] (Figure 4A),^8^ although its activity has not been biochemically confirmed. The enantiomer of (*R*,*R*)-**4**, (+)-dihydroxyenterodiol [(+)-DHEND, (*S*,*S*)-**4**] is generated during bacterial metabolism of (+)-secoisolariciresinol [(+)-SECO] diglucoside (Figure 4A), a lignan enriched in flaxseed, to enterolignans (+)-END [(*S*,*S*)-**10**] and (+)-ENL [(*S*,*S*)-**13**].^29−31^ These studies highlight the crucial roles of gut microbes in the bioactivation of plant lignans. However, the enzyme responsible for the dehydroxylation of (*S*,*S*)-**4** remains unknown (Figure 4A).

To identify the (*S*,*S*)-**4** dehydroxylase, we first obtained (*S*,*S*)-**4** from *B. producta*-mediated demethylation of (+)-SECO.^30^ The resulting CFS was diluted and tested for dehydroxylation by selected Coriobacteriia strains (Figure S5C). *E. lenta* strains showed differing levels of activity toward (*S*,*S*)-**4**, while none of the *Gordonibacter* strains showed activity (Figure S10A,B). RT-qPCR revealed (*S*,*S*)-**4** induced the transcription of uncharacterized dehydroxylase Elen_0501 in *E. lenta* DSM 2243, which we named (+)-Eddh for (+)-dihydroxyenterodiol dehydroxylase (Figures 4B and S10C). The activity of (+)-Eddh toward (*S*,*S*)-**4** was confirmed by heterologous expression in *G. uro* (Figure S10D,E). We also confirmed the metabolism of (*R*,*R*)-**4** by *Gordonibacter* but not *Eggerthella* strains as previously reported^8^ (Figure S11A,B), and demonstrated the activity of the *Gordonibacter* Cldh enzyme (renamed (−)-Eddh for (−)-dihydroxyenterodiol dehydroxylase) toward (*R*,*R*)-**4** by heterologous expression in *E. lenta* (Figures 4B and S11C,D). To determine the enantioselectivity of (+)-Eddh and (−)-Eddh, we purified individual enzymes from cumate-induced expression in *G. uro* (Figure S11E) and assayed their in vitro activities toward (*S*,*S*)-**4** and (*R*,*R*)-**4**. These two enzymes exhibited complementary enantioselectivity, fully dehydroxylating their preferred substrate enantiomer and only showing low activity toward the opposite enantiomer (Figure 4C,D). Notably, as these enantiocomplementary enzymes are from different Coriobacteriia genera, they are predicted to have significantly different structural features and subunit architectures.

### *Eggerthella* and *Gordonibacter* Encode Enantiocomplementary and Site-Selective Dehydroxylases That Produce Enterolactones

**Figure 5:**
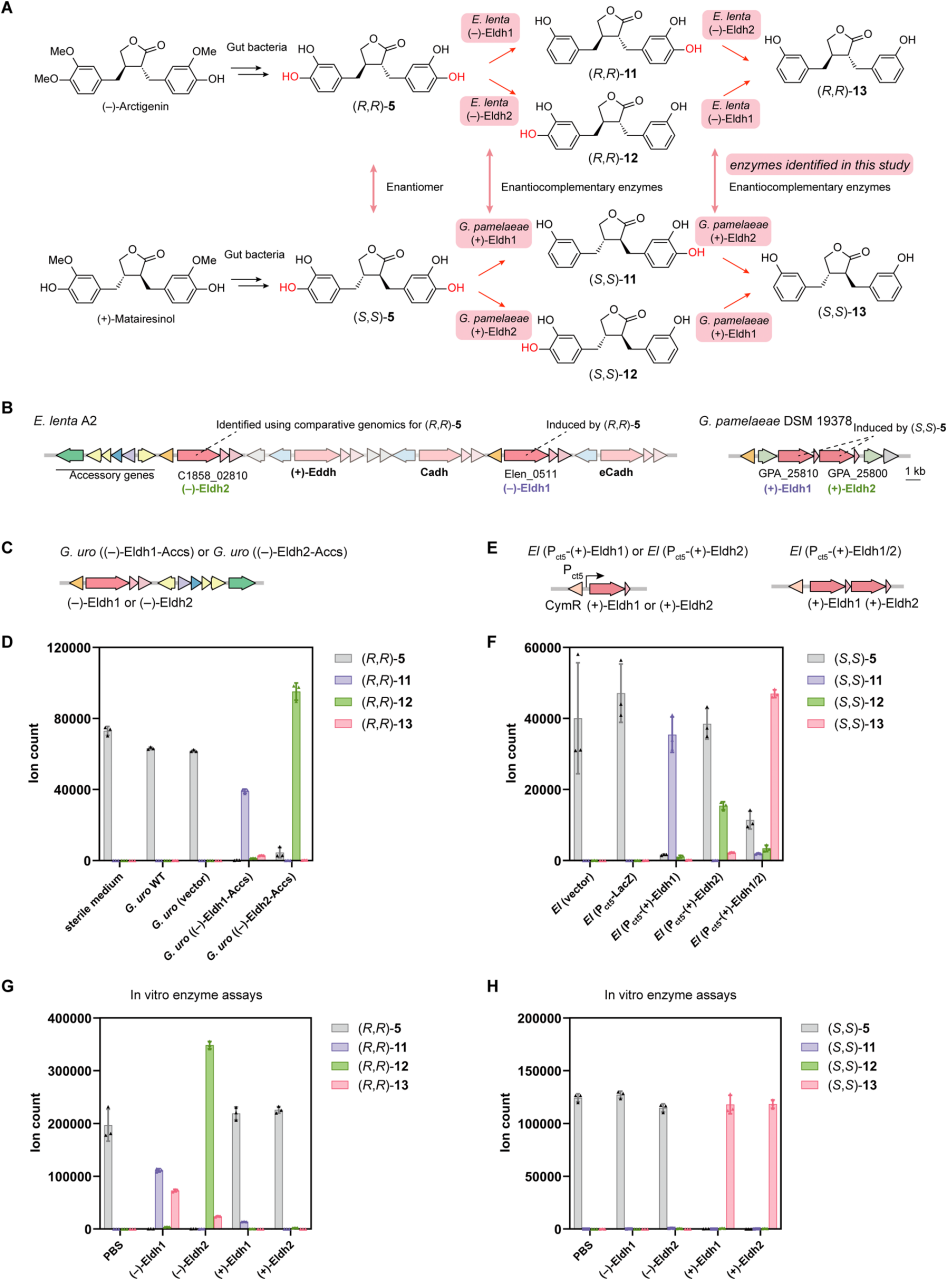
*Eggerthella* (−)-Eldh1 and (−)-Eldh2 and *Gordonibacter* (+)-Eldh1 and (+)-Eldh2 are site-selective, enantiocomplementary dehydroxylases that produce enterolactones. (A) Gut bacterial metabolism of (−)-arctigenin and (+)-matairesinol. (B) Schematic of the *E. lenta* A2 gene cluster encoding (−)-Eldh1 and (−)-Eldh2, co-clustered catechol dehydroxylases and accessory genes, as well as the *G. pamelaeae* DSM 19378 gene cluster encoding (+)-Eldh1 and (+)-Eldh2. (C) Construct design for the engineered *G. uro* strains *G. uro* ((−)-Eldh1-Accs) and *G. uro* ((−)-Eldh2-Accs). (D) LC–MS/MS data quantifying the production of dehydroxylated metabolites (*R*,*R*)-**11**, (*R*,*R*)-**12** and (*R*,*R*)-**13** after incubation of (*R*,*R*)-**5** with corresponding *G. uro* WT and engineered *G. uro* strains. (E) Construct design for the engineered *E. lenta* DSM 2243 strains *El* (P_ct5_-(+)-Eldh1), *El* (P_ct5_-(+)-Eldh2) and *El* (P_ct5_-(+)-Eldh1/2). (F) LC–MS/MS to quantify the production of dehydroxylated metabolites (*S*,*S*)-**11**, (*S*,*S*)-**12** and (*S*,*S*)-**13** after incubation of (*S*,*S*)-**5** with corresponding engineered *E. lenta* strains. (G, H) LC–MS/MS data quantifying the dehydroxylated metabolites after incubation of (G) (*R*,*R*)-**5** or (H) (*S*,*S*)-**5** with purified (−)-Eldh1, (−)-Eldh2, (+)-Eldh1, (+)-Eldh2 or PBS negative control. Data represented as mean ± SD with *n* = 3 biological replicates in (D) and (F–H).

The discovery of *Eggerthella* (+)-Eddh and *Gordonibacter* (−)-Eddh encouraged us to examine additional uncharacterized dehydroxylation events in lignan metabolism. Besides (*R*,*R*)-**4** and (*S*,*S*)-**4**, the corresponding oxidized and lactonized products (−)-dihydroxyenterolactone [(−)-DHENL, (*R*,*R*)-**5**] and (+)-dihydroxyenterolactone [(+)-DHENL, (*S*,*S*)-**5**] are generated by gut bacteria (Figure S12A),^8,21,29,32^ and can be dehydroxylated by Coriobacteriia.^7^ They can also be directly derived from the lignans (−)-matairesinol/(−)-arctigenin and (+)-matairesinol, respectively, via bacterial demethylation (Figure S12A).^33^ We thus sought to characterize the enzymes(s) involved in the dehydroxylation of (*R*,*R*)-**5** and (*S*,*S*)-**5** (Figure 5A).

To identify Coriobacteriia strains that dehydroxylate (*R*,*R*)-**5**, we used chemical approaches to demethylate (−)-arctigenin to (*R*,*R*)-**5**^8^ (Figure S5D) and incubated a panel of Coriobacteriia strains with this compound. While multiple *E. lenta* strains dehydroxylated (*R*,*R*)-**5** with different accumulating products (Figure S12B,C), none of the *Gordonibacter* strains showed activity. *E. lenta* A2 and MR1n12 showed complete conversion to (*R*,*R*)-**13**, while other *E. lenta* strains generate partially dehydroxylated compounds as the major product. Interestingly, the partially dehydroxylated product (*R*,*R*)-**11**, generated by *E. lenta* DSM 2243, had a dominant MS daughter ion with *m*/*z* of 191.07, different from (*R*,*R*)-**12** produced by some other strains, which had a dominant daughter ion with *m*/*z* of 269.11 (Figure S13A,B). Comparing the fragmentation patterns of (*R*,*R*)-**11** and (*R*,*R*)-**12** (Figure S13C,D) to those reported previously^34,35^ allowed us to assign their dehydroxylation sites. Notably, even though (*R*,*R*)-**11**/(*R*,*R*)-**12** are the major products of metabolism by these strains, we still detected the fully dehydroxylated product (*R*,*R*)-**13** in lower amounts. We reasoned that the observed products could be generated by two different enzymes variably distributed in *E. lenta* strains, each performing site-selective dehydroxylation of one *p*–OH group of (*R*,*R*)-**5**, while processing the other *p*–OH at a slower rate.

To identify the enzyme(s) responsible for the dehydroxylation of (*R*,*R*)-**5**, we incubated *E. lenta* DSM 2243, which preferably produces (*R*,*R*)-**11**, with (*R*,*R*)-**5**. RT-qPCR showed that Elen_0511 [named (−)-Eldh1 for (−)-dihydroxyenterolactone dehydroxylase 1] was significantly induced (Figures 5B and S12D), suggesting it encodes a catechol dehydroxylase that produces (*R*,*R*)-**11**. To identify the enzyme(s) responsible for the second dehydroxylation event, we compared the catechol dehydroxylases of *E. lenta* DSM 2243, which mainly produces (*R*,*R*)-**11**, and *E. lenta* A2, which produces (*R*,*R*)-**13**. We found a catechol dehydroxylase unique to *E. lenta* A2, encoded by C1858_02810 [named (−)-Eldh2 for (−)-dihydroxyenterolactone dehydroxylase 2] (Figures 5B and S12E), suggesting it may dehydroxylate the other *p*–OH group on (*R*,*R*)-**5**. We then heterologously expressed these two enzymes in *G. uro* (Figure 5C). Consistent with our hypothesis, *G. uro* ((−)-Eldh1-Accs) primarily produced (*R*,*R*)-**11**, and *G. uro* ((−)-Eldh2-Accs) produced (*R*,*R*)-**12** as a major product (Figure 5D). In summary, we identified two different enzymes from *E. lenta* that selectively dehydroxylate different *p*–OH groups on (*R*,*R*)-**5**. The discovery of these enzymes also represents the complete assignment of all catechol dehydroxylases in *E. lenta* strains DSM 2243 and A2.

Next, we investigated the dehydroxylation of (*S*,*S*)-**5** to identify enzymes enantiocomplementary to (−)-Eldh1 and (−)-Eldh2. To obtain (*S*,*S*)-**5**, (+)-matairesinol was incubated with *B. producta* (Figure S5D) and extracted using ethyl acetate. We then tested different Coriobacteriia strains for their activity toward (*S*,*S*)-**5**. While *E. lenta* strains encoding (−)-Eldh1 and/or (−)-Eldh2 did not dehydroxylate (*S*,*S*)-**5**, all four *Gordonibacter* strains tested dehydroxylated this substrate (Figure S14A,B). Screening for the induction of uncharacterized catechol dehydroxylases by (*S*,*S*)-**5** in *G. pamelaeae* DSM 19378 using RT-qPCR revealed that two genomically colocalized genes encoding catechol dehydroxylases [GPA_25810, named (+)-Eldh1 for (+)-dihydroxyenterolactone dehydroxylase 1 and GPA_25800, named (+)-Eldh2 for (+)-dihydroxyenterolactone dehydroxylase 2] were highly induced (Figures 5B and S14C). When heterologously expressed in *E. lenta*, (+)-Eldh1 transformed (*S*,*S*)-**5** completely to (*S*,*S*)-**11** while (+)-Eldh2 mainly produced (*S*,*S*)-**12** (Figures 5F and S14D,E). Induction of the strain encoding both enzymes produced (*S*,*S*)-**13** as a major product (Figure 5F). The reactivity of (+)-Eldh1 and (+)-Eldh2 toward (*S*,*S*)-**5** parallels that of (−)-Eldh1 and (−)-Eldh2 toward (*R*,*R*)-**5**.

To verify the enantioselectivity of the Eldh enzymes, we purified individual enzymes (Figure S14F) and profiled their activity toward (*R*,*R*)-**5** and (*S*,*S*)-**5**. We confirmed each enzyme showed high enantioselectivity, completely transforming their preferred substrate and displaying low to no conversion of the unfavored enantiomer (Figure 5G,H). The enantiocomplementary relationship between *Gordonibacter* (+)-Eldh1 and (+)-Eldh2 and *Eggerthella* (−)-Eldh1 and (−)-Eldh2 again highlights the prominent evolutionary trajectory of catechol dehydroxylases to process polyphenols of different chirality.

Finally, the co-localization of the genes encoding all the newly characterized *E. lenta* catechol dehydroxylases raises interesting questions about their specificity. We profiled the substrate specificity of these enzymes by measuring the activity of *G. uro* whole-cell cultures expressing different catechol dehydroxylases toward each newly identified *E. lenta* enzyme substrate. All the enzymes showed high activity toward their characterized substrates and showed little-to-no cross-reactivity toward substrates of other enzymes (Figure S15A), highlighting the high substrate specificity of these enzymes, a feature that has been typical of all catechol dehydroxylases characterized to date.

### Phylogenetic Analysis of Catechin- and Lignan-Metabolizing Catechol Dehydroxylases

**Figure 6:**
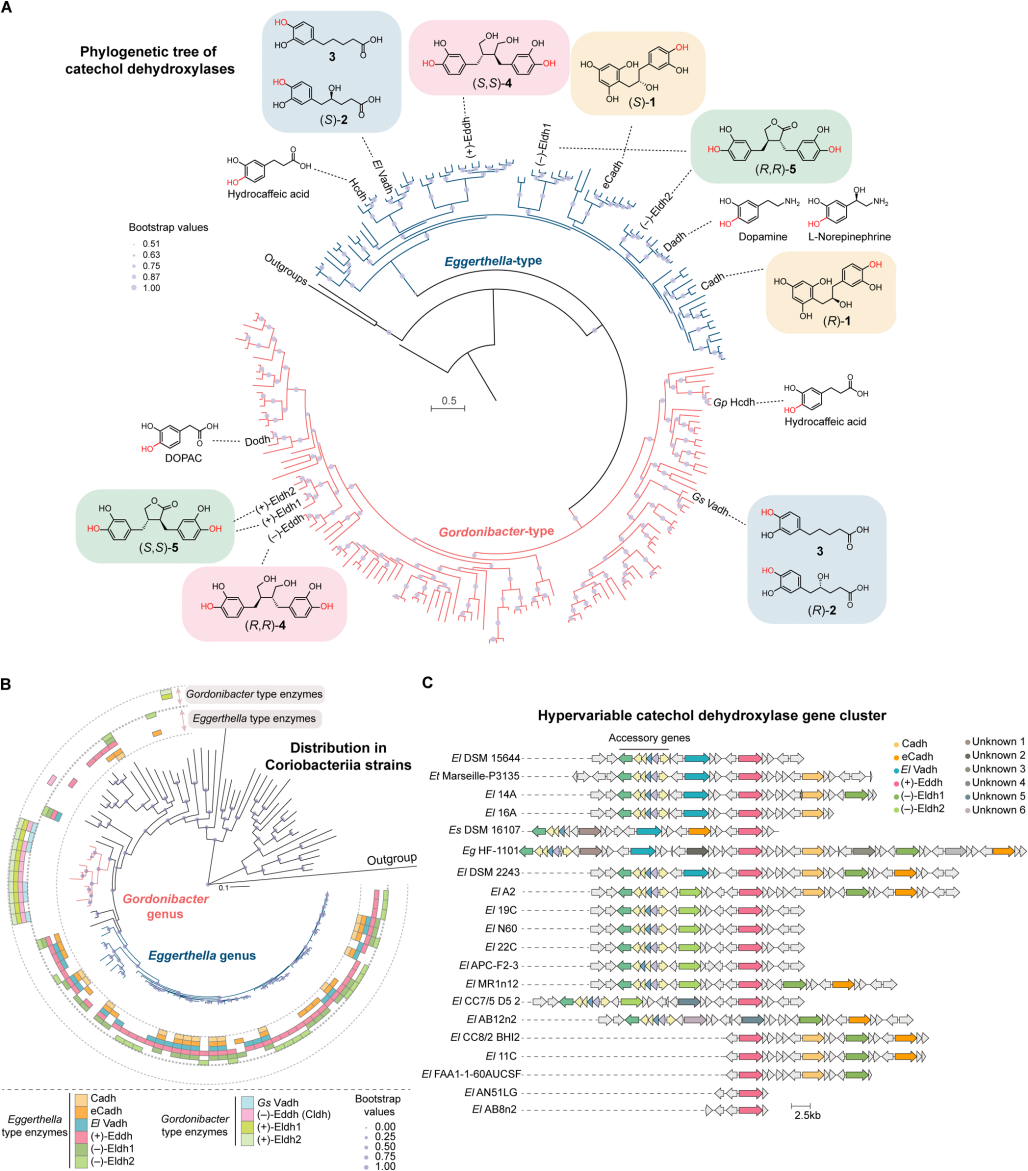
Analyses of gut bacterial dehydroxylase sequences reveal varied evolutionary histories and distribution across gut Coriobacteriia. (A) A maximum-likelihood phylogenetic tree of the catalytic subunits of characterized and predicted Coriobacteriia catechol dehydroxylases. Sequences are grouped by 95% amino acid identity. The characterized catechol dehydroxylases and their substrates are highlighted in the tree. The same color shading of the chemical structures indicates substrate enantiomers or the same substrate. Other biochemically characterized members of the DMSO reductase superfamily (DMSO reductase DorA, ethylbenzene dehydrogenase EbdA, and pyrogallol hydroxytransferase AthL) were used as outgroups. (B) Distribution of catechol dehydroxylases in gut Coriobacteriia genomes. Phylogenetic analysis of Coriobacteriia genomes was performed using PhyloPhlAn v3.0.^36^ Distribution of the catechol dehydroxylases of interest in different Coriobacteriia genomes was analyzed by BLAST searches against a local database consisting of the 113 Coriobacteriia genomes. BLAST hits with coverage of over 85% and amino acid identity of over 80% for *Gs* Vadh or 75% for other enzyme queries were considered enzyme homologues. (C) Variable content of a hypervariable catechol dehydroxylase encoding gene cluster in *Eggerthella* strains. Accessory genes and individual catechol dehydroxylase-encoding genes are colored.

The discovery of enantiocomplementary and site-selective catechol dehydroxylase enzymes in gut bacterial catechin and lignan metabolism (Figure S15B) raises interesting questions regarding their evolutionary trajectories. To explore this further, we performed a maximum likelihood phylogenetic analysis of the catalytic subunits of all characterized and uncharacterized Coriobacteriia catechol dehydroxylases (Figure 6A). As expected, *Eggerthella-* and *Gordonibacter*-type catechol dehydroxylases form two separate clades. Interestingly, the inferred tree revealed multiple instances of phylogenetically related enzymes sharing structurally similar substrates. For example, we found that the *Gordonibacter* enzymes (+)-Eldh1 and (+)-Eldh2 share particularly high amino acid identity (74%) and are closest to each other on the phylogenetic tree (Figure 6A), potentially suggesting a common ancestral sequence for these site-selective enzymes that accept the same substrate. We also found that (−)-Eddh is the closest neighbor to (+)-Eldh1 and (+)-Eldh2 on the phylogenetic tree (Figure 6A), suggesting a close relationship among these *Gordonibacter* lignan-metabolizing enzymes. In addition, *El* Vadh, which dehydroxylates (*S*)-**2** and **3**, and *El* Hcdh, which dehydroxylates hydrocaffeic acid, are located close to one another on the tree (Figure 6A). (*S*)-**2**, **3** and hydrocaffeic acid belong to the larger family of phenolic acids and are structurally similar. Notably, *Gordonibacter*-type enzymes that metabolize related substrates, *Gs* Vadh which dehydroxylates (*R*)-**2** and **3** and *Gp* Hcdh which dehydroxylates hydrocaffeic acid, are also close to one another within the *Gordonibacter*-type clade (Figure 6A). This suggests a close evolutionary relationship between phenolic acid dehydroxylating enzymes within both *Eggerthella* and *Gordonibacter*.

In contrast, some phylogenetically distant enzymes also process related substrates, suggesting convergent evolution. For example, *E. lenta* enzymes that dehydroxylate individual enantiomers of the same substrate (Cadh and eCadh) or dehydroxylate different *p*–OHs of the same molecule ((−)-Eldh1 and (−)-Eldh2) are interspersed across the *Eggerthella*-type clade (Figure 6A) and do not share high amino acid sequence identity (52% and 46%, respectively). Interestingly, different from the enantiocomplementary catechin metabolizing enzymes Cadh and eCadh, the enantiocomplementary pairs of lignan metabolizing enzymes (*Eggerthella* (+)-Eddh and *Gordonibacter* (−)-Eddh, and *Gordonibacter* (+)-Eldh1/2 and *Eggerthella* (−)-Eldh1/2) are from different genera (Figure 6A) and have distinct enzyme architectures (Figure S15B). The factors leading to these distinctive evolutionary patterns are unclear. Other examples of convergent evolution include *Eggerthella*-type and *Gordonibacter*-type enzymes that metabolize the same substrates (Hcdhs and Vadhs) (Figure 6A). Further efforts, including computational studies and mutagenesis experiments, are needed to elucidate the evolutionary trajectory of these enzymes and dissect the sequence determinants of substrate specificity.

### Variable Distribution of Catechol Dehydroxylases in Coriobacteriia Genomes

We next assessed the distribution of the eight newly characterized catechol dehydroxylases across sequenced Coriobacteriia isolates (Figures 6B and S16). First, there are clear genus-level distribution patterns regarding the enantiocomplementary catechol dehydroxylases. The catechin-metabolizing enzymes Cadh and eCadh are variably distributed in *Eggerthella* strains and other genera, but are not found in *Gordonibacter* (Figure 6B). Interestingly, Cadh typically co-occurs with eCadh in individual *Eggerthella* genomes, whereas eCadh can also be found in strains lacking Cadh, implying a broader availability of eCadh substrate(s) (Figure 6B). Among the lignan-metabolizing enzymes, the *Eggerthella*-type enzyme (+)-Eddh, which metabolizes (*S*,*S*)-**4**, is the most widely distributed across *Eggerthella* and multiple *Adlercreutzia* strains (Figure 6B). In contrast, its enantiocomplementary enzyme (−)-Eddh is found in many *Gordonibacter* strains (Figure 6B). Interestingly, the enantiocomplementary enzymes that metabolize the lactonized lignans have the opposite distribution pattern. *Gordonibacter*-type enzymes (+)-Eldh1 and (+)-Eldh2, which metabolize (*S*,*S*)-**5**, are found in most *Gordonibacter* species but are not present in *Eggerthella* species (Figure 6B). Their enantiocomplementary counterparts, *Eggerthella*-type (−)-Eldh1 and (−)-Eldh2 which metabolize (*R*,*R*)-**5** are found in *Eggerthella* species and other genera but are not encoded by *Gordonibacter* species (Figure 6B).

There are also striking differences in the distribution of the site-selective dehydroxylases. Only a few *Eggerthella* strains encode both (−)-Eldh1 and (−)-Eldh2, with most strains possessing only one of the two enzymes (Figure 6B). In contrast, *Gordonibacter* strains typically harbor both (+)-Eldh1 and (+)-Eldh2 in the same gene cluster (Figure 6B). The presence of these catechol dehydroxylases in different strains is consistent with the dehydroxylation capability of individual strains (Figure S17A–D).

Last, the genes encoding all newly discovered *Eggerthella* catechol dehydroxylases are genomically co-localized in individual *Eggerthella* genomes but are highly variably distributed across different strains (Figure 6C). The distribution is not correlated with strain phylogeny (Figure 6B), potentially suggestive of horizontal gene transfer. Whereas the hypervariable gene clusters encode different catechol dehydroxylases in individual *Eggerthella* strains, they have a similar architecture (Figure 6C), with accessory genes encoded at one end (Figure 6C). Within the gene cluster, each catechol dehydroxylase has its own transcriptional regulator, encoded in a divergent orientation to the genes encoding the three enzyme subunits (Figure 6C), which likely regulates the expression of individual enzymes in response to substrates. In total, the variable distribution of catechol dehydroxylases across different Coriobacteriia strains highlights the complex interaction between gut bacteria and dietary polyphenols and the need to consider strain-level variability when assessing gut bacterial polyphenol metabolism.

## Discussion

Catechol dehydroxylation is a prevalent reaction in gut bacterial polyphenol metabolism that impacts the bioavailability and bioactivity of polyphenols.^4,8^ Until recently the molecular basis of catechol dehydroxylation was uncharacterized, leading to difficulty in understanding and predicting the roles of the gut microbiome in modulating the health benefits of polyphenol consumption. Despite the discovery of the molybdenum-dependent catechol dehydroxylases, the lack of a feasible expression system for this enzyme family has been a major challenge impeding further characterization. In this study, we establish *G. urolithinfaciens* as a versatile host to express active catechol dehydroxylases and elucidate the roles of enzyme-specific accessory proteins required in their biogenesis. Using this expression system, we implement an enzyme discovery workflow to rapidly identify eight different catechol dehydroxylases from the human gut Coriobacteriia. These enzymes metabolize dietary catechins and lignans and display striking enantioselectivity and site-selectivity, highlighting how catechol dehydroxylases have evolved to process diverse plant polyphenols.

The heterologous expression system not only allowed us to obtain catechol dehydroxylase enzymes for functional characterization, but also to identify accessory proteins impacting their activity. While most accessory genes were important for Dadh activity but did not impact expression, the deletion of predicted helicase *dadhD* abolished Dadh expression. We propose that DadhD may interact with the Dadh genomic locus during transcription or the transcribed mRNA to promote protein expression (Figure S2C). DadhE has no functional annotation but its predicted interaction with the catalytic subunit and the presence of homologous proteins in other dehydroxylase gene clusters suggested it plays a critical role in enzyme biogenesis. DadhF is a TorD-like chaperone, which have been demonstrated to be important for expressing other DMSO reductase superfamily enzymes.^17^ It is potentially involved in cofactor insertion, protein translocation, or other processes.^17^ DadhG and DadhH, two membrane-associated ferredoxins, are predicted to interact with the DadhB and DadhC. Based on FoldSeek,^37^ they resemble ferredoxins NapG and NapH, respectively, found in the *E. coli* periplasmic nitrate reductase^38^ (Figure S18A,B), potentially mediating electron transfer similar to NapGH.^38^ Future functional characterization of these accessory proteins will help us further understand catechol dehydroxylase biogenesis and activity, and the evolution of this enzyme family within the larger DMSO reductase superfamily.

Most notably, we show that Coriobacteriia have evolved enzymes to dehydroxylate individual enantiomers of chiral polyphenols, adding to the collective knowledge of enantiocomplementary enzymes.^28^ There are a few examples of separate enzymes evolving to catalyze reactions on substrate enantiomers in which the reactive functional groups have opposite configuration, such as peptide methionine sulfoxide reductase MsrA that preferably reduces methionine-(*S*)-sulfoxide and MsrB that preferably reduces methionine-(*R*)-sulfoxide,^28,39^ and transaminases that distinguish l- and d-amino acids.^28,40^ There are more instances where different enzymes catalyze the same reaction on prochiral substrates with opposite enantioselectivity such as l- and d-lactate dehydrogenases,^28^ and flavin-dependent monooxygenases.^41^ However, examples of separate enzymes of similar or different enzyme architectures evolving to recognize stereochemistry of nonreactive substrate functional groups are distinct from the previous examples of naturally occurring enantiocomplementary enzymes and, to our knowledge, unprecedented. We hypothesize that the high substrate specificities of these catechol dehydroxylases arise from a strict preference for binding individual enantiomers, necessitating a separate enzyme for each substrate. To gain potential insights into the structural basis for enantiocomplentarity, we compared the AlphaFold2^42,43^-predicted structures of Cadh and eCadh and identified several residues that differ between their active sites (Figure S19A). The precise roles of these amino acids in enantioselectivity await further study. Further understanding of the mechanisms of catechol dehydroxylases and the features underlying their enantioselectivity may inspire efforts to design and engineer biocatalysts.

In addition, our findings show that Coriobacteriia have evolved separate enzymes to metabolize substrates containing multiple catechol groups, such as (*S*,*S*)-**5** and (*R*,*R*)-**5**. We hypothesize that this site-selectivity may also result from a strict requirement for substrate binding. We have identified several distinct residues in the predicted active sites of (+)-Eldh1 and (+)-Eldh2, which might play a role in site-selectivity (Figure S19B). The requirement for multiple selective enzymes to fully transform a substrate is reminiscent of reductive dehalogenation by organohalide-respiring bacteria. Multiple, highly selective reductive dehalogenases, which are also unevenly distributed across bacterial strains, perform regioselective reductive dehalogenation reactions of halogenated alkene and aromatic substrates.^44^ For example, *Dehalococcoides mccartyi* 195 use PceA and TceA to transform tetrachloroethene to trichloroethene and trichloroethene to vinyl chloride and ethene, respectively.^44−46^ Both catechol dehydroxylases and reductive dehalogenases allow bacteria to utilize organic molecules as terminal electron acceptors to support growth.^4,44^ This role in anaerobic respiration could potentially explain the driving force for evolving numerous diversified enzymes to process diverse substrates. Understanding the mechanisms and basis for selectivity in different catechol dehydroxylases will be needed to gain insights into their evolution and roles in microbial ecology.

The hypervariable distribution pattern of *Eggerthella* catechol dehydroxylases, which is not correlated with strain phylogeny (Figure 6B), raises additional interesting questions about the evolution of these enzymes. We hypothesize that the *Eggerthella* gene cluster encoding multiple catechol dehydroxylases might be a hot spot for enzyme duplication and diversification to create catechol dehydroxylases of new functions. For example, *Eggerthella guodeyinii* strain HF-1101 encodes nine different catechol dehydroxylases in this genomic region (Figure 6C). There are also likely hot spots for catechol dehydroxylase emergence in *Gordonibacter* species. For example, in *G. pamelaeae* DSM 19378, there are 11 uncharacterized catechol dehydroxylases encoded close to (−)-*eddh*, and the catechol dehydroxylases encoded in this region are also highly variably distributed across different *Gordonibacter* strains (Figure S20). A comparable distribution of high number of gene paralogs is also observed in *Dehalococcoides mccartyi* CBDB1 which encode 32 reductive dehalogenases, including 16 in a 97 kb region.^47^ These examples are supportive of the evolutionary advantages of evolving specialized enzymes for different substrates. Furthermore, the variation in the distribution of catechol dehydroxylase genes across different *Eggerthella* strains might result from horizontal gene transfer or variable gene loss driven by the availability of individual enzyme substrates from the diet. Efforts should be made to understand if and how horizontal gene transfer contributes to the dissemination of these dehydroxylases among different strains and genera. It also remains unclear how *Eggerthella* and *Gordonibacter* have each evolved their own type of catechol dehydroxylases and why there appears to be no transfer of catechol dehydroxylase encoding genes between the two genera. Ancestral sequence reconstruction leveraging our heterologous expression system^48^ may be a powerful strategy to understand catechol dehydroxylase evolution and identify key amino acid residues responsible for selectivity.^41,48,49^

Overall, our work reveals how a prevalent class of human gut bacteria can metabolize a wide diversity of dietary polyphenols, including multiple enantiomeric substrate pairs. It is likely that the large variation in catechol dehydroxylase activity across these gut bacteria contributes to inter-individual variability in polyphenol metabolism. The genetic tools we have employed for characterizing catechol dehydroxylases and the accelerated identification of enzymes critical for lignan and catechin metabolism will therefore enable a better understanding, prediction, and modulation of the health benefits of these plant-derived polyphenols.

## Data Availability

All other data generated or analyzed during this study are included in this article and its supplementary files. Source data are provided with this paper. The plasmids used in this study will be available from Addgene upon publication of the manuscript. Further information and requests for resources and reagents should be directed to and will be fulfilled by the Lead Contact, Emily P. Balskus (balskus@chemistry.harvard.edu).

## Supporting Information Available

Full description of materials, detailed procedures for bacterial culture, plasmid construction, bacterial transformations, biochemical assays, metabolite analysis, and bioinformatic analysis (Figures S1–S20) (PDF)
Strains and plasmids (Table S1), oligonucleotide sequences used in the study (Table S2), Coriobacteriia genome accession numbers (Table S3), catechol dehydroxylase accession numbers (Table S4), and amino acid identity% (Table S5) (XLSX)

## References

[ref1] Pandey K. B.; Rizvi S. I. Plant Polyphenols as Dietary Antioxidants in Human Health and Disease. Oxid. Med. Cell. Longev. 2009, 2, 270–278. 10.4161/oxim.2.5.9498. 20716914 PMC2835915

[ref2] Cory H.; Passarelli S.; Szeto J.; Tamez M.; Mattei J. The Role of Polyphenols in Human Health and Food Systems: A Mini-Review. Front. Nutr. 2018, 5, 370438 10.3389/fnut.2018.00087. PMC6160559 30298133

[ref3] Zhou Y.; Zheng J.; Li Y.; Xu D. P.; Li S.; Chen Y. M.; Li H. B. Natural Polyphenols for Prevention and Treatment of Cancer. Nutrients 2016, 8 (8), 515 10.3390/nu8080515. 27556486 PMC4997428

[ref4] Maini Rekdal V.; Nol Bernadino P.; Luescher M. U.; Kiamehr S.; Le C.; Bisanz J. E.; Turnbaugh P. J.; Bess E. N.; Balskus E. P. A widely distributed metalloenzyme class enables gut microbial metabolism of host- and diet-derived catechols. Elife 2020, 9, e50845 10.7554/eLife.50845. 32067637 PMC7028382

[ref5] DeEds F.; Booth A. N.; Jones F. T. Methylation and Dehydroxylation of Phenolic Compounds by Rats and Rabbits. J. Biol. Chem. 1957, 225 (2), 615–621. 10.1016/S0021-9258(18)64860-4. 13416264

[ref6] Scheline R. R.; Williams R. T.; Wit J. G. Biological Dehydroxylation. Nature 1960, 188 (4753), 849–850. 10.1038/188849a0. 13747445

[ref7] Jin J. S.; Zhao Y. F.; Nakamura N.; Akao T.; Kakiuchi N.; Min B. S.; Hattori M. Enantioselective dehydroxylation of enterodiol and enterolactone precursors by human intestinal bacteria. Biol. Pharm. Bull. 2007, 30 (11), 2113–2119. 10.1248/bpb.30.2113. 17978485

[ref8] Bess E. N.; Bisanz J. E.; Yarza F.; Bustion A.; Rich B. E.; Li X.; Kitamura S.; Waligurski E.; Ang Q. Y.; Alba D. L. Genetic basis for the cooperative bioactivation of plant lignans by Eggerthella lenta and other human gut bacteria. Nat. Microbiol. 2020, 5 (1), 56–66. 10.1038/s41564-019-0596-1. 31686027 PMC6941677

[ref9] Takagaki A.; Kato Y.; Nanjo F. Isolation and characterization of rat intestinal bacteria involved in biotransformation of (−)-epigallocatechin. Arch. Microbiol. 2014, 196 (10), 681–695. 10.1007/s00203-014-1006-y. 24947740

[ref10] Takagaki A.; Nanjo F. Catabolism of (+)-catechin and (−)-epicatechin by rat intestinal microbiota. J. Agric. Food Chem. 2013, 61 (20), 4927–4935. 10.1021/jf304431v. 23621128

[ref11] Takagaki A.; Nanjo F. Biotransformation of (−)-epigallocatechin and (−)-gallocatechin by intestinal bacteria involved in isoflavone metabolism. Biol. Pharm. Bull. 2015, 38 (2), 325–330. 10.1248/bpb.b14-00646. 25747993

[ref12] Maini Rekdal V.; Bess E. N.; Bisanz J. E.; Turnbaugh P. J.; Balskus E. P. Discovery and inhibition of an interspecies gut bacterial pathway for Levodopa metabolism. Science 2019, 364 (6445), eaau6323 10.1126/science.aau6323. 31196984 PMC7745125

[ref13] Sandler M.; Karoum F.; Ruthven C. R. J.; Calne D. B. *m*-Hydroxyphenylacetic Acid Formation from L-Dopa in Man: Suppression by Neomycin. Science 1969, 166 (3911), 1417–1418. 10.1126/science.166.3911.1417. 5350345

[ref14] Sandler M.; Goodwin B. L.; Ruthven C. R. J.; Calne D. B. Therapeutic Implications in Parkinsonism of m-Tyramine Formation from L-Dopa in Man. Nature 1971, 229 (5284), 414–416. 10.1038/229414a0. 4926994

[ref15] Le C. C.; Bae M.; Kiamehr S.; Balskus E. P. Emerging Chemical Diversity and Potential Applications of Enzymes in the DMSO Reductase Superfamily. Annu. Rev. Biochem. 2022, 91, 475–504. 10.1146/annurev-biochem-032620-110804. 35320685

[ref16] Dong X.; Guthrie B. G. H.; Alexander M.; Noecker C.; Ramirez L.; Glasser N. R.; Turnbaugh P. J.; Balskus E. P. Genetic manipulation of the human gut bacterium Eggerthella lenta reveals a widespread family of transcriptional regulators. Nat. Commun. 2022, 13 (1), 7624 10.1038/s41467-022-33576-3. 36494336 PMC9734109

[ref17] Genest O.; Mejean V.; Iobbi-Nivol C. Multiple roles of TorD-like chaperones in the biogenesis of molybdoenzymes. FEMS Microbiol. Lett. 2009, 297 (1), 1–9. 10.1111/j.1574-6968.2009.01660.x. 19519768

[ref18] Little A. S.; Younker I. T.; Schechter M. S.; Bernardino P. N.; Meheust R.; Stemczynski J.; Scorza K.; Mullowney M. W.; Sharan D.; Waligurski E. Dietary- and host-derived metabolites are used by diverse gut bacteria for anaerobic respiration. Nat. Microbiol. 2024, 9 (1), 55–69. 10.1038/s41564-023-01560-2. 38177297 PMC11055453

[ref19] Bae M.; Le C.; Mehta R. S.; Dong X.; Pieper L. M.; Ramirez L.; Alexander M.; Kiamehr S.; Turnbaugh P. J.; Huttenhower C.; et al. Metatranscriptomics-guided discovery and characterization of a polyphenol-metabolizing gut microbial enzyme. Cell Host Microbe 2024, 32, 1887–1896.e8. 10.1016/j.chom.2024.10.002. 39471822 PMC11585353

[ref20] Finefield J. M.; Sherman D. H.; Kreitman M.; Williams R. M. Enantiomeric natural products: Occurrence and biogenesis. Angew. Chem., Int. Ed. 2012, 51 (20), 4802–4836. 10.1002/anie.201107204. PMC3498912 22555867

[ref21] Jin J. S.; Kakiuchi N.; Hattori M. Enantioselective oxidation of enterodiol to enterolactone by human intestinal bacteria. Biol. Pharm. Bull. 2007, 30 (11), 2204–2206. 10.1248/bpb.30.2204. 17978502

[ref22] McCurry M. D.; D’Agostino G. D.; Walsh J. T.; Bisanz J. E.; Zalosnik I.; Dong X.; Morris D. J.; Korzenik J. R.; Edlow A. G.; Balskus E. P.; et al. Gut bacteria convert glucocorticoids into progestins in the presence of hydrogen gas. Cell 2024, 187 (12), 2952–2968.e13. 10.1016/j.cell.2024.05.005. 38795705 PMC11179439

[ref23] Abramson J.; Adler J.; Dunger J.; Evans R.; Green T.; Pritzel A.; Ronneberger O.; Willmore L.; Ballard A. J.; Bambrick J. Accurate structure prediction of biomolecular interactions with AlphaFold 3. Nature 2024, 630 (8016), 493–500. 10.1038/s41586-024-07487-w. 38718835 PMC11168924

[ref24] Sakakibara H.; Honda Y.; Nakagawa S.; Ashida H.; Kanazawa K. Simultaneous Determination of All Polyphenols in Vegetables, Fruits, and Teas. J. Agric. Food Chem. 2003, 51 (3), 571–581. 10.1021/jf020926l. 12537425

[ref25] Shabbir U.; Rubab M.; Daliri E. B.-M.; Chelliah R.; Javed A.; Oh D. H. Curcumin, Quercetin, Catechins and Metabolic Diseases: The Role of Gut Microbiota. Nutrients 2021, 13 (1), 206 10.3390/nu13010206. 33445760 PMC7828240

[ref26] Takagaki A.; Nanjo F. Bioconversion of (−)-Epicatechin, (+)-Epicatechin, (−)-Catechin, and (+)-Catechin by (−)-Epigallocatechin-Metabolizing Bacteria. Biol. Pharm. Bull. 2015, 38 (5), 789–794. 10.1248/bpb.b14-00813. 25947926

[ref27] Wang L.-Q.; Meselhy M. R.; Li Y.; Nakamura N.; Min B.-S.; Qin G.-W.; Hattori M. The Heterocyclic Ring Fission and Dehydroxylation of Catechins and Related Compounds by Eubacterium sp. Strain SDG-2, a Human Intestinal Bacterium. Chem. Pharm. Bull. 2001, 49 (12), 1640–1643. 10.1248/cpb.49.1640. 11767089

[ref28] Mugford P. F.; Wagner U. G.; Jiang Y.; Faber K.; Kazlauskas R. J. Enantiocomplementary enzymes: Classification, molecular basis for their enantiopreference, and prospects for mirror-image biotransformations. Angew. Chem., Int. Ed. 2008, 47 (46), 8782–8793. 10.1002/anie.200705159. 18850616

[ref29] Clavel T.; Borrmann D.; Braune A.; Dore J.; Blaut M. Occurrence and activity of human intestinal bacteria involved in the conversion of dietary lignans. Anaerobe 2006, 12 (3), 140–147. 10.1016/j.anaerobe.2005.11.002. 16765860

[ref30] Woting A.; Clavel T.; Loh G.; Blaut M. Bacterial transformation of dietary lignans in gnotobiotic rats. FEMS Microbiol. Ecol. 2010, 72 (3), 507–514. 10.1111/j.1574-6941.2010.00863.x. 20370826

[ref31] Blaut M.; Clavel T. Metabolic Diversity of the Intestinal Microbiota: Implications for Health and Disease1. J. Nutr. 2007, 137 (Suppl 3), 751S–755S. 10.1093/jn/137.3.751S. 17311972

[ref32] Jin J. S.; Hattori M. Human intestinal bacterium, strain END-2 is responsible for demethylation as well as lactonization during plant lignan metabolism. Biol. Pharm. Bull. 2010, 33 (8), 1443–1447. 10.1248/bpb.33.1443. 20686246

[ref33] Jin J. S.; Zhao Y. F.; Nakamura N.; Akao T.; Kakiuchi N.; Hattori M. Isolation and characterization of a human intestinal bacterium, Eubacterium sp. ARC-2, capable of demethylating arctigenin, in the essential metabolic process to enterolactone. Biol. Pharm. Bull. 2007, 30 (5), 904–911. 10.1248/bpb.30.904. 17473433

[ref34] Jin J. S.; Hattori M. Further studies on a human intestinal bacterium Ruminococcus sp. END-1 for transformation of plant lignans to mammalian lignans. J. Agric. Food Chem. 2009, 57 (16), 7537–7542. 10.1021/jf900902p. 19630415

[ref35] Xie L. H.; Akao T.; Hamasaki K.; Deyama T.; Hattori M. Biotransformation of pinoresinol diglucoside to mammalian lignans by human intestinal microflora, and isolation of Enterococcus faecalis strain PDG-1 responsible for the transformation of (+)-pinoresinol to (+)-lariciresinol. Chem. Pharm. Bull. 2003, 51 (5), 508–515. 10.1248/cpb.51.508. 12736449

[ref36] Asnicar F.; Thomas A. M.; Beghini F.; Mengoni C.; Manara S.; Manghi P.; Zhu Q.; Bolzan M.; Cumbo F.; May U.; et al. Precise phylogenetic analysis of microbial isolates and genomes from metagenomes using PhyloPhlAn 3.0. Nat. Commun. 2020, 11 (1), 2500 10.1093/jn/137.3.751S. 32427907 PMC7237447

[ref37] van Kempen M.; Kim S. S.; Tumescheit C.; Mirdita M.; Lee J.; Gilchrist C. L. M.; Soding J.; Steinegger M. Fast and accurate protein structure search with Foldseek. Nat. Biotechnol. 2024, 42 (2), 243–246. 10.1038/s41587-023-01773-0. 37156916 PMC10869269

[ref38] Sparacino-Watkins C.; Stolz J. F.; Basu P. Nitrate and periplasmic nitrate reductases. Chem. Soc. Rev. 2014, 43 (2), 676–706. 10.1039/C3CS60249D. 24141308 PMC4080430

[ref39] Delaye L.; Becerra A.; Orgel L.; Lazcano A. Molecular evolution of peptide methionine sulfoxide reductases (MsrA and MsrB): On the early development of a mechanism that protects against oxidative damage. J. Mol. Evol. 2007, 64 (1), 15–32. 10.1007/s00239-005-0281-2. 17180746

[ref40] Sugio S.; Petsko G. A.; Manning J. M.; Soda K.; Ringe D. Crystal structure of a D-amino acid aminotransferase: How the protein controls stereoselectivity. Biochemistry 1995, 34 (30), 9661–9669. 10.1021/bi00030a002. 7626635

[ref41] Chiang C. H.; Wymore T.; Rodriguez Benitez A.; Hussain A.; Smith J. L.; Brooks C. L. 3rd; Narayan A. R. H. Deciphering the evolution of flavin-dependent monooxygenase stereoselectivity using ancestral sequence reconstruction. Proc. Natl. Acad. Sci. U. S. A. 2023, 120 (15), e2218248120 10.1073/pnas.2218248120. 37014851 PMC10104550

[ref42] Jumper J.; Evans R.; Pritzel A.; Green T.; Figurnov M.; Ronneberger O.; Tunyasuvunakool K.; Bates R.; Žídek A.; Potapenko A. Highly accurate protein structure prediction with AlphaFold. Nature 2021, 596 (7873), 583–589. 10.1038/s41586-021-03819-2. 34265844 PMC8371605

[ref43] Mirdita M.; Schutze K.; Moriwaki Y.; Heo L.; Ovchinnikov S.; Steinegger M. ColabFold: Making protein folding accessible to all. Nat. Methods 2022, 19 (6), 679–682. 10.1038/s41592-022-01488-1. 35637307 PMC9184281

[ref44] Fincker M.; Spormann A. M. Biochemistry of Catabolic Reductive Dehalogenation. Annu. Rev. Biochem. 2017, 86, 357–386. 10.1146/annurev-biochem-061516-044829. 28654328

[ref45] Fung J. M.; Morris R. M.; Adrian L.; Zinder S. H. Expression of reductive dehalogenase genes in Dehalococcoides ethenogenes strain 195 growing on tetrachloroethene, trichloroethene, or 2,3-dichlorophenol. Appl. Environ. Microbiol. 2007, 73 (14), 4439–4445. 10.1128/AEM.00215-07. 17513589 PMC1932842

[ref46] Magnuson J. K.; Stern R. V.; Gossett J. M.; Zinder S. H.; Burris D. R. Reductive dechlorination of tetrachloroethene to ethene by a two-component enzyme pathway. Appl. Environ. Microbiol. 1998, 64 (4), 1270–1275. 10.1128/AEM.64.4.1270-1275.1998. 10671186 PMC106140

[ref47] Kube M.; Beck A.; Zinder S. H.; Kuhl H.; Reinhardt R.; Adrian L. Genome sequence of the chlorinated compound-respiring bacterium Dehalococcoides species strain CBDB1. Nat. Biotechnol. 2005, 23 (10), 1269–1273. 10.1038/nbt1131. 16116419

[ref48] Merkl R.; Sterner R. Ancestral protein reconstruction: Techniques and applications. Biol. Chem. 2016, 397 (1), 1–21. 10.1515/hsz-2015-0158. 26351909

[ref49] Nocedal I.; Laub M. T. Ancestral reconstruction of duplicated signaling proteins reveals the evolution of signaling specificity. Elife 2022, 11, e77346 10.7554/eLife.77346. 35686729 PMC9208753

