# Supplementary material for: Enantiocomplementary Gut Bacterial Enzymes Metabolize Dietary Polyphenols
Source: J Am Chem Soc. 2025 Feb 24;147(9):7231–44. doi: 10.1021/jacs.4c09892 (PMC11887054; doi:10.1021/jacs.4c09892)
Supplement: Supplementary file 1 — ja4c09892_si_001.pdf [file ja4c09892_si_001.pdf]

SUPPORTING INFORMATION

Enantiocomplementary gut bacterial enzymes metabolize dietary polyphenols

Xueyang Dong[1], Minwoo Bae[1], Chi (Chip) Le[1], Miguel A. Aguilar Ramos[1], Emily P. Balskus[1,2*]

[1]Department of Chemistry and Chemical Biology, Harvard University, Cambridge, MA 02138, United States

[2]Howard Hughes Medical Institute, Harvard University, Cambridge, MA 02138, United States

Contents

Methods 4

Bacterial culture 4

Plasmids and DNA oligonucleotides 4

Bacterial transformations 5

Assays for measuring metabolism of dopamine, hydrocaffeic acid, (+)-catechin, (–)-epicatechin, (*R*)-**1**, (*S*)-**1**, (*R*)-**2**, (*S*)-**2** and **3** by Coriobacteriia WT, engineered *G. uro* and *E. coli* strains 5

Assays for measuring metabolism of (*S*,*S*)-**4**, (*R*,*R*)-**4**, (*S*,*S*)-**5** and (*R*,*R*)-**5** by Coriobacteriia WT, engineered *G. uro* and engineered *E. lenta* strains 8

Catechol dehydroxylase purification 10

Western blotting 11

Measurement of *E. lenta* catechol dehydroxylase substrate specificity 11

Culturing *E. lenta* and *G. pamelaeae* with catechols, RNA extraction and RT-qPCR experiments 12

Phylogenetic analysis of Coriobacteriia genomes and catechol dehydroxylases 13

Protein structural analysis 14

Quantification and statistical analysis 14

Supplementary Figures 15

Figure S1. Heterologous expression of Dadh in *G. uro* and putative roles of Dadh accessory genes. 15

Figure S2. Putative roles of accessory genes in *Eggerthella* catechol dehydroxylases. 16

Figure S3. Heterologous expression of Hcdh and Cadh in *G. uro* and specificity of accessory genes. 17

Figure S4. Heterologous expression of Dadh, Hcdh and Cadh in *E. coli* 19

Figure S5. Preparation of catechol dehydroxylase substrates. 20

Figure S6. Metabolism of (+)-catechin and (–)-epicatechin by different Coriobacteriia WT strains, and identification and characterization of eCadh. 21

Figure S7. Metabolism of (*S*)-**2**, (*R*)-**2** and **3** by different Coriobacteriia WT strains. 22

Figure S8. Identification and characterization of (*S*)-**2,** (*R*)-**2**, and **3** dehydroxylases. 23

Figure S9. Summary of the characterized gut bacterial metabolism of (+)-catechin and (–)-epicatechin. 25

Figure S10. Metabolism of (*S*,*S*)-**4** by different Coriobacteriia WT strains, and identification and characterization of (+)-Eddh. 26

Figure S11. Metabolism of (*R*,*R*)-**4** by different Coriobacteriia WT strains, and characterization of (–)-Eddh. 27

Figure S12. Gut bacterial metabolism related to **5**, metabolism of (*R*,*R*)-**5** by different Coriobacteriia WT strains, and identification of (–)-Eldh1 and (–)-Eldh2. 28

Figure S13. MS fragmentation of (*R*,*R*)-**11** and (*R*,*R*)-**12**. 30

Figure S14. Metabolism of (*S*,*S*)-**5** by different Coriobacteriia WT strains, identification of (*S*,*S*)-**5** dehydroxylases and MS fragmentation of (*S*,*S*)-**11** and (*S*,*S*)-**12**. 31

Figure S15. Substrate specificity of co-localized *E. lenta* catechol dehydroxylases, and summary of characterized catechol dehydroxylases in this study. 33

Figure S16. Distribution of catechol dehydroxylases in different Coriobacteriia strains with strain information specified. 34

Figure S17. Presence of catechol dehydroxylases in different Coriobacteriia strains predicts the metabolism. 35

Figure S18. DadhG/H share similar structures to NapG/H. 36

Figure S19. Active site comparison of enantiocomplementary and complementary site-selective catechol dehydroxylases. 37

Figure S20. Hypervariable genomic regions encoding catechol dehydroxylases in different *Gordonibacter* strains. 38

Supplemental references 39

Methods

Bacterial culture

Routine culturing of *E. lenta*, *E. timonensis*, *E. sinensis*, *G. pamelaeae* and *G. urolithinfaciens* was done under anaerobic conditions (Coy Lab Products) with an atmosphere of 2% to 4% H_2, 2% to 4% CO_2, and N_2 as the balance. *Eggerthella* and *Gordonibacter* cultures were grown in BHIrcf medium (BHI with 1% w/v arginine, 0.05% w/v L-cysteine hydrochloride and 10 mM sodium formate) at 37 °C unless otherwise stated. For *E. lenta* DSM 2243 and *G. uro* transformed with kanamycin-resistance plasmids, cultures were grown in BHIrcf supplemented with 100 µg/mL of kanamycin unless otherwise stated.

Plasmids and DNA oligonucleotides

Cloning work was performed using Gibson Assembly (NEB, E2611S) or NEBuilder HiFi DNA Assembly (NEB, E2621S). The plasmids involved in this work are listed in Supplementary Table 1. The oligonucleotide sequences used for this work are listed in Supplementary Table 2.

To construct an entry plasmid for enzyme expression in *G. uro*, we first introduced tandem terminators flanking the expression cassette into the previously reported *G. uro* vector pXD68Kan2 (https://www.addgene.org/191248/) to prevent possible interference between the expression cassette and other genes on the plasmid. The plasmid pXD68Kan2 backbone was first amplified using primers oXD855 and oXD856, and was ligated to a fragment containing tandem terminators amplified from pXD71Cas10RFP (https://www.addgene.org/192273/) using primers oXD859 and oXD860 to construct plasmid pXD80RFP. The resulting plasmid pXD80RFP was then amplified with primers oXD291 and oXD292 to provide the pXD80RFP plasmid backbone containing terminators flanking the expression cassette.

Briefly, to construct the catechol dehydroxylase-expressing plasmids for expression in *G. uro*, the coding sequences of the regulator, three enzyme subunits, and the flanked promoter region were amplified in one piece or two pieces from either *E. lenta* A2 or DSM 2243 and cloned into the pXD80RFP backbone. To include accessory genes in the catechol dehydroxylase plasmids, either the PsiI-v2 (NEB, R0744S) or SpeI-HF (NEB, R3133S) restriction endonuclease was used to digest the original catechol dehydroxylase-expressing plasmids. The accessory genes were amplified in one piece or two pieces from *E. lenta* A2 and ligated into the linearized catechol dehydroxylase plasmid backbone. To construct the Elen_0616 plasmid, the coding sequences of the regulator, enzyme and the flanked promoter region were amplified from *E. lenta* DSM 2243 and cloned into the pXD80RFP backbone. To construct the C1878_02750 plasmid, the gene cluster of C1878_02750 was amplified from *Gordonibacter* sp. 28C and cloned into the pXD80RFP backbone.

To construct the (+)-Eldh1/2-encoding plasmids, the coding sequences of (+)-Eldh1AB, (+)-Eldh2AB or both were amplified from *G. pamelaeae* DSM 19378 gDNA and cloned to replace the *lacZ* gene in pXD70CT5.

Bacterial transformations

Preparation of electrocompetent cells and transformation of various *E. lenta* strains and *Gordonibacter* species was performed as previously described[1]. Briefly, for preparation of *E. lenta* or *G. uro* electrocompetent cells, 1 mL of 48 h saturated cultures was inoculated into 100 mL of BHI+ medium (BHI with 1% w/v arginine) and grown at 37 °C to an OD_{600} of 0.2–0.4. Cultures were chilled on ice for 20 min, centrifuged, washed three times with 10 mL of ice-cold water, and washed once with 5 mL of 10% ice-cold aqueous glycerol solution. The cell pellets were suspended in 2–4 mL of 10% aqueous glycerol solution. For bacterial transformation, 100-µL aliquots of *E. lenta* or *G. uro* electrocompetent cells were electroporated using a MicroPulser Electroporator (BioRad) with 500–2000 ng of plasmid at 2.5 kV voltage, using 1-mm gap width electroporation cuvettes (VWR). 1 mL of BHIrcf medium was immediately added to the electroporated cells and transferred to 1.7-mL Eppendorf tubes. These tubes were brought to an anaerobic chamber (Coy) and incubated at 37 °C anaerobically for 3 h. All of the transformations were plated onto BHIrf (BHI with 1% w/v arginine and 10 mM sodium formate) agar with 100 µg/mL kanamycin and grown anaerobically for 3–4 days at 37 °C.

Assays for measuring metabolism of dopamine, hydrocaffeic acid, (+)-catechin, (–)-epicatechin, (*R*)-1, (*S*)-1, (*R*)-2, (*S*)-2 and 3 by Coriobacteriia WT, engineered *G. uro* and *E. coli* strains

Dopamine hydrochloride (Sigma H8502, ≥ 98% purity) was dissolved in BHIrcf to make a 200 mM stock solution. Hydrocaffeic acid (Sigma 102601, ≥ 98% purity) was dissolved in sterile H_2O to make a 400 mM stock solution. (+)-Catechin (Sigma C1251, ≥ 98% purity) and (–)-epicatechin (Sigma E1753, ≥ 90% purity) were dissolved in DMF to make 200 mM stock solutions, which were used to generate (*R*)-**1**/(*S*)-**2** and (*S*)-**1**/(*R*)-**2**, respectively, as described below. **3** (Toronto Research Chemicals, TRC-D458505, > 95% purity) was dissolved in DMF to make a 20 mM stock solution.

To obtain (*R*)-**1** and (*S*)-**1**, saturated cultures of *E. lenta* AB8n2 in BHIrcf were inoculated 1:20 into 20 mL of fresh BHIrcf medium containing 1 mM (+)-catechin or 1 mM (–)-epicatechin and incubated at 37 °C anaerobically for 72 h as previously reported[2-4], with > 90% substrate consumption. The production of (*R*)-**1** and (*S*)-**1** was confirmed using LC–MS/MS as described below. The stereochemistry of (*R*)-**1**, derived from (+)-catechin, and (*S*)-**1**, derived from (–)-epicatechin, and their dehydroxylated products was assigned based on previous reports that showed the configurations at the 3-position of catechins are maintained during gut bacterial metabolism based

on NMR, LC-MS/MS and optical rotation analysis[2-4]. The optical purity of our prepared substrates was further supported by the distinct metabolism patterns displayed by the heterologous strains expressing Cadh and eCadh (Fig. S6G, S6H). The spent medium after incubation was centrifuged at 3220× *g* for 15 min at 4 °C in a tabletop swinging bucket centrifuge, filtered through a 0.22 μm syringe filter (VWR) twice, and then diluted 2-fold or 4-fold into fresh BHIrcf medium.

To obtain (*S*)-**2** and (*R*)-**2**, saturated cultures of *F. plautii* DSM 4000 and *E. lenta* AB8n2 in GAM medium (Gifu Anaerobic Broth) were inoculated 1:50 into 20 mL of fresh GAM medium containing 2 mM (+)-catechin or 2 mM (–)-epicatechin and incubated at 37 °C anaerobically for 90 h as previously reported[2-4], with > 90% substrate consumption. The production of (*S*)-**2**, derived from (+)-catechin, and (*R*)-**2**, derived from (–)-epicatechin was confirmed using LC–MS/MS as described below. The stereochemistry of (*S*)-**2**, (*R*)-**2**, and their dehydroxylated products was assigned based on previous reports that the configurations at the 3-position of the original catechins are maintained during gut bacterial metabolism based on NMR, LC-MS/MS and optical rotation analysis[2-4]. The optical purity of our prepared substrates was further supported by the distinct specificities of the purified *El* Vadh and *Gs* Vadh enzymes (Fig. S8I, S8K). The spent medium after incubation was centrifuged at 3220× *g* for 15 min at 4 °C on a tabletop swinging bucket centrifuge, filtered through a 0.22 μm syringe filter (VWR) twice and then diluted 2-fold or 4-fold into fresh BHIf (BHI with 10 mM sodium formate) medium. The diluted cell free supernatant (CFS) was used to grow Coriobacteriia strains to test metabolism of (*R*)-**1**, (*S*)-**1**, (*R*)-**2** and (*S*)-**2**.

Coriobacteriia WT strains were grown to saturation in BHIrcf media, and the engineered *G. uro* strains were grown to saturation in BHIrcf with 100 μg/mL of kanamycin. The saturated WT cultures were then inoculated 1:20, 1:25, or 1:50 either into 200 μL of BHIrcf supplemented with 1 mM dopamine, 1 mM hydrocaffeic acid, 1 mM (+)-catechin or 1 mM (–)-epicatechin, respectively, into 200 μL of BHIf supplemented with 100 μM **3**, or into 200 μL of diluted CFS containing (*R*)-**1**, (*S*)-**1**, (*S*)-**2** or (*R*)-**2** substrate in triplicate in 96-well clear flat-bottom microplates (Corning or Avantor). For engineered *G. uro* strains, 100 μg/mL of kanamycin was added to the growth medium. The cultures were incubated at 37 °C anaerobically for 48–96 h. For assessing the heterologous expression in *E. coli*, *E. coli* DH5α strains harboring the catechol dehydroxylase plasmids were used along with engineered *G. uro* strains harboring the same plasmids in the activity assays.

The plates were then centrifuged (3220× *g*, 10 min, 4 °C), and the supernatants were harvested. For detection of dopamine and hydrocaffeic acid metabolites, 40 μL of the supernatant was diluted 1:5 with 160 μL of LC–MS grade (Honeywell) methanol, and 40 μL of the resulting mixture was then diluted 1:5 with 160 μL of Milli-Q water. For detection of (+)-catechin, (–)-epicatechin, (*R*)-**1**, (*S*)-**1**, (*R*)-**2**, (*S*)-**2**, and **3** metabolites, 40 μL of the supernatant was diluted 1:5 with 160 μL of LC–MS grade (Honeywell) methanol, and 100 μL of the resulting mixture was then diluted 1:2 with 100

μL of Milli-Q water. The amount of catechols and products in the diluted mixtures were quantified using UPLC–MS/MS.

UPLC–MS/MS was conducted using a Waters Acquity UPLC H-Class System (Waters Corporation), and Waters Xevo TQ-S (Waters Corporation) instrument. 5 or 10 μL of each sample was injected onto a CORTECS T3 Column (120Å, 2.7 μm, 2.1 mm X 100 mm, Waters Corporation). The flow rate was 0.5 mL/min using solvent A = 0.1% formic acid in H_2O and solvent B = 0.1% formic acid in acetonitrile (Honeywell). The column temperature was maintained at 40 °C. The following gradient was applied: 0–1 min at 100% A isocratic, 1.0–2.0 min at 0–90% B, 2.0–2.5 min at 90% B isocratic, 2.5–2.75 min at 90–0% B, 2.75–3.50 min at 0% B isocratic.

MS detection of dopamine and *m*-tyramine was performed using electron spray ionization in positive mode (ESI+) (capillary voltage, 3.20 kV; cone voltage, 29 V; source offset voltage, 50 V; desolvation temperature, 200 °C; desolvation gas flow, 800 L/h; cone gas flow, 150 L/h; nebulizer, 7.0 bar). The masses of dopamine (precursor ion *m*/*z* = 154.1119, daughter ion *m*/*z* = 137.0550; cone voltage 30 V; collision energy 8 V), and *m*-tyramine (precursor ion *m*/*z* = 138.1119, daughter ion *m*/*z* = 121.1114; cone voltage 8 V; collision energy 10 V) were monitored[5]. Concentrations of dopamine and *m*-tyramine were quantified using calibration curves.

MS detection of all other metabolites was performed using electron spray ionization in negative mode (ESI–) (capillary voltage, 2.60 kV; cone voltage, 29 V; source offset voltage, 50 V; desolvation temperature, 200 °C; desolvation gas flow, 800 L/h; cone gas flow, 150 L/h; nebulizer, 7.0 bar).

The masses of hydrocaffeic acid (precursor ion *m*/*z* = 181.0898, daughter ion *m*/*z* = 137.1128; cone voltage 2 V; collision energy 12 V), and *m*-HPPA (precursor ion *m*/*z* = 165.0919, daughter ion *m*/*z* = 106.0780; cone voltage 36 V; collision energy 20 V) were monitored[2]. Concentrations of hydrocaffeic acid and *m*-HPPA were quantified using calibration curves.

To detect metabolism of (+)-catechin, (–)-epicatechin, and **2**, the masses of (+)-catechin or (–)-epicatechin (precursor ion *m*/*z* = 289.0710, daughter ion *m*/*z* = 109.0300; cone voltage 45 V; collision energy 20 V), C–O cleavage product **1** (precursor ion *m*/*z* = 291.0860, daughter ion *m*/*z* = 123.0440; cone voltage 45 V; collision energy 20 V), dehydroxylated product **6** (precursor ion *m*/*z* = 275.0910, daughter ion *m*/*z* = 107.0490; cone voltage 45 V; collision energy 20 V), **2** (precursor ion *m*/*z* = 225.0760, daughter ion *m*/*z* = 101.0000; cone voltage 30 V; collision energy 15 V) and **7** (precursor ion *m*/*z* = 209.0810, daughter ion *m*/*z* = 101.0000; cone voltage 30 V; collision energy 15 V) were monitored, based on previous reports[2-4] with further optimization.

For detecting metabolism of **3**, the masses of **3** (precursor ion *m*/*z* = 209.0810, daughter ion *m*/*z* = 122.0360; cone voltage 30 V; collision energy 20 V) and **8** (precursor ion *m*/*z* = 193.0860, daughter ion *m*/*z* = 149.0960; cone voltage 30 V; collision energy 17 V) were monitored, based on a previous report[3] with further optimization.

Assays for measuring metabolism of (*S*,*S*)-4, (*R*,*R*)-4, (*S*,*S*)-5 and (*R*,*R*)-5 by Coriobacteriia WT, engineered *G. uro* and engineered *E. lenta* strains

(+)-SECO (AstaTech, E87134-10MG, ≥ 98% purity), (–)-arctigenin (Biosynth, FA41645-0.5G, > 98% purity), and (+)-matairesinol (BLD Pharmatech, BD298610, > 98% purity) were used to prepare (*S*,*S*)-**4**, (*R*,*R*)-**4**/**5**, and (*S*,*S*)-**5**, respectively.

To obtain (*S*,*S*)-**4**, a *B. producta* DSM 3507 culture was inoculated directly from a glycerol stock into 30 mL of BHIrcf medium containing 250 μM (+)-SECO (AstaTech, E87134-10MG) and incubated at 37 °C anaerobically for 144 h as previously reported[6], with > 90% substrate consumption. The production of (*S*,*S*)-**4** was confirmed using LC–MS/MS as described below. (*R*,*R*)-**4** was chemically synthesized from (–)-arctigenin (Biosynth, FA41645-0.5G) according to a previously published protocol[7] and dissolved in DMF to make a 10 mM stock solution. The stereochemistry of (*S*,*S*)-**4**, derived from (+)-SECO, and (*R*,*R*)-**4**, derived from (–)-arctigenin, was assigned based on previous reports[6, 7] that no transformations of the compound stereocenters occur under the preparation condition, and their optical purity was further supported by the distinct metabolism patterns observed for strains of the *Eggerthella* and *Gordonibacter* genus (Fig. S10A, S11A) consistent with previous reports[6, 7].

To obtain (*S*,*S*)-**5**, a *B. producta* DSM 3507 culture was inoculated directly from a glycerol stock into 20 mL of BHIrcf medium containing 100 μM (+)-matairesinol (BLD Pharmatech, BD298610) and incubated at 37 °C anaerobically for 120 h with > 90% substrate consumption. The production of (*S*,*S*)-**5** was confirmed using LC–MS/MS as described below. The spent medium after incubation was centrifuged at 3220× *g* for 15 min at 4 °C in a tabletop swinging bucket centrifuge, filtered through a 0.22 μm syringe filter (VWR) twice, and then diluted 2-fold or 4-fold into fresh BHIrcf medium. The diluted cell free supernatant (CFS) containing (*S*,*S*)-**4** was used to grow Coriobacteriia strains to test metabolism of (*S*,*S*)-**4**. The CFS containing (*S*,*S*)-**5** was further extracted using equal volume of ethyl acetate containing 1.5% formic acid three times. The organic layer was dried using anhydrous $MgSO_4$ and evaporated under reduced pressure to give a residue. The (*S*,*S*)-**5** residue was further lyophilized and resuspended in 1 mL of BHIrcf, which was used to measure metabolism. (*R*,*R*)-**5** was chemically synthesized from (–)-arctigenin (Biosynth, FA41645-0.5G) according to a previously published protocol[7] and dissolved in DMF to make a 20 mM stock solution. The stereochemistry of (*S*,*S*)-**5**, derived from (+)-matairesinol, was inferred based on the stereochemistry of (+)-matairesinol, and further supported by its metabolism only by *Gordonibacter* but not by *Eggerthella* (Fig. S14A), consistent with a previous report[6]. The stereochemistry of (*R*,*R*)-**5**, derived from (–)-arctigenin, was ascertained based on the previous reports that no modifications of compound stereocenters occur under the preparation condition[6, 7], and was further supported by the observed metabolism of the substrate only by *Eggerthella* but not *Gordonibacter* (Fig. S12B) consistent with previous reports[6].

To measure (*S*,*S*)-**4** and (*R*,*R*)-**5** metabolism, Coriobacteriia WT strains were grown to saturation in BHIrcf media, and the engineered *G. uro* strains were grown to saturation in BHIrcf with 100 µg/mL of kanamycin. The saturated WT cultures were then inoculated 1:25 or 1:50 either into 200 µL of diluted CFS containing (*S*,*S*)-**4** or 200 µL of BHIrcf supplemented with 100 µM (*R*,*R*)-**5** in triplicate in 96-well plates. For engineered *G. uro* strains, 100 µg/mL of kanamycin was added to the growth medium. The cultures were incubated at 37 °C anaerobically for 48–72 h.

To measure (*R*,*R*)-**4** metabolism, Coriobacteriia WT strains were grown to saturation in BHIrcf media, and the engineered *E. lenta* strains were grown to saturation in BHIrcf with 100 µg/mL of kanamycin. The saturated WT cultures were then inoculated 1:20 into 200 µL of BHIf supplemented with 50 µM (*R*,*R*)-**4** in triplicate in 96-well plates. The cultures were incubated at 37 °C anaerobically for 48 h. To test the activity of engineered *E. lenta* strains, the saturated cultures were inoculated 1:30 into 30 mL of BHIrcf medium supplemented with 100 µg/mL kanamycin and 100 µM cumate to induce protein expression and incubated at 37 °C anaerobically for 20–24 h. After overnight induction, the cultures were then centrifuged (3220× *g*, 10 min, 4 °C), and the supernatants were decanted inside of the anaerobic chamber. Cell pellets were resuspended in 2 mL of pre-reduced phosphate-buffered saline (PBS, pH 7.4). 190 µL of the cell suspension or PBS was incubated with 5 mM methyl viologen, 10 mM sodium dithionite, and 1 µL of 10 mM (*R*,*R*)-**4** in triplicate in a 96-well plate. Methyl viologen and sodium dithionite are used as artificial electron donors to allow for enzyme cofactor reduction. The plate was incubated at 37 °C anaerobically for 24 h.

To measure (*S*,*S*)-**5** metabolism, Coriobacteriia WT strains were grown to saturation in BHIrcf media, and the engineered *E. lenta* DSM 2243 strains were grown to saturation in BHIrcf with 100 µg/mL of kanamycin. The saturated WT cultures were then inoculated 1:20 into 100 µL of BHIf supplemented with 5 µL of the aforementioned (*S*,*S*)-**5**-BHIrcf solution in triplicate in 96-well plates. The cultures were incubated at 37 °C anaerobically for 48–72 h. To test the activity of engineered *E. lenta* strains, the saturated cultures were inoculated 1:30 into 15 mL of BHIrcf medium supplemented with 100 µg/mL kanamycin and 100 µM cumate to induce protein expression and incubated at 37 °C anaerobically for 20–24 h. After overnight induction, the cultures were then centrifuged (3220× *g*, 10 min, 4 °C), and the supernatants were decanted inside of the anaerobic chamber. Cell pellets were resuspended in 0.7 mL of pre-reduced PBS (pH 7.4). 100 µL of the cell suspension was incubated with 5 mM methyl viologen, 10 mM sodium dithionite, and 5 µL of the aforementioned (*S*,*S*)-**5**-BHIrcf solution in triplicate in a 96-well plate. The plate was incubated at 37 °C anaerobically for 72 h.

The plates were then centrifuged (3220× *g*, 10 min, 4 °C), and the supernatants were harvested. 40 µL of the supernatant was diluted 1:5 with 160 µL of LC–MS grade (Honeywell) methanol, and 100 µL of the resulting mixture was then diluted 1:2 with 100 µL of Milli-Q water. The amount of catechols and products in the diluted mixtures were quantified using UPLC–MS/MS.

UPLC–MS/MS was conducted using a Waters Acquity UPLC H-Class System (Waters Corporation), and Waters Xevo TQ-S (Waters Corporation) instrument. 5 or 10 μL of each sample was injected onto a CORTECS T3 Column (120Å, 2.7 μm, 2.1 mm X 100 mm, Waters Corporation). The flow rate was 0.5 mL/min using solvent A = 0.1% formic acid in H_2O and solvent B = 0.1% formic acid in acetonitrile (Honeywell). The column temperature was maintained at 40 °C. The following gradient was applied: 0–1 min at 100% A isocratic, 1.0–2.0 min at 0–90% B, 2.0–2.5 min at 90% B isocratic, 2.5–2.75 min at 90–0% B, 2.75–3.50 min at 0% B isocratic. MS detection was performed using electron spray ionization in negative mode (ESI–) (capillary voltage, 2.60 kV; cone voltage, 29 V; source offset voltage, 50 V; desolvation temperature, 200 °C; desolvation gas flow, 800 L/h; cone gas flow, 150 L/h; nebulizer, 7.0 bar).

For detecting metabolism of (*R*,*R*)-**4** and (*S*,*S*)-**4**, the masses of SECO (precursor ion *m*/*z* = 361.1651, daughter ion *m*/*z* = 136.0524; cone voltage 25 V; collision energy 30 V), **4** (precursor ion *m*/*z* = 333.1338, daughter ion *m*/*z* = 122.0367; cone voltage 25 V; collision energy 30 V), **9** (precursor ion *m*/*z* = 317.1389, daughter ion *m*/*z* = 122.0367; cone voltage 25 V; collision energy 30 V) and **10** (precursor ion *m*/*z* = 301.1439, daughter ion *m*/*z* = 106.0418; cone voltage 25 V; collision energy 30 V) were monitored, based on previous reports[6-9] with further optimization.

For detecting metabolism of (*R*,*R*)-**5** and (*S*,*S*)-**5**, the masses of **5** (precursor ion *m*/*z* = 329.1025, daughter ion *m*/*z* = 123.0440; cone voltage 25 V; collision energy 30 V), **11** (precursor ion *m*/*z* = 313.1076, daughter ion *m*/*z* = 191.0700; cone voltage 25 V; collision energy 20 V), **12** (precursor ion *m*/*z* = 313.1076, daughter ion *m*/*z* = 269.1170; cone voltage 25 V; collision energy 20 V) and **13** (precursor ion *m*/*z* = 297.1127, daughter ion *m*/*z* = 253.1220; cone voltage 25 V; collision energy 15 V) were monitored, based on previous reports[6-9] with further optimization. Daughter ion scanning was used to monitor fragmentation patterns of **11**/**12** (precursor ion *m*/*z* = 313.1076 in negative ion mode or *m*/*z* = 315.12 in positive ion mode) when applicable.

<u>Catechol dehydroxylase purification</u>

The engineered *G. uro* strains harboring plasmids encoding cumate-inducible catechol dehydroxylases (and any required accessory proteins) were first grown to saturation in BHIrcf medium with 100 μg/mL of kanamycin. The saturated cultures were then inoculated 1:25 into 1 L or 2 L of BHIrcf with 100 μg/mL of kanamycin and 100 μM cumate, and were incubated at 37 °C anaerobically for 20–24 h. Cell pellets were harvested by centrifuging at 6,000× *g* for 10 min at 4 °C, and the supernatants were decanted. All subsequent steps were performed anaerobically at 4 °C unless otherwise specified. All buffers were sparged with nitrogen and equilibrated in anaerobic chamber overnight before use. Cell pellets from 1 L or 2 L cultures were resuspended in 30 mL of lysis buffer (50 mM HEPES, 30 mM imidazole, 250 mM NaCl, 2 mg/mL lysozyme, 0.05 mg/mL DNase I, Pierce EDTA-free protease inhibitor (Thermo Fisher A32965), pH 8). Cells were lysed by sonication using a ½ inch horn at 35% amplitude for 6 or 10 min (5 sec on followed by 10

sec off) while being kept in an ice water bath. Lysate was centrifuged at 19,000× *g* for 45 min at 4 °C to separate soluble and insoluble fractions. A 3 mL bed volume of Ni-NTA resin was equilibrated with 30 mL of lysis buffer and was then mixed with soluble lysate followed by 1 h incubation with constant agitation. The lysate was then loaded onto a column by gravity flow. The column was washed twice with 15 mL of wash buffer (50 mM HEPES, 30 mM imidazole, 250 mM NaCl, pH 8). Protein was eluted from the column with 20 mL of elution buffer (50 mM HEPES, 250 mM imidazole, 250 mM NaCl, pH 8). The elution fraction was concentrated using an Ultra-15 Centrifugal Filter (30 kDa MWCO membrane, Amicon) and desalted using a disposable PD-10 desalting column (Cytiva) with desalting buffer (50 mM HEPES, 250 mM NaCl, 10% glycerol (v/v), pH 8). The desalted proteins were again concentrated using a new centrifugal concentrator (30 kDa MWCO). Protein concentrations were estimated by measuring the absorption at a wavelength of 280 nm using NanoDrop 2000 UV-Vis Spectrophotometer (Thermo Scientific). The protein samples were flash frozen and stored in liquid nitrogen until further use. The purity of purified proteins was assessed by SDS-PAGE after heat protein solutions in Laemmli Sample Buffer with 2-mercaptoethanol at 95 °C for 10 min.

Western blotting

Saturated cultures of *G. uro* strains encoding vector or Dadh plasmid variants were inoculated 1:20 in BHIrcf medium with 100 µg/mL kanamycin and 1 mM dopamine, and were incubated at 37 °C for 20 h. Cell pellets were then harvested by centrifugation (3220 × g, 10 min, 4 °C), and heated in Laemmli Sample Buffer with 2-mercaptoethanol at 95 °C for 20 min. Standard Western blotting analysis was performed using 10–20% SDS-PAGE gels with a primary Anti-His antibody HRP conjugate (1:20000, Novagen 71841-125 µL) incubation at 4 °C overnight, and imaged with CN-DAB substrate (Thermo Fisher 34000) using an Azure Imaging Systems (Azure Biosystems).

Measurement of *E. lenta* catechol dehydroxylase substrate specificity

The substrates (*S*)-**1**, (*R*)-**1**, (*S*,*S*)-**4**, (*R*,*R*)-**4**, (*S*,*S*)-**5** and (*R*,*R*)-**5** and were prepared as described above. **3** was dissolved in DMF to make a 20 mM stock solution. CFS containing (*S*)-**2** or (*R*)-**2** was prepared as described earlier with the exception that for the assays in Fig. S15A, GAMrcf medium (Gifu Anaerobic Broth with 1% w/v arginine, 0.05% w/v L-cysteine hydrochloride and 10 mM sodium formate), instead of GAM medium, was used to culture *F. plautii* DSM 4000 and *E. lenta* AB8n2 with 2 mM (+)-catechin or 2 mM (–)-epicatechin, and was further diluted in BHIrcf instead of BHIf.

Coriobacteriia WT strains were grown to saturation in BHIrcf medium, and the engineered *G. uro* strains were grown to saturation in BHIrcf with 100 µg/mL of kanamycin. The saturated WT cultures were then inoculated 1:25 either into 200 µL of BHIrcf supplemented with 100 µM **3** or 100 µM (*R*,*R*)-**5**, or into 200 µL of diluted CFS containing (*R*)-**1**, (*S*)-**1**, (*S*)-**2** or (*S*,*S*)-**4** in triplicate in 96-well plates. For engineered *G. uro* strains, 100 µg/mL of kanamycin was added to the growth medium.

The cultures were incubated at 37 °C anaerobically for 60 h. The cultures after incubation were diluted as described earlier and the amount of catechols and products in the diluted mixtures were quantified using UPLC–MS/MS as described earlier.

To characterize the in vitro substrate specificity of catechol dehydroxylases, 100–200 nM purified enzymes were incubated with 5 mM methyl viologen, 10 mM sodium dithionite and corresponding substrates (approximately 200 μM **2**, 200 μM **3**, 50 μM **4**, and 20 μM **5**) in 100 μL pre-reduced PBS (pH 7.4) in triplicate in a 96-well plate. The plate was incubated at 37 °C anaerobically for 24 or 48h. The reaction mixtures after incubation were diluted as described earlier and the amount of catechols and products in the diluted mixtures were quantified using UPLC–MS/MS as described earlier.

Culturing *E. lenta* and *G. pamelaeae* with catechols, RNA extraction and RT-qPCR experiments

For identification of genes induced by (–)-epicatechin, a saturated culture of *E. lenta* DSM 2243 was inoculated into BHIrcf medium and grown to OD_{600} of 0.3–0.5 (mid-log phase). 1.5 mL of the culture was exposed to either 1 mM (–)-epicatechin (200 mM in DMF) or the same volume of vehicle (DMF) in triplicate. Then cultures were incubated anaerobically at 37 °C for 3–4 h. For identification of genes induced by (*S*,*S*)-**4**, (*S*,*S*)-**4** was first extracted from 8 mL of 250 μM (+)-SECO/*B. producta* CFS using the same approach described for (*S*,*S*)-**5** extraction and dissolved in 0.5 mL of BHIrcf medium. 100 μL of (*S*,*S*)-**4**-BHIrcf solution was added to 1.5 mL of *E. lenta* DSM 2243 mid-log phase cultures in triplicate. For identification of genes induced by (*R*,*R*)-**5**, 1.5 mL of *E. lenta* DSM 2243 mid-log phase cultures were exposed to 100 μM (*R*,*R*)-**5** (20 mM in DMF) in triplicate. DMF was used as a vehicle control. For identification of genes induced by (*S*,*S*)-**5**, a saturated culture of *G. pamelaeae* DSM 19378 was inoculated into BHIrcf medium and grown to OD_{600} of 0.15–0.2 (mid-log phase), and 100 μL of the aforementioned (*S*,*S*)-**5**-BHIrcf solution or 100 μL of BHIrcf medium was added to 1.5 mL of *G. pamelaeae* DSM 19378 cultures in triplicate. Then cultures were incubated anaerobically at 37 °C for 3–4 h. For identification of *Gordonibacter* sp. 28C genes induced by **3**, a saturated culture of *Gordonibacter* sp. 28C was inoculated into BHIf medium and grown to an OD_{600} of 0.1–0.12 (mid-log phase). 0.75 mL of the culture was first diluted with 0.75 mL of fresh BHIf medium and exposed to either 100 μM **3** (20 mM in DMF) or the same volume of vehicle (DMF) in triplicate. Then cultures were incubated anaerobically at 37 °C for 5 h. Cell pellets were then harvested by centrifugation (3220× *g*, 10 min, 4 °C), resuspended in 800 μL of TRIzol reagent (Invitrogen, catalog number 15596-026), and used for RNA extraction immediately or stored in a freezer at –80 °C until RNA extraction.

Total RNA was isolated first by bead beating the TRIzol cell suspensions for 2.5 min twice to lyse cells using Mini-Beadbeater-16 (BioSpec) and ZR BashingBead Lysis Tubes (0.1 & 0.5 mm) (Zymo Research, S6012-50), and then using the Zymo Research Direct-Zol RNA MiniPrep Plus kit (R2070)

according to a previously published protocol[1]. From total RNA, complementary DNA synthesis and PCR amplification were performed using Luna Universal One-Step RT-qPCR Kit according to the manufacturer's protocol (NEB, E3005S). Assays were performed using a Bio-Rad CFX Opus Real-Time PCR System. The primers used for amplification are listed in Supplementary Table 2. Fold changes in transcript levels were calculated using the $\Delta\Delta C_T$ method normalized to one replicate in the vehicle controls.

Phylogenetic analysis of Coriobacteriia genomes and catechol dehydroxylases

Phylogenetic analysis of Coriobacteriia genomes was performed using PhyloPhlAn v3.0[10] for 113 representative Coriobacteriia genomes retrieved from NCBI (genome accession: Supplementary Table 3). The *Bifidobacterium animalis* subsp. Lactis BLC1 genome was included as an outgroup. The distribution of catechol dehydroxylases of interest in different Coriobacteriia genomes was analyzed by BLAST searches against a local database consisting of the 113 Coriobacteriia genomes using Geneious Prime 2023. BLAST hits with coverage of higher than 85% and amino acid identity of over 80% for *Gs* Vadh or 75% for other enzyme queries were considered enzyme homologs. The Coriobacteriia phylogenetic tree and catechol dehydroxylase distribution were visualized using ggtree[11]. The catechol dehydroxylase gene clusters across different *Eggerthella* strains were compared with a minimum alignment sequence identity of 0.75 and visualized using clinker[12].

Among proteins containing a PF00384 domain (molybdopterin oxidoreductase), protein sequences encoded by Coriobacteriia were retrieved from UniProt. The sequences were then grouped by sequence similarity into a sequence similarity network (SSN) using EFI-EST[13]. At minimum alignment score of 130, all the biochemically characterized *Eggerthella*-type catechol dehydroxylases formed a single cluster, and all the biochemically characterized *Gordonibacter*-type catechol dehydroxylases formed a separate cluster. The sequences (total 419 sequences, Supplementary Table 4) in these two clusters were regarded as catechol dehydroxylase homologs. To infer a maximum-likelihood phylogenetic tree, we first grouped the sequences by 95% amino acid identity, resulting in 215 unique sequences. These sequences were then aligned using mafft-linsi (v7.505)[14] and then trimmed using trimal (v1.4.1, -gappyout)[15]. A maximum-likelihood phylogenetic tree was generated using iqtree2 (v2.1.3, 1000 ultrafast bootstraps)[16]. Other biochemically characterized members of DMSO reductase superfamily, acetylene hydratase (Ach, Q71EW5), DMSO reductase (DorA, Q52675), ethylbenzene dehydrogenase (EbdA, Q5P5I0) and pyrogallol hydroxytransferase (AthL, P80563) were used as outgroups for generating the phylogenetic tree. For better visualization, Ach was omitted from the tree. The tree was visualized using a free web-based tool, iTOL v6.5.8[17]. The amino acid identities of the characterized catechol dehydroxylases are listed in Supplementary Table 5.

Protein structural analysis

The prediction of protein complex formation between catechol dehydroxylases and their accessory proteins was performed using AlphaFold3[18]. The structural comparison between complementary enantioselective (Cadh/eCadh) and site-selective enzymes ((+)-Eldh1/(+)-Eldh2) was performed by PyMol (v3.0.0) using the predicted structures retrieved from UniProt. The position of molybdenum cofactor in these enzymes was estimated by overlaying the structure of 8J83 (PDB) on Cadh/eCadh, and 4DMR (PDB) on (+)-Eldh1/(+)-Eldh2. Foldseek[19] was used to identify proteins with similar structures to DadhG and DadhH.

Quantification and statistical analysis

Unless otherwise specified, statistical analysis was carried out using Prism 10 (GraphPad Software). Individual datapoints have been shown where possible but are otherwise represented as the mean ± standard deviation unless otherwise stated.

Supplementary Figures

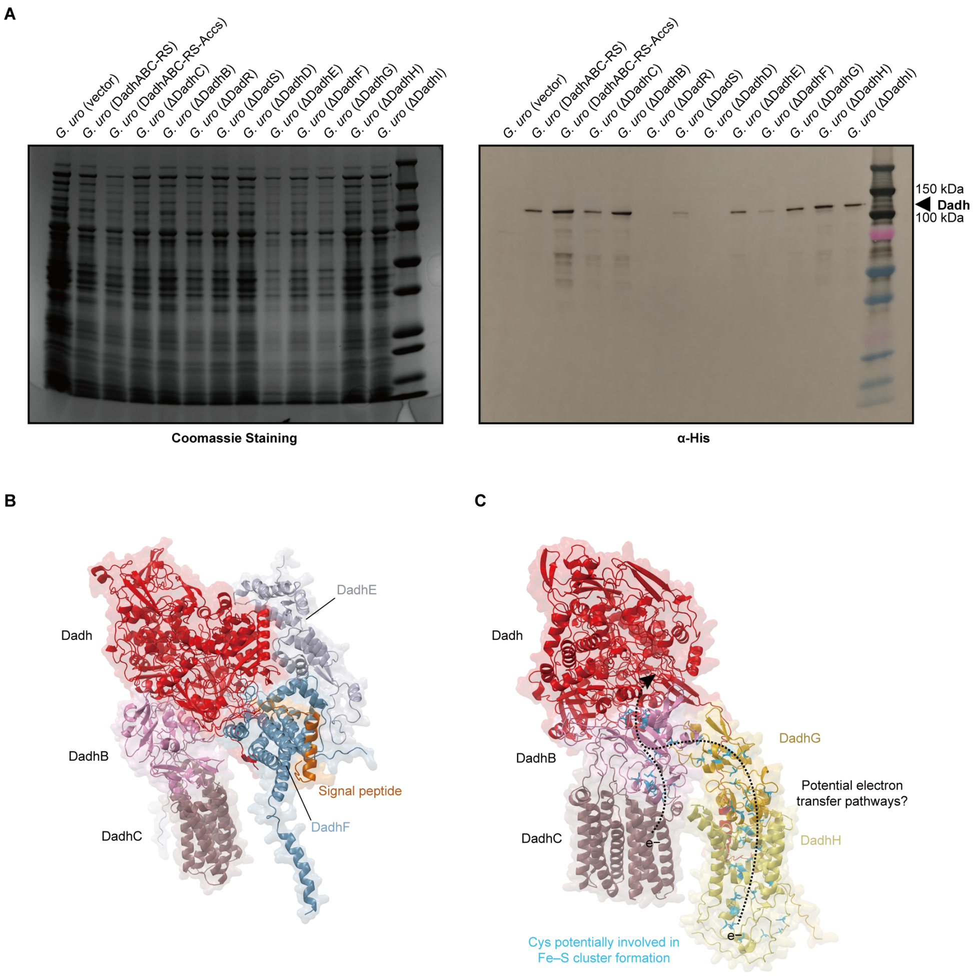

Figure S1. Heterologous expression of Dadh in *G. uro* and putative roles of Dadh accessory genes. A. Coomassie staining and anti-His western blot of *G. uro* strains harboring an empty vector or different His-tagged Dadh-encoding plasmids to assess Dadh expression. **B.** A predicted protein complex formed by DadhABC, DadhE, and DadhF. **C.** A predicted protein complex formed by DadhABC, DadhG and DadhH.

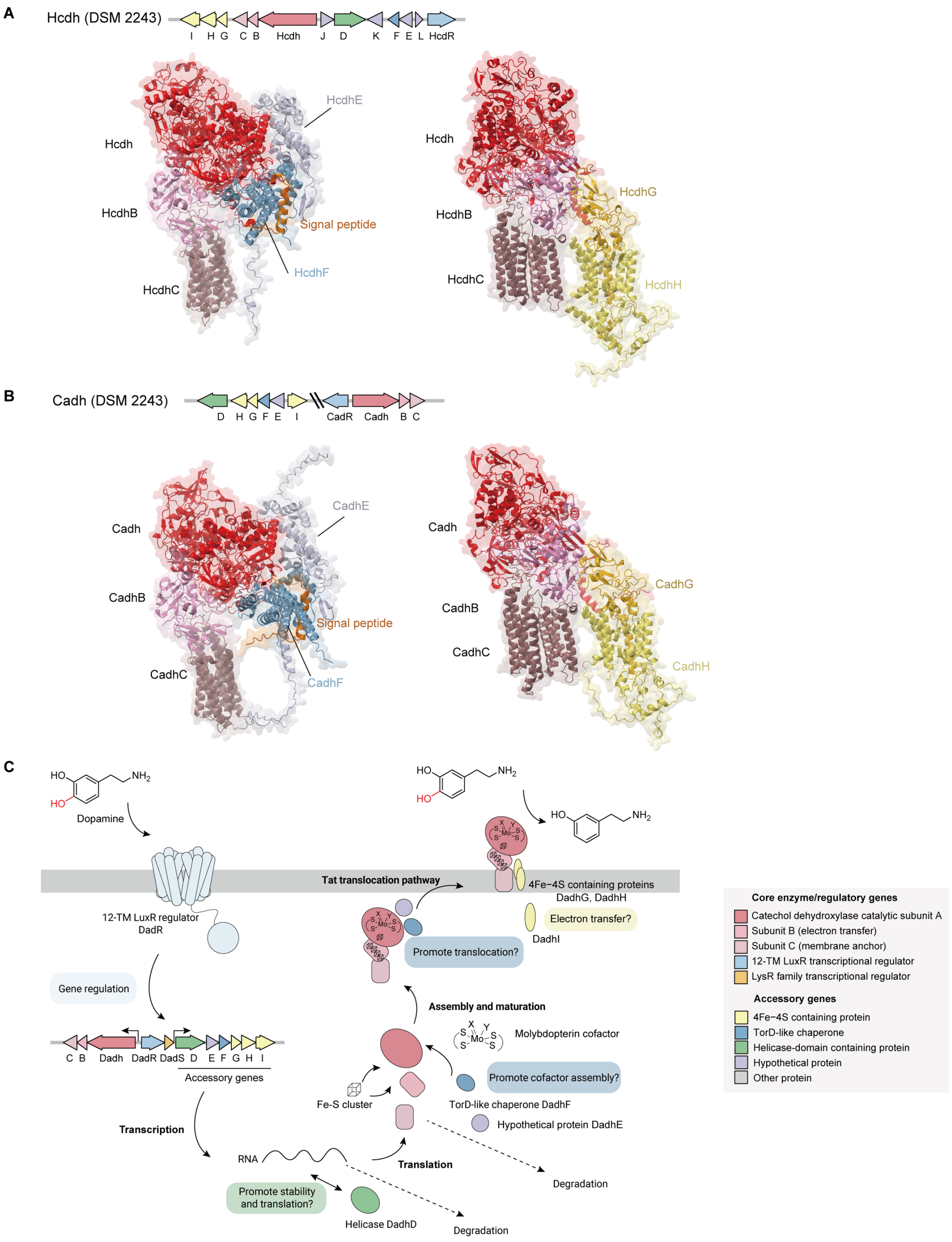

Figure S2. Putative roles of accessory genes in *Eggerthella* catechol dehydroxylases. A. Predicted protein complex formed by Hcdh and its accessory genes. **B.** Predicted protein complex formed by Cadh and its accessory genes. **C.** Proposed roles of accessory proteins in *Eggerthella* catechol dehydroxylase biogenesis and catalysis.

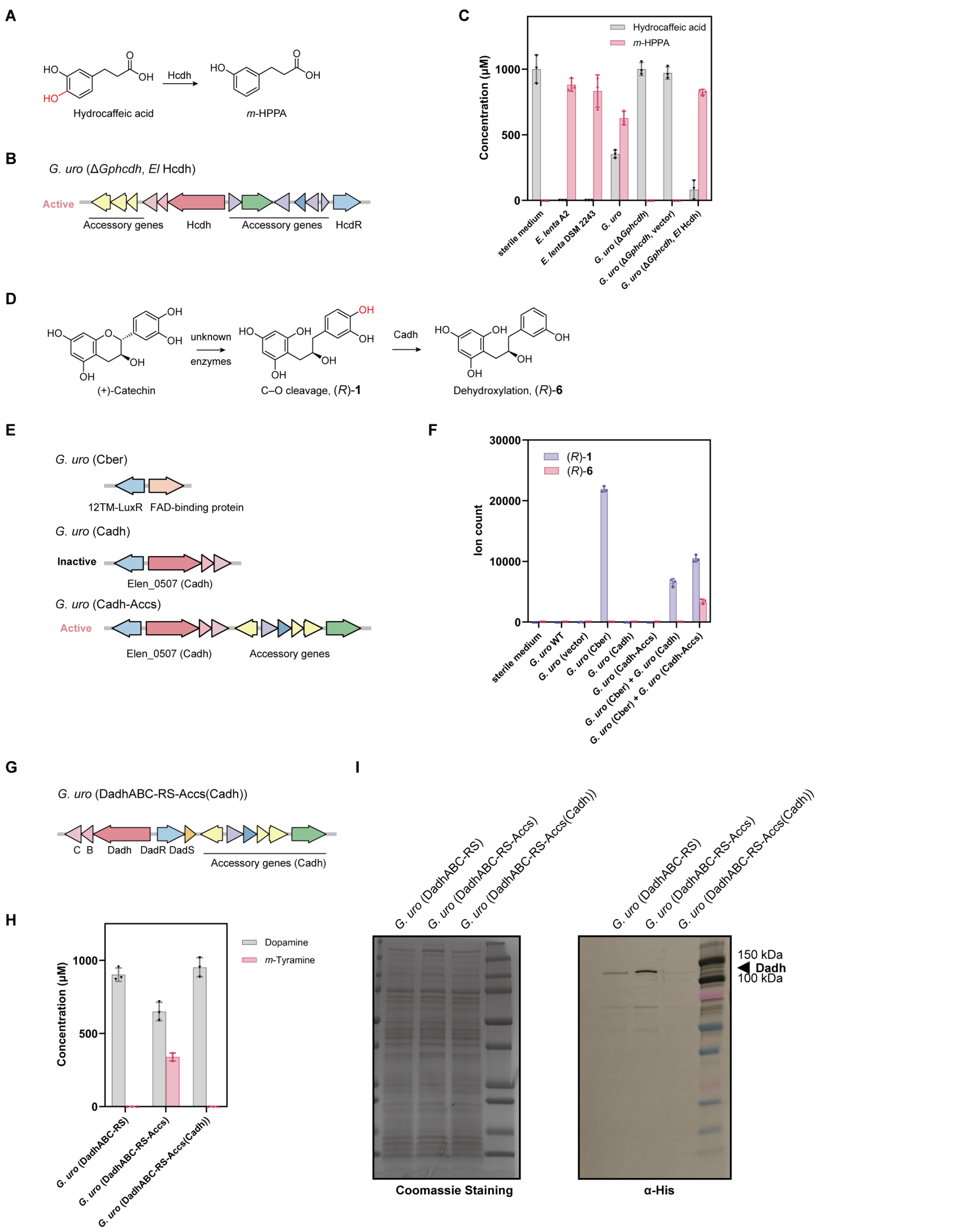

Figure S3. Heterologous expression of Hcdh and Cadh in *G. uro* and specificity of accessory genes. A. Hydrocaffeic acid dehydroxylation by Hcdh. **B.** Schematic of construct design for the engineered *G. uro* strain *G. uro* (*ΔGphcdh*, *El* Hcdh). **C.** LC–MS/MS data quantifying the production of dehydroxylated metabolite *m*-HPPA after incubation of hydrocaffeic acid with corresponding *E. lenta* WT, *G. uro* WT, and engineered *G. uro* strains. **D.** (+)-Catechin metabolism by *E. lenta*. **E.** Schematic of construct design for the engineered *G. uro* strains *G. uro* (Cber), *G. uro* (Cadh), and *G. uro* (Cadh-Accs). **F.** LC–MS/MS data quantifying the production of metabolites (*R*)-**1** and (*R*)-**6** after incubation of (+)-catechin with corresponding *G. uro* WT and engineered *G. uro* strains. **G.** Schematic of construct design for the engineered *G. uro* strain *G. uro* (DadhABC-RS-Accs(Cadh)) which encodes Dadh core subunits and regulators, and the accessory genes from the Cadh gene cluster. **H.** LC–MS/MS data quantifying the production of *m*-tyramine after incubation of dopamine

with corresponding engineered *G. uro* strains. **I.** Coomassie staining and anti-His western blot of *G. uro* strains harboring different His-tagged Dadh-encoding plasmids to assess Dadh expression. Data represented as mean ± SD with n = 3 biological replicates in **C**, **F**, and **H**.

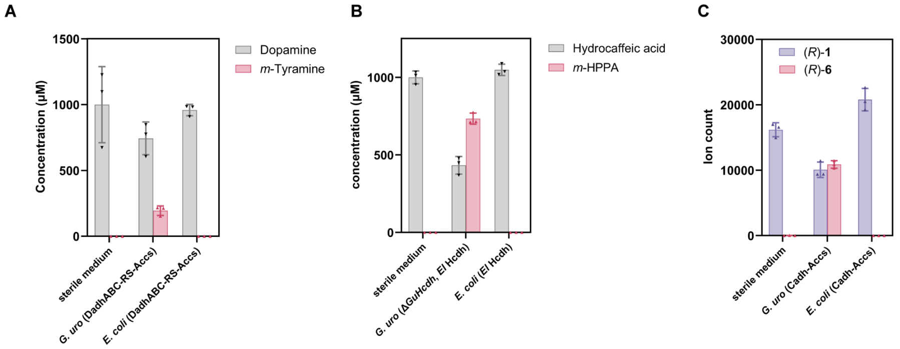

Figure S4. Heterologous expression of Dadh, Hcdh and Cadh in *E. coli*. A. LC–MS/MS data quantifying the production of *m*-tyramine after incubation of dopamine with engineered *G. uro* strain and *E. coli* strain harboring the same Dadh-encoding plasmid. **B.** LC–MS/MS data quantifying the production of dehydroxylated metabolite *m*-HPPA after incubation of hydrocaffeic acid with engineered *G. uro* strain and *E. coli* strain harboring the same Hcdh-encoding plasmid. **C.** LC–MS/MS data quantifying the production of dehydroxylated metabolite (*R*)-**6** after incubation of (*R*)-**1** with engineered *G. uro* strain and *E. coli* strain harboring the same Cadh-encoding plasmid. Data represented as mean ± SD with n = 3 biological replicates in **A**–**C**.

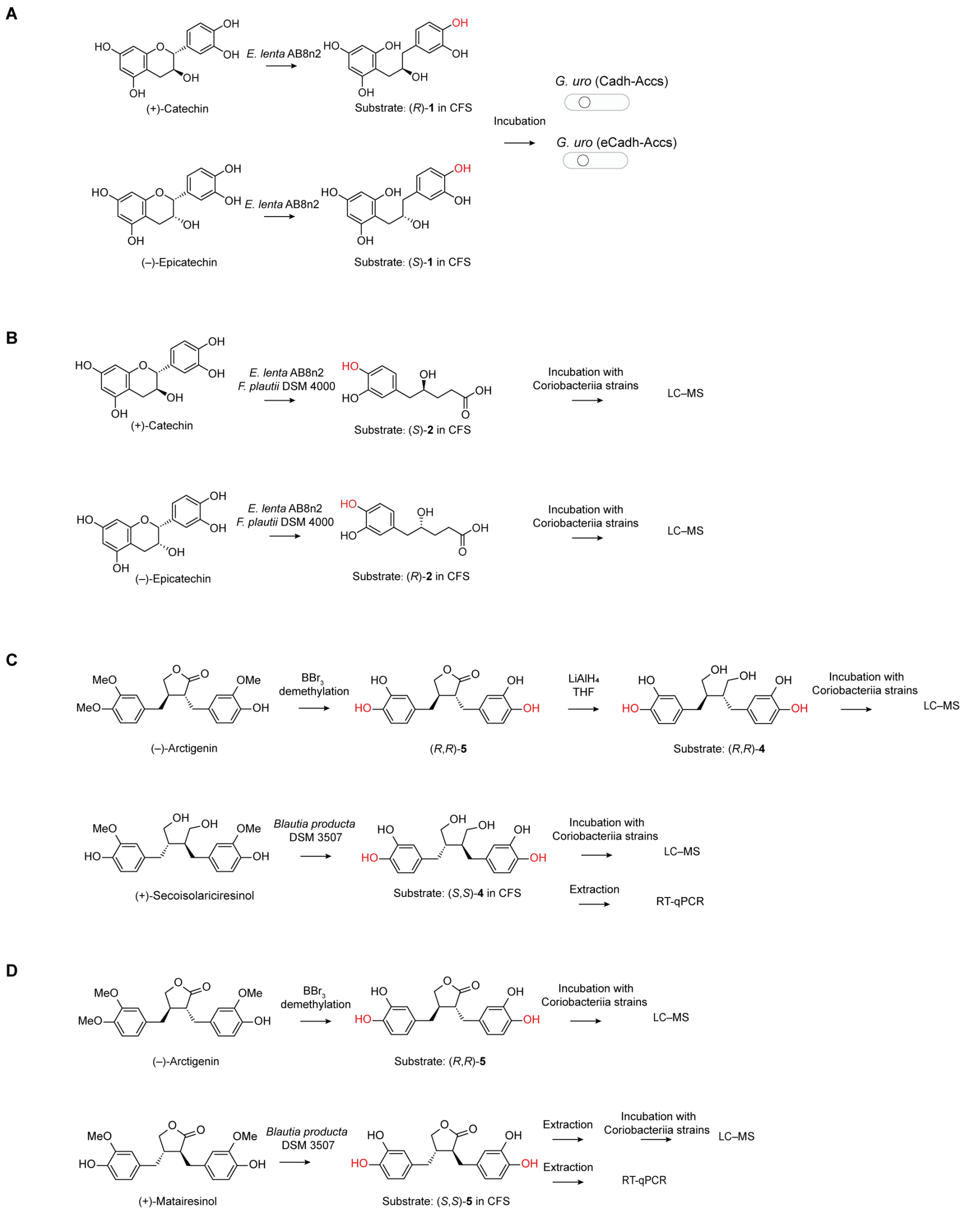

Figure S5. Preparation of catechol dehydroxylase substrates. A. *E. lenta* AB8n2 was incubated with (+)-catechin or (–)-epicatechin to generate (*R*)-**1** or (*S*)-**1** to test the substrate specificity of Cadh and eCadh. **B.** *E. lenta* AB8n2 and *F. plautii* DSM 4000 were incubated with (+)-catechin or (–)-epicatechin to generate (*S*)-**2** or (*R*)-**2**. **C.** (–)-Arctigenin was first demethylated using boron tribromide to produce (*R*,*R*)-**5**, which is further reduced by lithium aluminum hydride to (*R*,*R*)-**4**. *B. producta* DSM 3507 was incubated with (+)-SECO to generate (*S*,*S*)-**4**. **D.** (–)-Arctigenin was demethylated using boron tribromide to produce (*R*,*R*)-**5**. *B. producta* DSM 3507 was incubated with (+)-matairesinol to produce (*S*,*S*)-**5**.

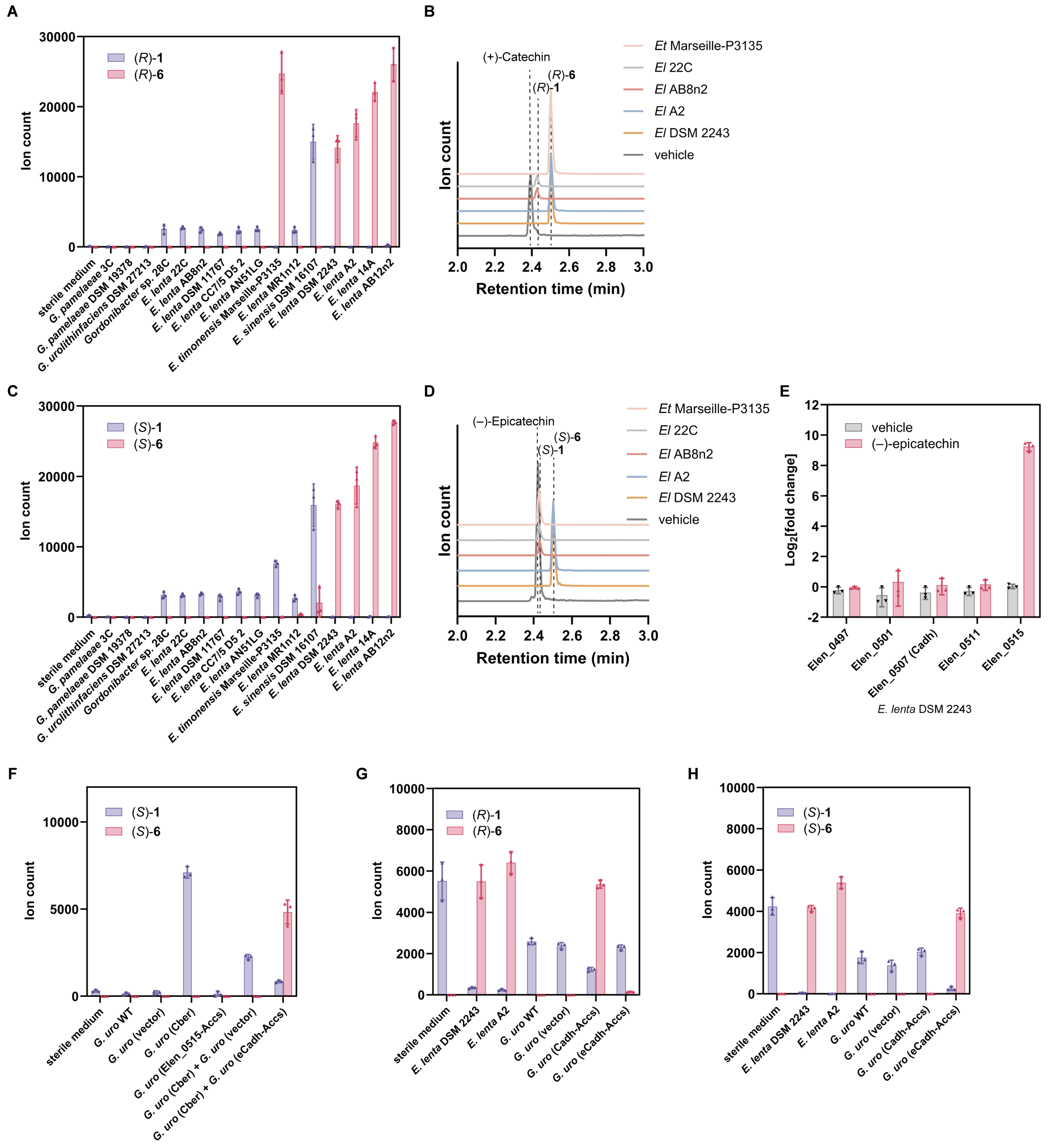

Figure S6. Metabolism of (+)-catechin and (–)-epicatechin by different Coriobacteriia WT strains, and identification and characterization of eCadh. A,**B.** LC–MS/MS quantification (**A**) and representative chromatogram traces (**B**) of metabolites (*R*)-**1** and (*R*)-**6** after incubation of (+)-catechin with different Coriobacteriia strains. **C**,**D.** LC–MS/MS quantification (**C**) and representative chromatogram traces (**D**) of metabolites (*S*)-**1** and (*S*)-**6** after incubation of (–)-epicatechin with different Coriobacteriia strains. **E.** RT-qPCR to test the expression level changes of uncharacterized catechol dehydroxylases and *cadh* upon exposure to (–)-epicatechin in *E. lenta* DSM 2243. **F.** LC–MS/MS data quantifying the production of metabolites (*S*)-**1** and (*S*)-**6** after incubation of (–)-epicatechin with corresponding *G. uro* WT and engineered *G. uro* strains to confirm the activity of eCadh for (*S*)-**1** dehydroxylation. **G**,**H.** LC–MS/MS data quantifying the production of (*R*)-**1** dehydroxylated metabolite (*R*)-**6** (**G**) and (*S*)-**1** dehydroxylated metabolite (*S*)-**6** (**H**) after incubation with corresponding *G. uro* WT and engineered *G. uro* strains, corresponding to Fig. 3E. Data represented as mean ± SD with n = 3 biological replicates in **A**, **C**, and **F**–**H**. Data represented as mean ± S.E.M. with n = 3 biological replicates in **E**.

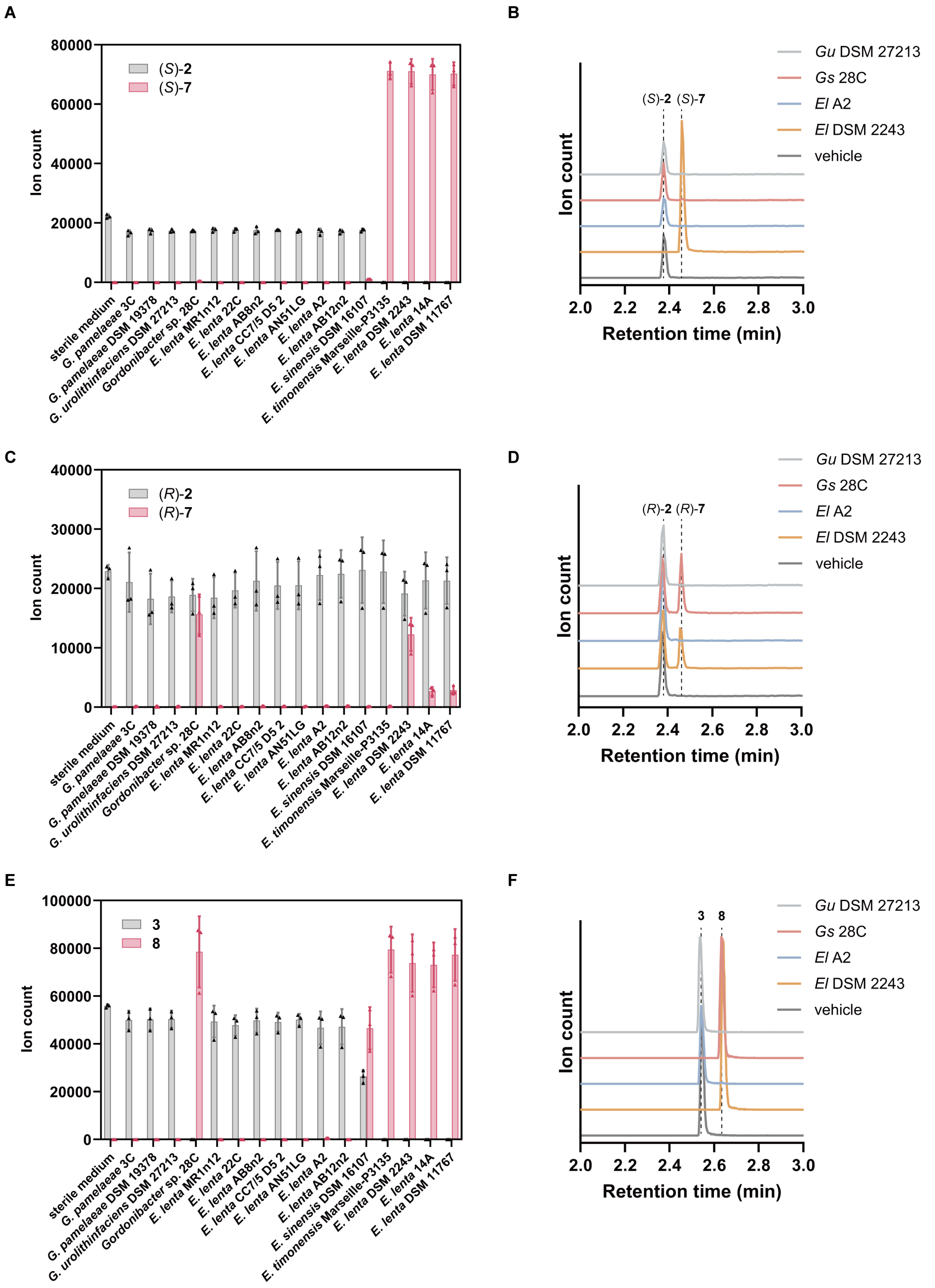

Figure S7. Metabolism of (*S*)-2, (*R*)-2 and 3 by different Coriobacteriia WT strains. A,**B.** LC–MS/MS quantification (**A**) and representative chromatogram traces (**B**) of the production of dehydroxylated metabolite (*S*)-**7** after incubation of (*S*)-**2** with different Coriobacteriia strains. **C**,**D.** LC–MS/MS quantification (**C**) and representative chromatogram traces (**D**) of the production of dehydroxylated metabolite (*R*)-**7** after incubation of (*R*)-**2** with different Coriobacteriia strains. **E**,**F.** LC–MS/MS quantification (**E**) and representative chromatogram traces (**F**) of the production of dehydroxylated metabolite **8** after incubation of **3** with different Coriobacteriia strains. Data represented as mean ± SD with n = 3 biological replicates in **A**, **C**, and **E**.

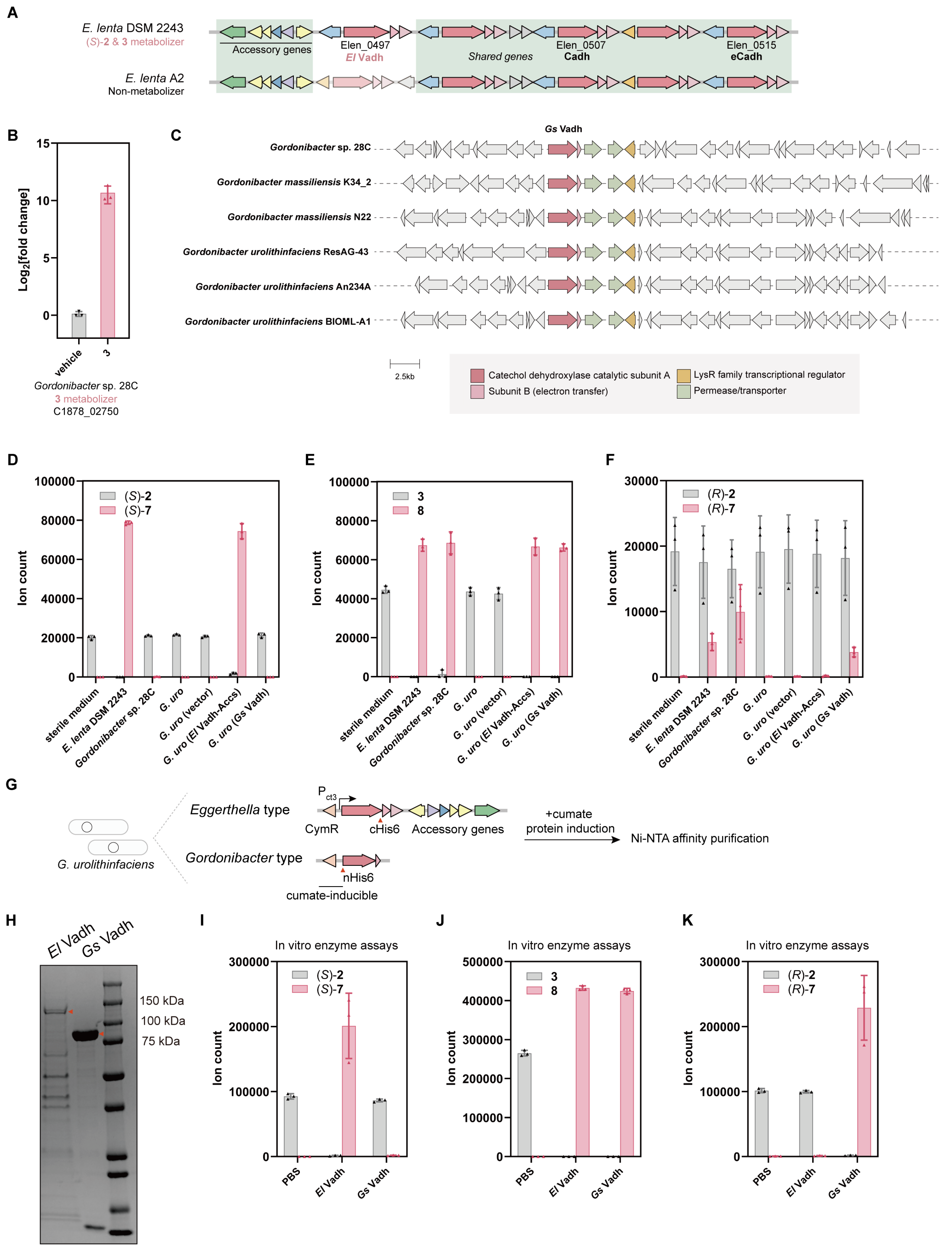

Figure S8. Identification and characterization of (*S*)-2, (*R*)-2, and 3 dehydroxylases. A. Comparing catechol dehydroxylases encoded in the (*S*)-**2** and **3** metabolizer *E. lenta* DSM 2243 and non-metabolizer *E. lenta* A2 suggests that Elen_0497 unique to *E. lenta* DSM 2243 encodes an (*S*)-**2** and **3** dehydroxylase (*El* Vadh). **B.** RT-qPCR revealed *Gordonibacter* sp. 28C gene C1878_02750 encoding an uncharacterized catechol dehydroxylase *Gs* Vadh was highly induced by **3**. **C.** Putative *Gs* Vadh gene cluster identified from comparison of multiple *Gordonibacter* strains encoding *Gs* Vadh. **D–F.** LC–MS/MS data quantifying the dehydroxylated metabolites (*S*)-**7** (**D**), **8** (**E**), and (*R*)-**7** (**F**) after incubation of (*S*)-**2** (**D**), **3** (**E**), and (*R*)-**2** (**F**) with corresponding WT Coriobacteriia and engineered *G. uro* strains, corresponding to Fig. 3H. **G.** Schematic of construct design for the engineered *G. uro* strains to express cumate-inducible His-tagged catechol

dehydroxylases for Ni-NTA protein affinity purification. **H.** SDS-PAGE of purified *El* Vadh and *Gs* Vadh proteins. **I**–**K.** LC–MS/MS data quantifying the dehydroxylated metabolites (*S*)-**7** (**I**), **8** (**J**), and (*R*)-**7** (**K**) after incubation of (*S*)-**2** (**I**), **3** (**J**), and (*R*)-**2** (**K**) with purified *El* Vadh and *Gs* Vadh proteins, corresponding to Fig. 3I. Data represented as mean ± S.E.M. with n = 3 biological replicates in **B**. Data represented as mean ± SD with n = 3 biological replicates in **D**–**F**, and **I**–**K**.

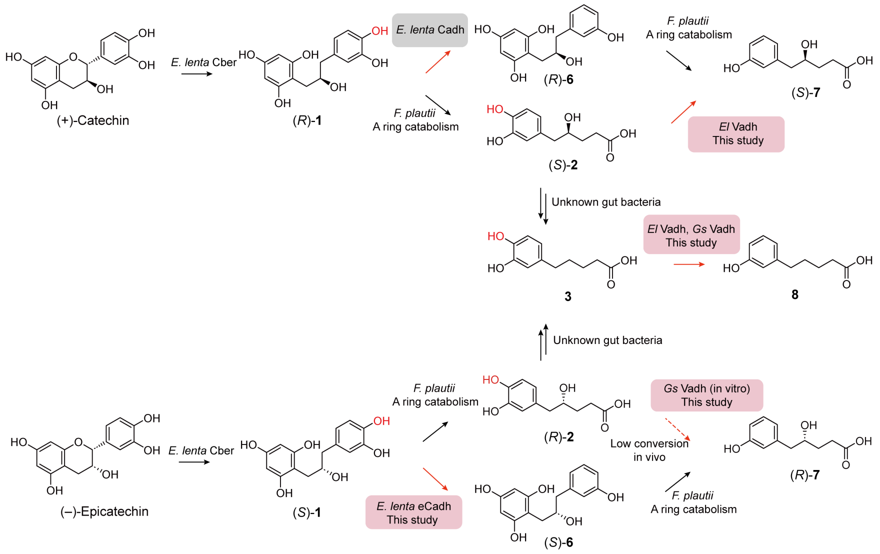

Figure S9. Summary of the characterized gut bacterial metabolism of (+)-catechin and (–)-epicatechin. Catechol dehydroxylases Cadh, eCadh and *El* Vadh from *Eggerthella* species and *Gs* Vadh from *Gordonibacter* species are involved in the gut bacterial metabolism of (+)-catechin and (–)-epicatechin. A previously characterized dehydroxylase Cadh is highlighted in gray, and catechol dehydroxylases identified in this study are highlighted in pink.

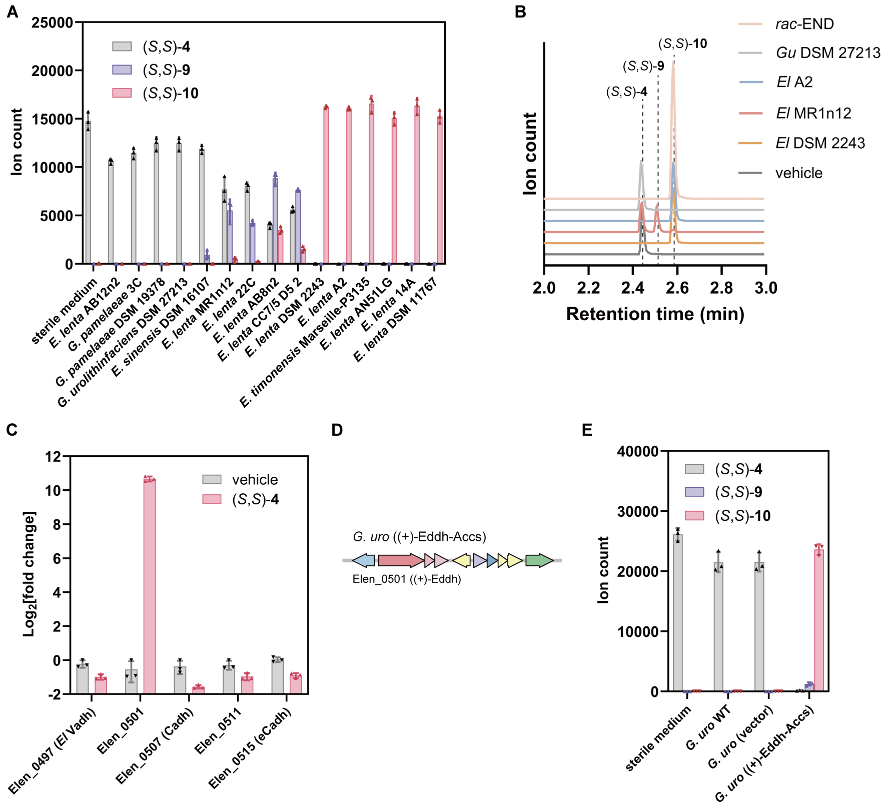

Figure S10. Metabolism of (*S*,*S*)-4 by different Coriobacteriia WT strains, and identification and characterization of (+)-Eddh. A,**B.** LC–MS/MS quantification (**A**) and representative chromatogram traces (**B**) of the production of dehydroxylated metabolites (*S*,*S*)-**9** and (*S*,*S*)-**10** after incubation of (*S*,*S*)-**4** with different Coriobacteriia strains. **C.** RT-qPCR to test the expression level changes of co-clustered catechol dehydroxylases in *E. lenta* DSM 2243 on exposure to (*S*,*S*)-**4** from CFS extract. **D.** Schematic of the construct design for the engineered *G. uro* strain *G. uro* ((+)-Eddh-Accs). **E.** LC–MS/MS data quantifying the production of (*S*,*S*)-**4** dehydroxylated metabolites (*S*,*S*)-**9** and (*S*,*S*)-**10** after incubation with *G. uro* WT and engineered *G. uro* strains to confirm activity of (+)-Eddh for (*S*,*S*)-**4** dehydroxylation. Data represented as mean ± SD with n = 3 biological replicates in **A** and **E**. Data represented as mean ± S.E.M. with n = 3 biological replicates in **C**.

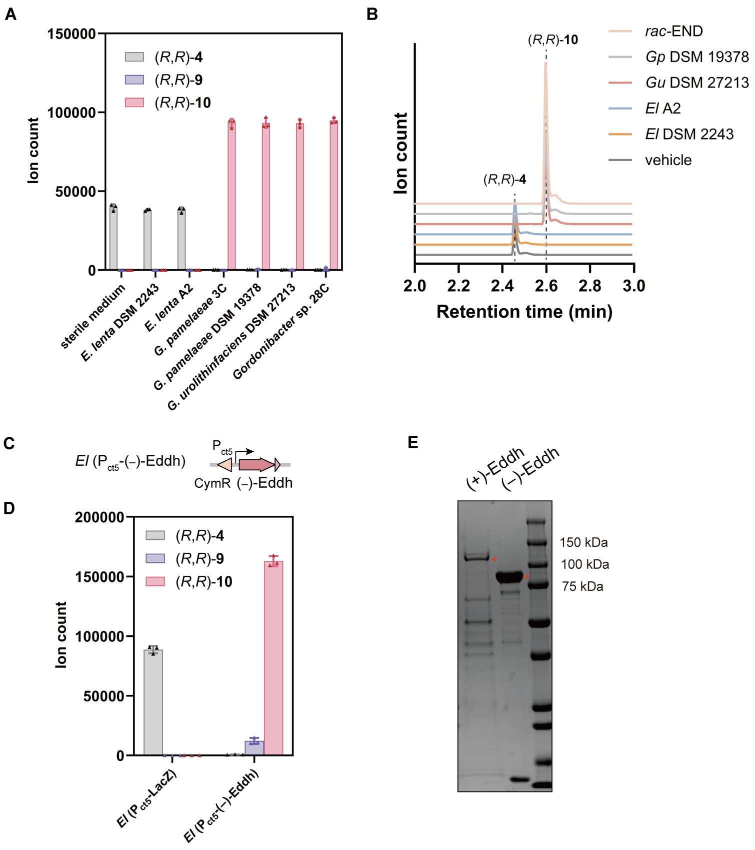

Figure S11. Metabolism of (*R*,*R*)-4 by different Coriobacteriia WT strains, and characterization of (–)-Eddh. A,**B.** LC–MS/MS quantification (**A**) and representative chromatogram traces (**B**) of the production of dehydroxylated metabolites (*R*,*R*)-**9** and (*R*,*R*)-**10** after incubation of (*R*,*R*)-**4** with different Coriobacteriia strains. **C.** Schematic of the construct design for the engineered *E. lenta* DSM 2243 strain *E. lenta* (P_{ct5}-(–)-Eddh). **D.** LC–MS/MS data quantifying the production of (*R*,*R*)-**4** dehydroxylated metabolites (*R*,*R*)-**9** and (*R*,*R*)-**10** after incubation with the cell resuspensions of engineered *E. lenta* strains in PBS, supplemented with methyl viologen and sodium dithionite, to confirm activity of (–)-Eddh for (*R*,*R*)-**4** dehydroxylation. **E.** SDS-PAGE of purified (+)-Eddh and (–)-Eddh proteins. Data represented as mean ± SD with n = 3 biological replicates in **A** and **D**.

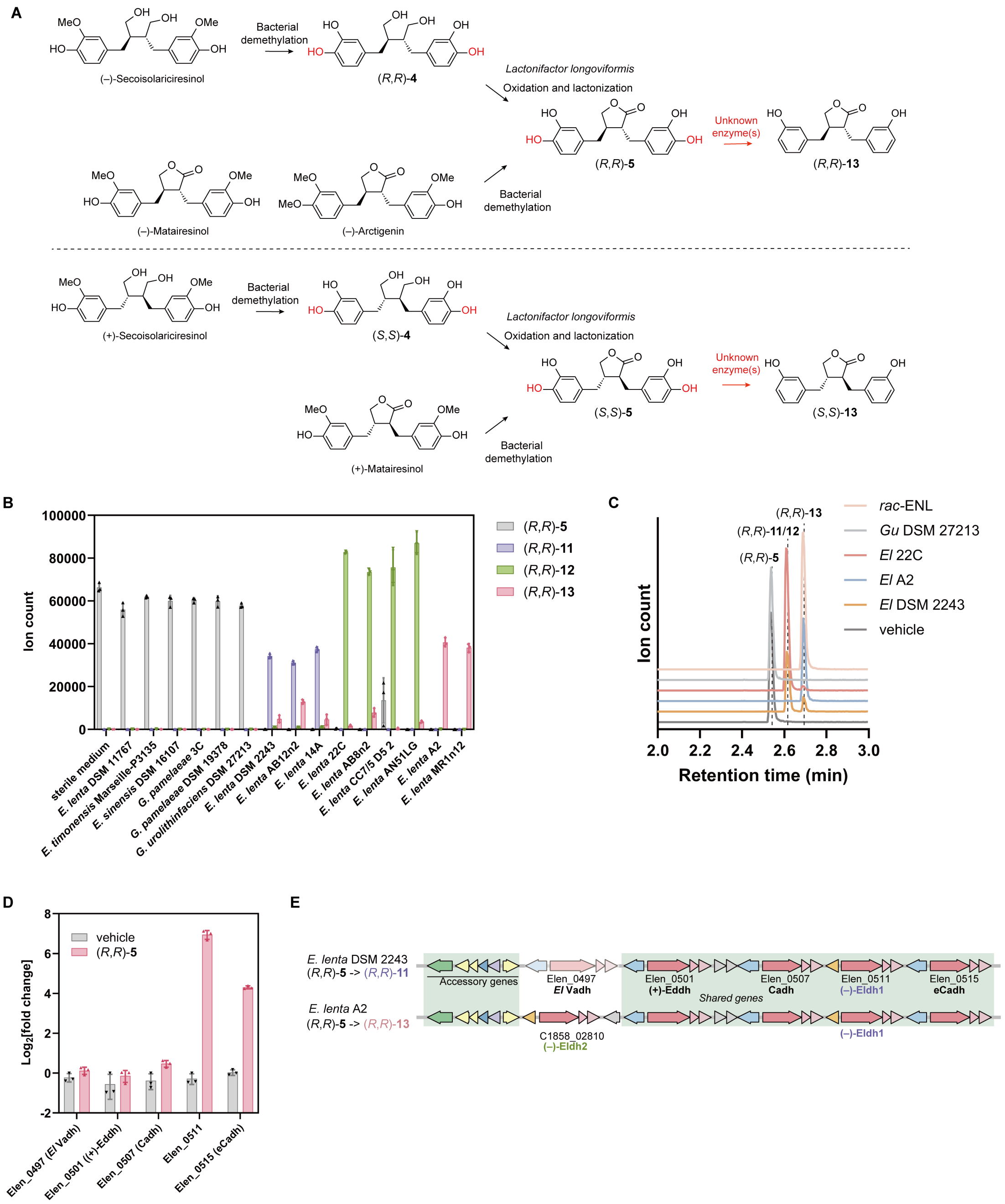

Figure S12. Gut bacterial metabolism related to 5, metabolism of (*R*,*R*)-5 by different Coriobacteriia WT strains, and identification of (–)-Eldh1 and (–)-Eldh2. A. Gut bacterial metabolism of (–)-secoisolariciresinol, (–)-matairesinol and (–)-arctigenin produces (*R*,*R*)-**5**, which can be further metabolized to (*R*,*R*)-**13** by unknown enzymes. Gut bacterial metabolism of (+)-secoisolariciresinol and (+)-matairesinol produces (*S*,*S*)-**5**, which can be further metabolized to (*S*,*S*)-**13** by unknown enzymes. **B**,**C.** LC–MS/MS quantification (**B**) and representative chromatogram traces (**C**) of the production of dehydroxylated metabolites (*R*,*R*)-**11**, (*R*,*R*)-**12**, and (*R*,*R*)-**13** after incubation of (*R*,*R*)-**5** with different Coriobacteriia strains. **D.** RT-qPCR to test the expression level changes of co-clustered catechol dehydroxylases in *E. lenta* DSM 2243 on exposure to (*R*,*R*)-**5** in *E. lenta* DSM 2243. **E.** Comparing catechol dehydroxylases encoded in the

(*R*,*R*)-**11**-producer *E. lenta* DSM 2243 and (*R*,*R*)-**13**-producer *E. lenta* A2 suggests that C1858_02810 unique to *E. lenta* A2 is responsible for the second dehydroxylation event on (*R*,*R*)-**5**. Data represented as mean ± SD with n = 3 biological replicates in **B**. Data represented as mean ± S.E.M. with n = 3 biological replicates in **D**.

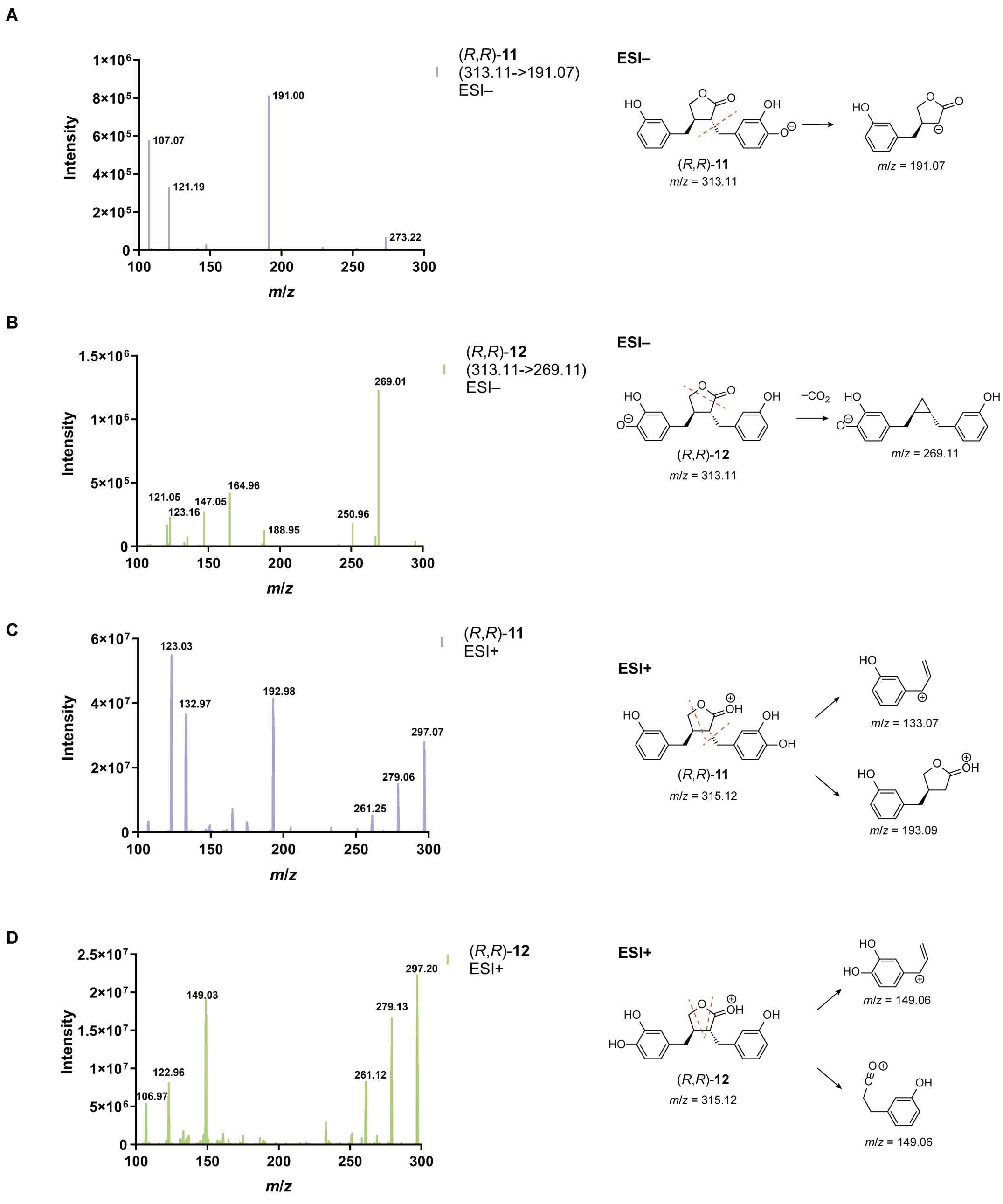

Figure S13. MS fragmentation of (*R*,*R*)-11 and (*R*,*R*)-12. A. LC–MS/MS fragmentation of (*R*,*R*)-**11** in negative ion mode which has a dominant daughter ion with *m*/*z* of 191.07 and a putative structure of the daughter ion derived from (*R*,*R*)-**11**. **B.** LC–MS/MS fragmentation of (*R*,*R*)-**12** in negative ion mode which has a dominant daughter ion with *m*/*z* of 269.11 and a putative structure of the daughter ion derived from (*R*,*R*)-**12**. **C.** LC–MS/MS fragmentation of (*R*,*R*)-**11** in positive ion mode which has two signature daughter ions with *m*/*z* of 193.09 and 133.07, respectively, and putative structures of the daughter ions derived from (*R*,*R*)-**11**, matching the previously reported fragmentation signature of this compound[9]. **D.** LC–MS/MS fragmentation of (*R*,*R*)-**12** in positive ion mode which has a signature daughter ion with *m*/*z* of 149.06, and a putative structure of the daughter ion derived from (*R*,*R*)-**12**, matching the previously reported fragmentation signature of this compound[9].

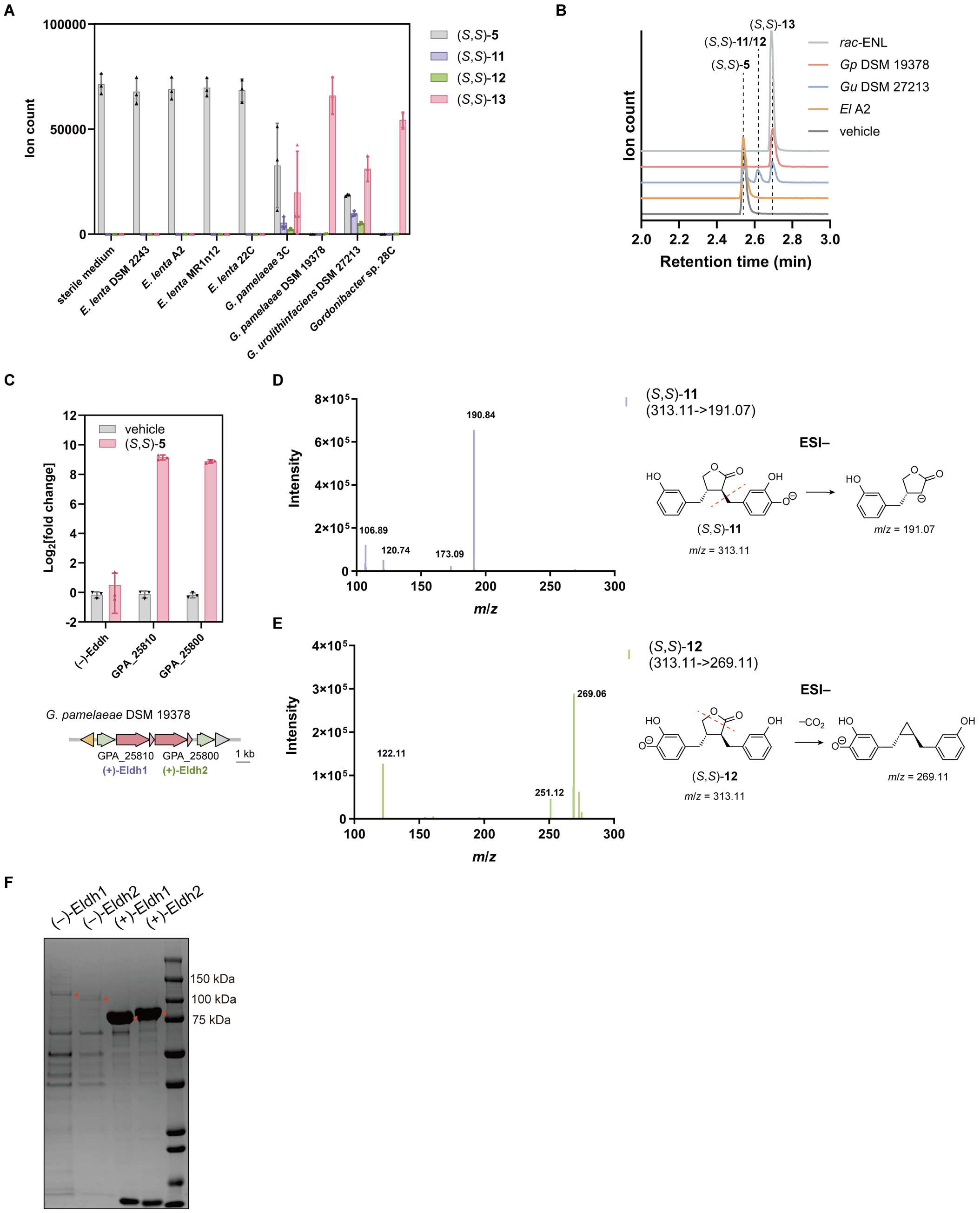

Figure S14. Metabolism of (*S*,*S*)-5 by different Coriobacteriia WT strains, identification of (*S*,*S*)-5 dehydroxylases and MS fragmentation of (*S*,*S*)-11 and (*S*,*S*)-12. A,**B.** LC–MS/MS quantification (**A**) and representative chromatogram traces (**B**) of the production of dehydroxylated metabolites (*S*,*S*)-**11**, (*S*,*S*)-**12** and (*S*,*S*)-**13** after incubation of (*S*,*S*)-**5** with different Coriobacteriia strains. **C.** RT-qPCR to test the expression level changes of GPA_25810, GPA_25800 and (–)-Eddh upon exposure to (*S*,*S*)-**5** from (+)-matairesinol CFS extract in *G. pamelaeae* DSM 19378, and schematic of GPA_25810 ((+)-Eldh1) and GPA_25800 ((+)-Eldh2) gene cluster. **D.** LC–MS/MS fragmentation of (*S*,*S*)-**11** which has a dominant daughter ion with *m*/*z* of 191.07 and a putative structure of the daughter ion derived from (*S*,*S*)-**11**. **E.** LC–MS/MS fragmentation of (*S*,*S*)-**12** which has a dominant daughter ion with *m*/*z* of 269.11 and a putative structure of the daughter ion derived from (*S*,*S*)-**12**. **F.** SDS-PAGE of purified (–)-Eldh1, (–)-Eldh2, (+)-Eldh1, and (+)-Eldh2 proteins.

Data represented as mean ± SD with n = 3 biological replicates in **A**. Data represented as mean ± S.E.M. with n = 3 biological replicates in **C**.

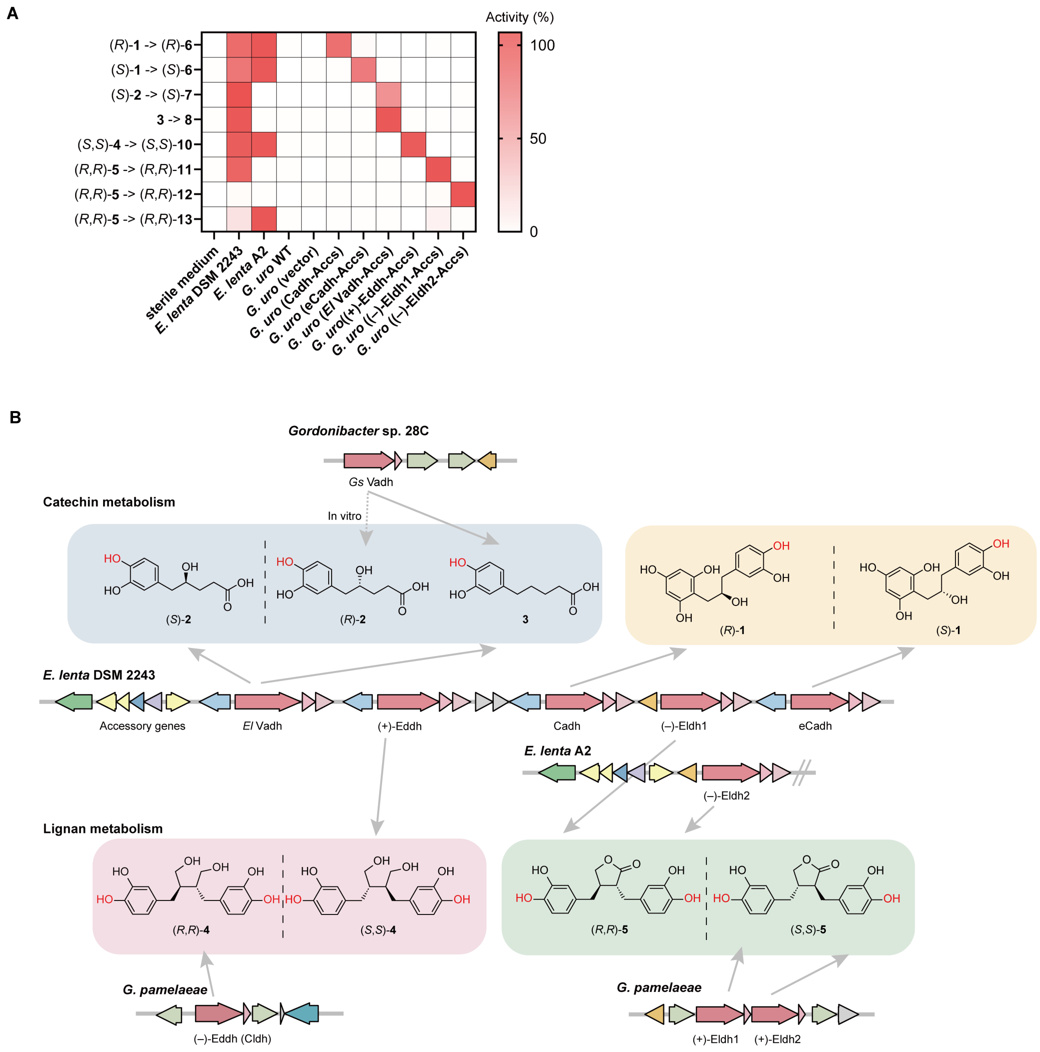

Figure S15. Substrate specificity of co-localized *E. lenta* catechol dehydroxylases, and summary of characterized catechol dehydroxylases in this study. A. LC–MS/MS data quantifying the dehydroxylated metabolites of (*R*)-**1**, (*S*)-**1**, (*S*)-**2**, **3**, (*S*,*S*)-**4**, and (*R*,*R*)-**5** after incubation with individual engineered *G. uro* strains to test substrate specificity. *E. lenta* DSM 2243 and A2 are positive controls and *G. uro* WT and *G. uro* (vector) are negative controls. **B.** Eight catechol dehydroxylases characterized in this study and two previously reported catechol dehydroxylases are involved in the gut bacterial metabolism of catechins and lignans. The substrate enantiomers are highlighted, and the arrows indicate the substrates of individual enzymes. Dashed line indicates that high activity is only detected in vitro. Data represented are means normalized to the highest intensity for each metabolite with n = 3 biological replicates in **A**.

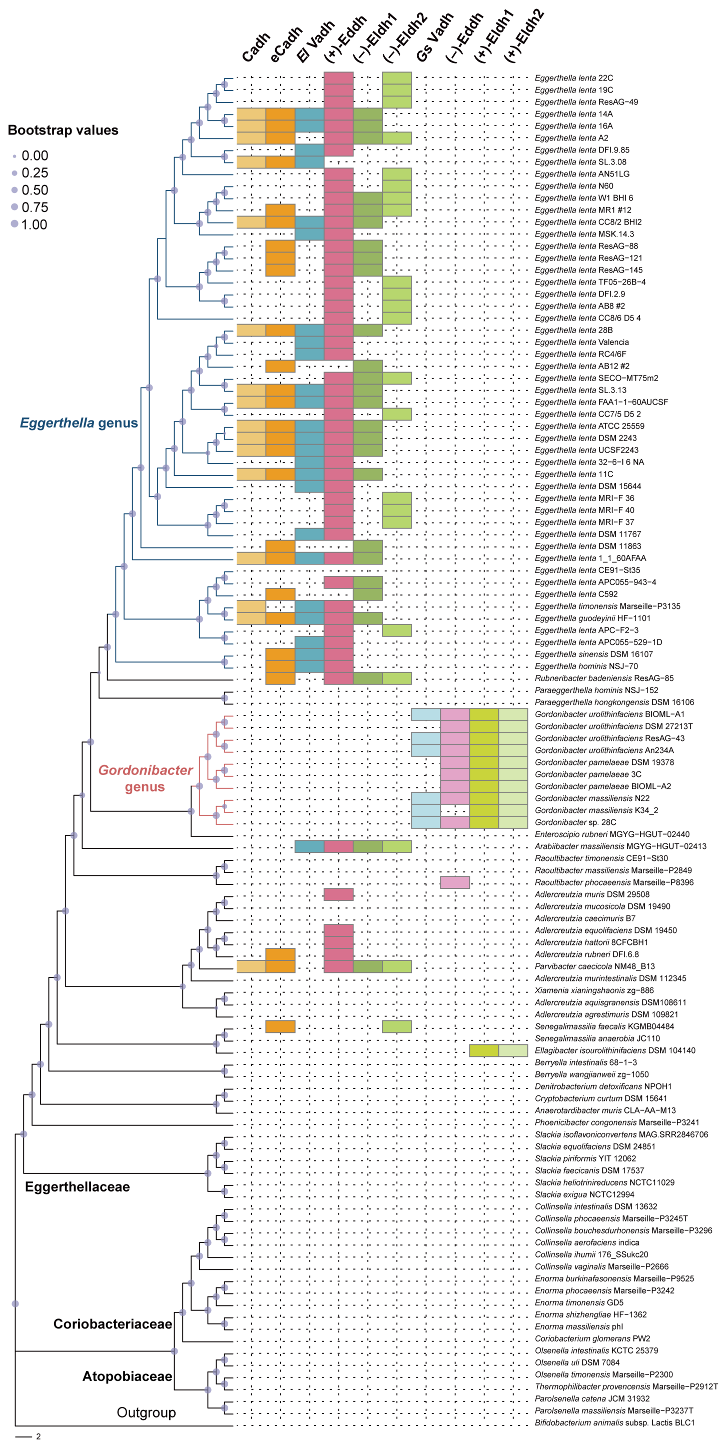

Figure S16. Distribution of catechol dehydroxylases in different Coriobacteriia strains with strain information specified. Phylogenetic analysis of Coriobacteriia genomes were performed using PhyloPhlAn v3.0. Distribution of the catechol dehydroxylases of interest in different Coriobacteriia genomes were analyzed by BLAST searches against a local database consisting of the 113 Coriobacteriia genomes. BLAST hits with coverage of over 85% and amino acid identity of over 80% for *Gs* Vadh or 75% for other enzyme queries were considered as enzyme homologs.

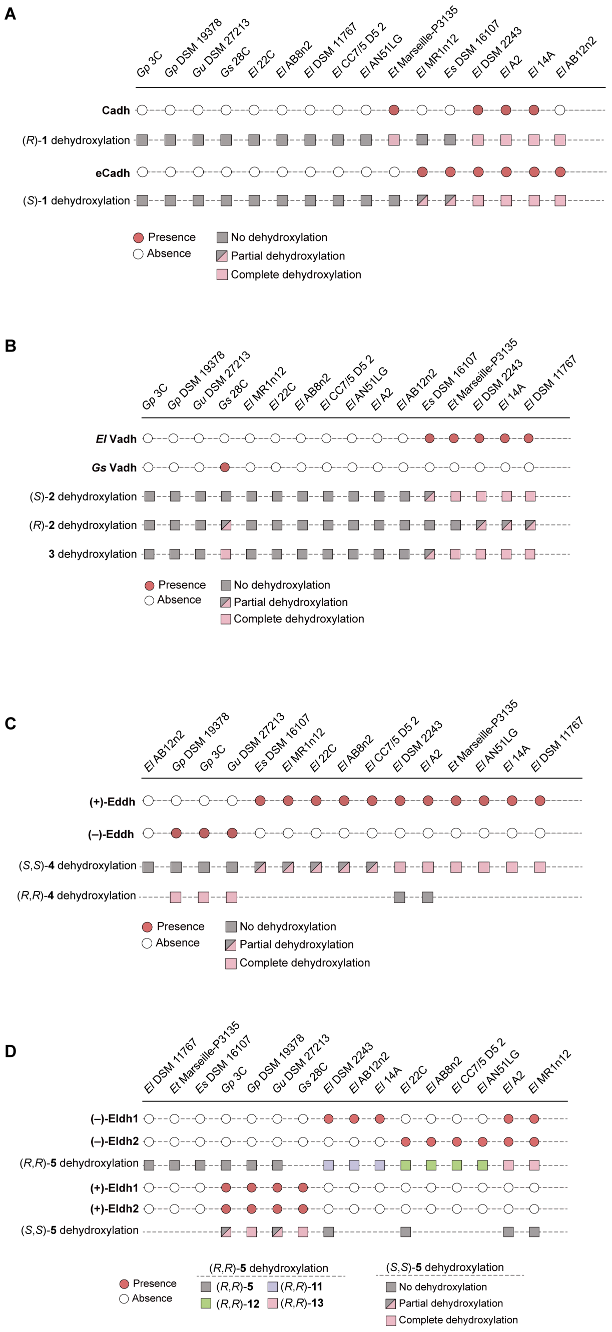

Figure S17. Presence of catechol dehydroxylases in different Coriobacteriia strains predicts the metabolism. A. Distribution of Cadh and eCadh in Coriobacteriia strains, and (*R*)-**1** and (*S*)-**1** dehydroxylation activity of Coriobacteriia strains. **B.** Distribution of *El* Vadh and *Gs* Vadh in Coriobacteriia strains, and (*S*)-**2**, (*R*)-**2**, and **3** dehydroxylation activity of Coriobacteriia strains. **C.** Distribution of (+)-Eddh and (–)-Eddh in Coriobacteriia strains, and (*S*,*S*)-**4** and (*R*,*R*)-**4** dehydroxylation activity of Coriobacteriia strains. **D.** Distribution of (–)-Eldh1, (–)-Eldh2, (+)-Eldh1, and (+)-Eldh2 in different Coriobacteriia strains, and (*R*,*R*)-**5** and (*S*,*S*)-**5** dehydroxylation activity of Coriobacteriia strains.

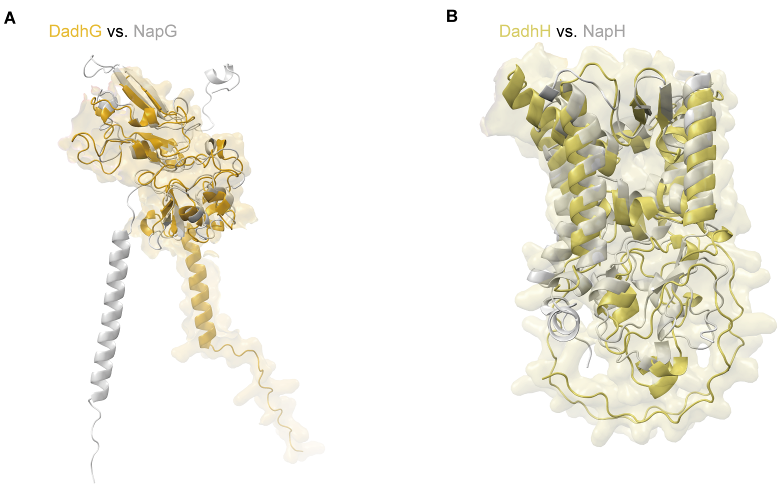

Figure S18. DadhG/H share similar structures to NapG/H. A. Alignment of the predicted structures of DadhG and NapG. **B.** Alignment of the predicted structures of DadhH and NapH.

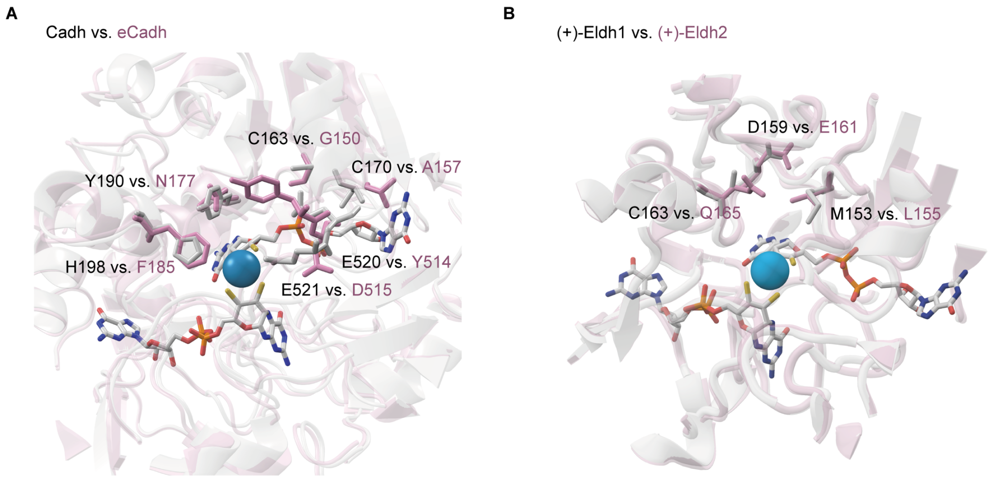

Figure S19. Active site comparison of enantiocomplementary and complementary site-selective catechol dehydroxylases. A. Alignment of the predicted active site structures of enantiocomplementary enzymes Cadh and eCadh. **B.** Alignment of the predicted active site structures of complementary site-selective enzymes (+)-Eldh1 and (+)-Eldh2.

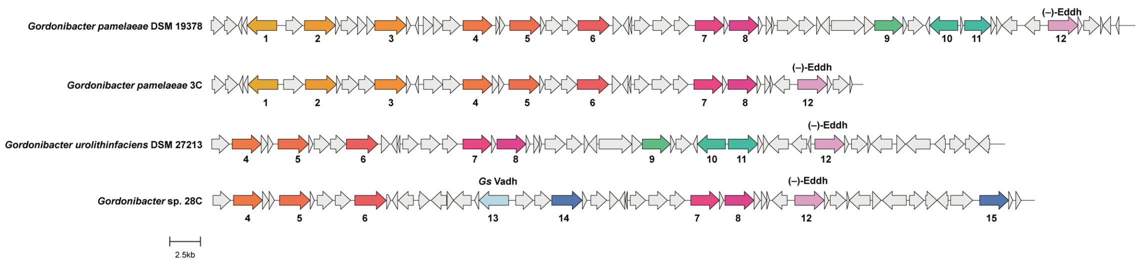

Figure S20. Hypervariable genomic regions encoding catechol dehydroxylases in different *Gordonibacter* strains. Multiple uncharacterized catechol dehydroxylases are encoded close to (–)-Eddh in a highly variable manner across different *Gordonibacter* strains.

Supplemental references

(1) Dong, X.; Guthrie, B. G. H.; Alexander, M.; Noecker, C.; Ramirez, L.; Glasser, N. R.; Turnbaugh, P. J.; Balskus, E. P. Genetic manipulation of the human gut bacterium Eggerthella lenta reveals a widespread family of transcriptional regulators. *Nat Commun* **2022**, *13* (1), 7624.
(2) Maini Rekdal, V.; Nol Bernadino, P.; Luescher, M. U.; Kiamehr, S.; Le, C.; Bisanz, J. E.; Turnbaugh, P. J.; Bess, E. N.; Balskus, E. P. A widely distributed metalloenzyme class enables gut microbial metabolism of host- and diet-derived catechols. *Elife* **2020**, *9*, e50845.
(3) Takagaki, A.; Nanjo, F. Catabolism of (+)-catechin and (-)-epicatechin by rat intestinal microbiota. *J Agric Food Chem* **2013**, *61* (20), 4927-4935.
(4) Takagaki, A.; Nanjo, F. Bioconversion of (−)-Epicatechin, (+)-Epicatechin, (−)-Catechin, and (+)-Catechin by (−)-Epigallocatechin-Metabolizing Bacteria. *Biological and Pharmaceutical Bulletin* **2015**, *38* (5), 789-794.
(5) Maini Rekdal, V.; Bess, E. N.; Bisanz, J. E.; Turnbaugh, P. J.; Balskus, E. P. Discovery and inhibition of an interspecies gut bacterial pathway for Levodopa metabolism. *Science* **2019**, *364* (6445), eaau6323.
(6) Jin, J. S.; Zhao, Y. F.; Nakamura, N.; Akao, T.; Kakiuchi, N.; Min, B. S.; Hattori, M. Enantioselective dehydroxylation of enterodiol and enterolactone precursors by human intestinal bacteria. *Biol Pharm Bull* **2007**, *30* (11), 2113-2119.
(7) Bess, E. N.; Bisanz, J. E.; Yarza, F.; Bustion, A.; Rich, B. E.; Li, X.; Kitamura, S.; Waligurski, E.; Ang, Q. Y.; Alba, D. L.; et al. Genetic basis for the cooperative bioactivation of plant lignans by Eggerthella lenta and other human gut bacteria. *Nat Microbiol* **2020**, *5* (1), 56-66.
(8) Xie, L. H.; Akao, T.; Hamasaki, K.; Deyama, T.; Hattori, M. Biotransformation of pinoresinol diglucoside to mammalian lignans by human intestinal microflora, and isolation of Enterococcus faecalis strain PDG-1 responsible for the transformation of (+)-pinoresinol to (+)-lariciresinol. *Chem Pharm Bull (Tokyo)* **2003**, *51* (5), 508-515.
(9) Jin, J. S.; Hattori, M. Further studies on a human intestinal bacterium Ruminococcus sp. END-1 for transformation of plant lignans to mammalian lignans. *J Agric Food Chem* **2009**, *57* (16), 7537-7542.
(10) Asnicar, F.; Thomas, A. M.; Beghini, F.; Mengoni, C.; Manara, S.; Manghi, P.; Zhu, Q.; Bolzan, M.; Cumbo, F.; May, U.; et al. Precise phylogenetic analysis of microbial isolates and genomes from metagenomes using PhyloPhlAn 3.0. *Nat Commun* **2020**, *11* (1), 2500.
(11) Yu, G.; Smith, D. K.; Zhu, H.; Guan, Y.; Lam, T. T. Y.; McInerny, G. ggtree: an r package for visualization and annotation of phylogenetic trees with their covariates and other associated data. *Methods in Ecology and Evolution* **2016**, *8* (1), 28-36.
(12) Gilchrist, C. L. M.; Chooi, Y. H. clinker & clustermap.js: automatic generation of gene cluster comparison figures. *Bioinformatics* **2021**, *37* (16), 2473-2475.
(13) Zallot, R.; Oberg, N.; Gerlt, J. A. The EFI Web Resource for Genomic Enzymology Tools: Leveraging Protein, Genome, and Metagenome Databases to Discover Novel Enzymes and Metabolic Pathways. *Biochemistry* **2019**, *58* (41), 4169-4182.
(14) Katoh, K.; Standley, D. M. MAFFT multiple sequence alignment software version 7: improvements in performance and usability. *Mol Biol Evol* **2013**, *30* (4), 772-780.
(15) Capella-Gutierrez, S.; Silla-Martinez, J. M.; Gabaldon, T. trimAl: a tool for automated alignment trimming in large-scale phylogenetic analyses. *Bioinformatics* **2009**, *25* (15), 1972-1973.
(16) Minh, B. Q.; Schmidt, H. A.; Chernomor, O.; Schrempf, D.; Woodhams, M. D.; von Haeseler, A.; Lanfear, R. IQ-TREE 2: New Models and Efficient Methods for Phylogenetic Inference in the Genomic Era. *Mol Biol Evol* **2020**, *37* (5), 1530-1534.
(17) Letunic, I.; Bork, P. Interactive Tree Of Life (iTOL) v5: an online tool for phylogenetic tree display and annotation. *Nucleic Acids Res* **2021**, *49* (W1), W293-W296.
(18) Abramson, J.; Adler, J.; Dunger, J.; Evans, R.; Green, T.; Pritzel, A.; Ronneberger, O.; Willmore, L.; Ballard, A. J.; Bambrick, J.; et al. Accurate structure prediction of biomolecular interactions with AlphaFold 3. *Nature* **2024**, *630* (8016), 493-500.
(19) van Kempen, M.; Kim, S. S.; Tumescheit, C.; Mirdita, M.; Lee, J.; Gilchrist, C. L. M.; Soding, J.; Steinegger, M. Fast and accurate protein structure search with Foldseek. *Nat Biotechnol* **2024**, *42* (2), 243-246.
